# High-Throughput Development of SSR Markers from Pea (*Pisum sativum* L.) Based on Next Generation Sequencing of a Purified Chinese Commercial Variety

**DOI:** 10.1371/journal.pone.0139775

**Published:** 2015-10-06

**Authors:** Tao Yang, Li Fang, Xiaoyan Zhang, Jinguo Hu, Shiying Bao, Junjie Hao, Ling Li, Yuhua He, Junye Jiang, Fang Wang, Shufang Tian, Xuxiao Zong

**Affiliations:** 1 The National Key Facility for Crop Gene Resources and Genetic Improvement/Institute of Crop Science, Chinese Academy of Agricultural Sciences, Beijing, China; 2 Qingdao Academy of Agricultural Sciences, Qingdao, China; 3 USDA-ARS Western Regional Plant Introduction Station, Pullman, Washington, United States of America; 4 Institute of Grain Crops, Yunnan Academy of Agricultural Sciences, Kunming, China; 5 Institute of Cash Crops, Liaoning Academy of Agricultural Sciences, Liaoyang, China; University of New England, AUSTRALIA

## Abstract

Pea (*Pisum sativum* L.) is an important food legume globally, and is the plant species that J.G. Mendel used to lay the foundation of modern genetics. However, genomics resources of pea are limited comparing to other crop species. Application of marker assisted selection (MAS) in pea breeding has lagged behind many other crops. Development of a large number of novel and reliable SSR (simple sequence repeat) or microsatellite markers will help both basic and applied genomics research of this crop. The Illumina HiSeq 2500 System was used to uncover 8,899 putative SSR containing sequences, and 3,275 non-redundant primers were designed to amplify these SSRs. Among the 1,644 SSRs that were randomly selected for primer validation, 841 yielded reliable amplifications of detectable polymorphisms among 24 genotypes of cultivated pea (*Pisum sativum* L.) and wild relatives (*P*. *fulvum* Sm.) originated from diverse geographical locations. The dataset indicated that the allele number per locus ranged from 2 to 10, and that the polymorphism information content (PIC) ranged from 0.08 to 0.82 with an average of 0.38. These 1,644 novel SSR markers were also tested for polymorphism between genotypes G0003973 and G0005527. Finally, 33 polymorphic SSR markers were anchored on the genetic linkage map of G0003973 × G0005527 F_2_ population.

## Introduction

Pea (*Pisum sativum* L.) is one of the most popular food legumes in the world. The harvested area was approximately 6.4 million hectares and production was almost 11 million metric tons of dry peas in 2013 [1]. As one of the most important legumes, pea can be used as vegetable, pulse, and feed. Moreover, pea plays a critical role in crop rotation and low-carbon agriculture for its capacity of biological fixation of atmospheric N_2_ [2].

Although significant advances have been made through traditional breeding practices, resulting in semi-leafless pea, snow pea, and snap pea, progress in developing SSR markers [3–6] and marker assisted selection in pea breeding is limited. This is due mainly to the large genome size of pea (4.45 GB), which is approximately 9 times larger than that of barrel medic (*Medicago truncatula* Gaertn.) (http://www.jcvi.org/medicago/), and 4 times larger than that of soybean (*Glycine max* L. Merr.) [7].

A number of next-generation sequencing technologies such as the Roche 454, the Illumina Hiseq 2500 and the Pacific Biosciences PacBio RS II systems have been developed in recent years. These technologies are capable of generating tens of millions of short DNA sequence reads at a relatively low cost. De novo sequencing of genomes, re-sequencing of genomes and RNA-seq were popular all over the world [8–10]. However, only a few researchers utilized Next Generation Sequencing (NGS) platforms for high-throughput development of SSR markers in plant genome [11–16].

The present study aims at obtaining more SSR sequences cheaply and efficiently by using the high-throughput Illumina HiSeq 2500 platform (Illumina, San Diego, CA, USA). We report here the result of identifying over 8,899 putative SSR containing sequences, characterizing and validating 1,644 of these newly identified SSRs experimentally using 22 *P*. *sativum* and two *P*. *fulvum* genotypes, and enhancing the density of previous genetic linkage map with 33 of these newly identified markers.

## Materials and Methods

### Plant materials

Widely grown Chinese pea cultivar Zhongwan No. 6, numbered G0005527 in the National Genebank of China, was purified by single seed descend for three consecutive generations. DNA from the resulting plants was used for sequencing and SSR marker development.

**Fig 1:**
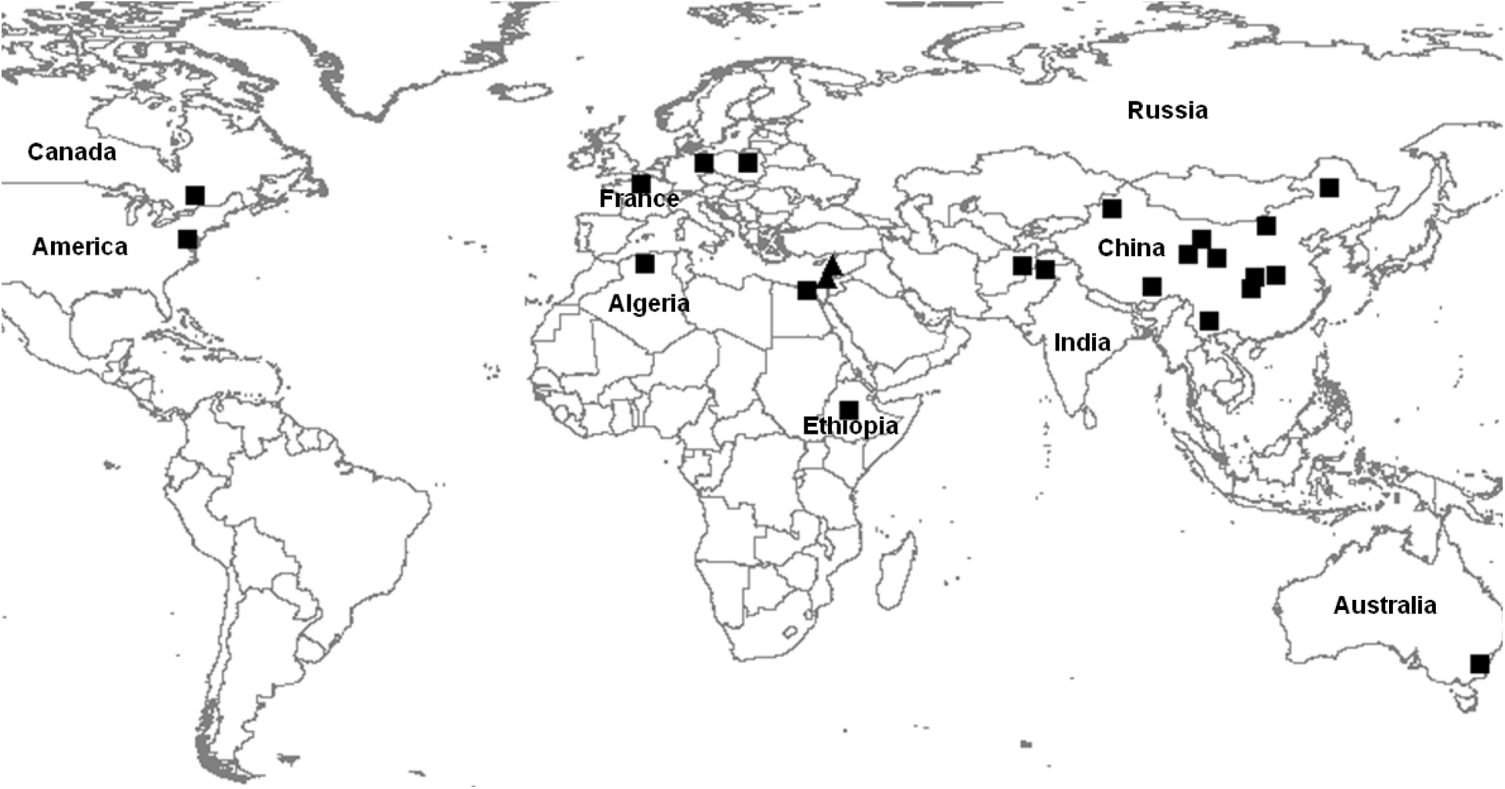
Geographic distribution of the 24 entries in the diverse panel for putative SSR validation (*Pisum sativum* entries are represented with squares and *Pisum fulvum* triangles).

For validating the SSRs, a diverse panel of 24 accessions, consisting of 11 entries from China, 11 from other countries and two wild relatives as out-groups, was used in the amplification experiment (Fig 1 and Table 1). These germplasm resources are maintained by the National Genebank of China at the Institute of Crop Science (ICS), Chinese Academy of Agricultural Sciences (CAAS), Beijing, China.

**Table 1:** Country of origin and geographic information of the 24 pea and its wild relatives used in this study.

	Voucher number	Species	Collection locale	Longitude	Latitude
1	G0000809	*Pisum sativum*	USA.	-164.5000	63.2500
2	G0000831	*Pisum sativum*	France	2.3330	48.8330
3	G0000868	*Pisum sativum*	Australia	149.1330	-35.2500
4	G0000871	*Pisum sativum*	Egypt	31.2330	30.0170
5	G0002101	*Pisum sativum*	Canada	-75.7000	45.4500
6	G0002108	*Pisum sativum*	Poland	21.0000	52.2170
7	G0002860	*Pisum sativum*	Germany	13.4170	52.5000
8	G0004353	*Pisum sativum*	Ethiopia	38.7000	9.0330
9	G0004484	*Pisum sativum*	Algeria	3.1330	36.7000
10	G0006082	*Pisum sativum*	Afghanistan	69.1830	34.4670
11	G0006170	*Pisum sativum*	Pakistan	73.1670	33.6670
12	G0000001	*Pisum sativum*	Henan, China	113.3200	32.7200
13	G0000043	*Pisum sativum*	Yunnan, China	101.6419	24.6889
14	G0000557	*Pisum sativum*	Xinjiang, China	84.8000	44.4000
15	G0000673	*Pisum sativum*	Qinghai, China	98.0872	36.2978
16	G0000783	*Pisum sativum*	Gansu, China	100.5070	38.9483
17	G0002288	*Pisum sativum*	Gansu, China	103.2105	35.6012
18	G0002305	*Pisum sativum*	Inner Mongolia, China	122.7375	48.0137
19	G0002371	*Pisum sativum*	Tibet, China	92.47	28.4200
20	G0002654	*Pisum sativum*	Inner Mongolia, China	111.6800	41.3700
21	G0003219	*Pisum sativum*	Hubei, China	108.9364	30.2910
22	G0003268	*Pisum sativum*	Hubei, China	109.7153	32.3183
23	G0005094	*Pisum fulvum*	Israel	35.2000	31.7830
24	G0005733	*Pisum fulvum*	Israel	34.4600	32.0500

For SSR mapping, a segregating F_2_ population of 190 individuals derived from the cross of G0003973 × G0005527 was used. The dry seed color of G0003973 (winter hardy, from Qinghai) was olivine and that of G0005527 (cold sensitive, from Beijing) was green. This population was grown in a protected field at Qingdao Academy of Agricultural Sciences, Qingdao (QdAAS), Shandong, China.

All the plant materials were planted in the protected field of experimental farm within CAAS campus (39° 57' 38" N, 116° 19' 27" E).

### DNA extraction, library preparation and next-generation sequencing

Genomic DNA was extracted from 10-day old, etiolated seedlings of each genotype cleared with sterile water, using the CTAB method [17,18]. For the Illumina HiSeq 2500 run, a library was prepared with a commercial kit NEBNext Multiplex Oligos for Illumina with Index Primers Set 2 (New England Biolabs Inc., Ipswich, MA, USA) following the manufacturer’s protocol (Paired-End Library Construction). The raw sequencing files were submitted to the National Center for Biotechnology Information (NCBI) short read archive under accession numbers with the accession number SRX973821.

### Reads initiative characterization

CLC Genomics Workbench 7.5 software (CLC Inc., Aarhus, Denmark) was used in the following analyses. The quality of paired-end data was checked by the Create Sequencing QC Report Module at default parameters. Subsequent quality trimming was performed with the Trim Sequences Module using quality scores limit of 0.05 and maximum number of ambiguities of 2. The Remove of Duplicate Reads Module was used to filter redundant reads at default parameters. Finally, de novo Assembly Module was used for sequences assembly. These sequences were prepared for further SSRs mining.

### SSRs mining

MISA (Microsatellite identification) software, a SSRs motif scanning tool written in Perl (http://pgrc.ipk-gatersleben.de/misa/), was used for the identification and localization of SSRs or microsatellites. The identified motifs were mononucleotide to hexanucleotide, and the minimum repeat unit was defined as 10 for mononucleotide, 6 for dinucleotide, 5 for all the higher order motifs including trinucleotide, tetranucleotide, pentanucleotide and hexanucleotide. Furthermore, the maximal number of interrupting base pairs in a compound microsatellite was 20 bp. The characterizations of SSRs were obtained by statistical analysis from the MISA files. The SSRs information was extracted and statistically analyzed by in-house Perl script, plotted by R language [19].

### Primer design

The high throughput primers designing pipeline contained Perl scripts p3_in.pl, p3_out.pl (http://pgrc.ipk-gatersleben.de/misa/primer3.html) and Primer 3.0 software (http://www-genome.wi.mit.edu/genome_software/other/primer3.html). Redundant primers were removed by the in-house developed script: reduce_ssr.py (data in S1 File), and the ‘fine’ primers were used for further study.

### PCR amplification

Polymerase chain reactions (PCR) were performed in 10 μl reaction volumes containing 5 μl 2 x TaqPCR MasterMix (Hooseen, Beijing, China), 1 μl primer pair (10 μM), 1.5 μl of genomic DNA (30 ng) and 2.5 μl of dd H_2_O. Microsatellites were amplified on a K960 Thermal Cycler (Jingle, Hangzhou, China) with the following cycle: 5 min initial denaturation at 95°C, 35 cycles of 30 s at 95°C, 30 s at the optimized annealing temperature, 45 s of elongation at 72°C, and a final extension at 72°C for 10 min. The PCR products were separated on 8% non-denaturing polyacrylamide gel electrophoresed at 280 V and 50 W and visualized by 0.1% silver nitrate staining.

### Polymorphic validation and genetic diversity assessment

The number of alleles and polymorphism information content (PIC) of the alleles revealed by each primer pair were calculated by Powermarker v3.25 [20] with the genotype data among 24 accessions. A cluster analysis was conducted based on the unweighted pair group method on arithmetic averages (UPGMA) algorithm using Powermarker v3.25, and a dendrogram was drawn by Powermarker v3.25 [20] and modified by MEGA4 [21].

STRUCTURE V2.3.3 was used to analyze population structure and differentiation [22,23]. Simulations were run with a burn-in of 100,000 iterations and from K (the number of populations) = 1 to 10. Runs for each K were replicated 160 times and the true K was determined according to the method described by Evanno [24].

### Linkage map construction and blast mapped SSR markers to *Medicago truncatula*

The distorted segregation of the markers against the expected Mendelian segregation ratio was tested with Chi-squared analysis (P < 0.05) by QTL ICIMapping V3.2 software [25]. The information of SSR markers were filled into Map Manager QTXb 20 software [26]. For the F_2_ population, the male allele was recorded as “A” and the female allele as “B”, “H” was recorded when a locus was heterozygous, and “-” when there was a missing or null allele. The linkage map was constructed using the Map Manager QTXb 20 software with the parameter of Kosambi function (P < 0.0001) and marker distances in centiMorgans (cM). Finally, the linkage map was presented by JoinMap 4.0 software [27]. Putative location of flanking sequences of mapped SSRs onto chromosomes of *Medicago truncatula* for synteny-based comparison was conducted by using blast method (http://phytozome.jgi.doe.gov/pz/portal.html#!search?show=BLAST&method=Org_Mtruncatula).

## Results

### Illumina paired-end sequencing

In this study, a total of 17.5 GB of paired-end raw sequencing data, comprising 173,245,234 reads from a 500 bp insert DNA library, was generated by Illumina Hiseq2500 system. After trimming the adaptors and removal of possible contaminations, the remaining 170,865,238 high quality read sequences were used for further analysis. Adenine was the most abundant type, accounting for 29.1% of total nucleotides, followed by thymine (28.9%), cytosine (21.0%) and guanine (21.0%). The CG content was about 42% and the average read length was 94.7 bp.

### Duplicated reads removing and genome de novo assembling

The trimmed reads were used for duplicated sequences analysis under the Remove Duplicate Reads Module in CLC Genomics Workbench 7.5 software. As a result, there were 505,740 (0.3%) duplicate reads and 170,359,498 (99.7%) unique ones. After the de novo assembly, the number of contigs (including scaffolded regions) was 343,849. The average length of contigs and the N50 was about 370 bp and 359 bp, respectively.

### Mining for SSRs

MISA software was used for SSRs search based on contigs. The total number of SSR containing sequences was 8,899, and these sequences contained 10,207 SSRs (Table 2). In this study, mono- and di- nucleotide motifs occurred at the highest rate (accounting for 40.86% and 32.68%, respectively). Trinucleotide motifs accounted for 25.29%, while tetra-, penta-, and hexa-nucleotide motifs accounted for 1.17%. (A/T)_n_, (AC/GT)_n_ and (AG/CT)_n_ were the relatively more frequent motifs in our study.

**Table 2:** MISA result in this study.

Category	Numbers
Total number of sequences examined	343,849
Total size of examined sequences (bp)	127,283,564
Total number of identified SSRs	10,207
Number of SSR containing sequences	8,899
Number of sequences containing more than one SSR	671
Number of SSRs present in compound formation	450

### Primer design

A total of 3,275 non-redundant primer pairs were designed by Primer 3.0 software and reduce_ssr.py (in house developed programs) based on criteria of melting temperature, CG content, lack of secondary structure and length of amplification bands. The expected length of target bands was between 110 bp and 210 bp.

### Validation of the SSR markers

**Fig 2:**
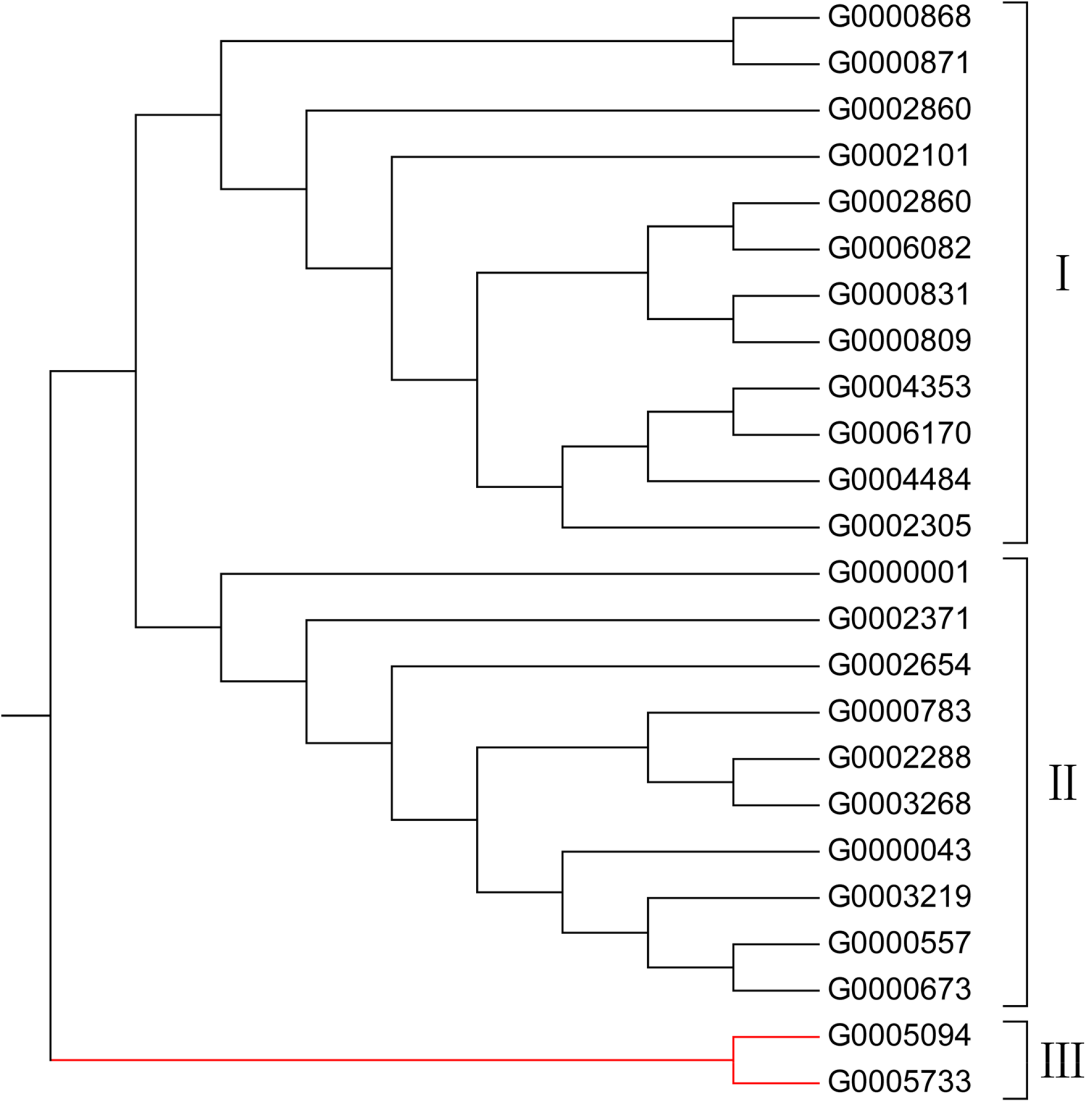
A UPGMA dendrogram of 24 accessions of pea and its wild species based on 841 polymorphic SSR markers amplified (Cluster I consisted of overseas accessions except G0002305; Cluster II consisted Chinese accessions; Cluster III consisted of wild relatives).

A subset of 1,644 SSR markers was randomly selected for validation. Among them 841 (51.16%) markers (S2 File) produced reliable polymorphic bands between 22 pea accessions (*Pisum sativum*) and two wild relatives (*Pisum fulvum*). Meanwhile, the monomorphic markers were listed in S3 File. The allele number per locus ranged from 2 to 10 with an average of 3.22. The polymorphism information content (PIC) with an average of 0.38, ranged from 0.08 to 0.82 (S2 File). The dendrogram clearly showed that the 24 pea and its wild relative accessions fell into three distinct clusters based on 841 polymorphic SSR markers (Fig 2). Cluster I consisted of overseas accessions except G0002305; Cluster II consisted Chinese accessions; Cluster III consisted of wild relatives.

**Fig 3:**
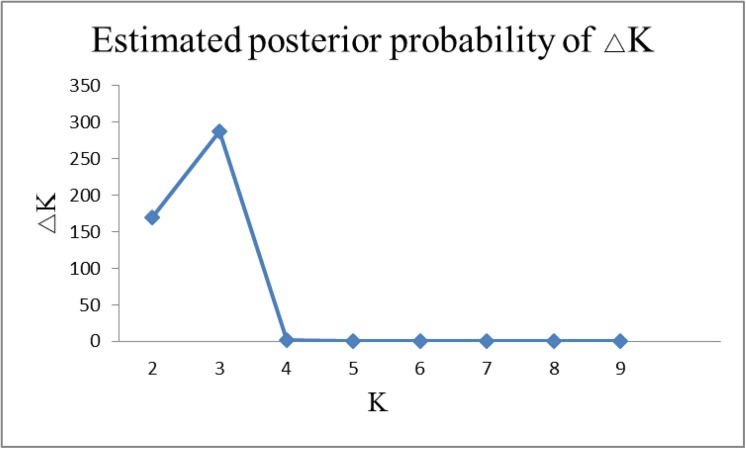
ΔK was used to determine the most appropriate K value for population structure.

**Fig 4:**
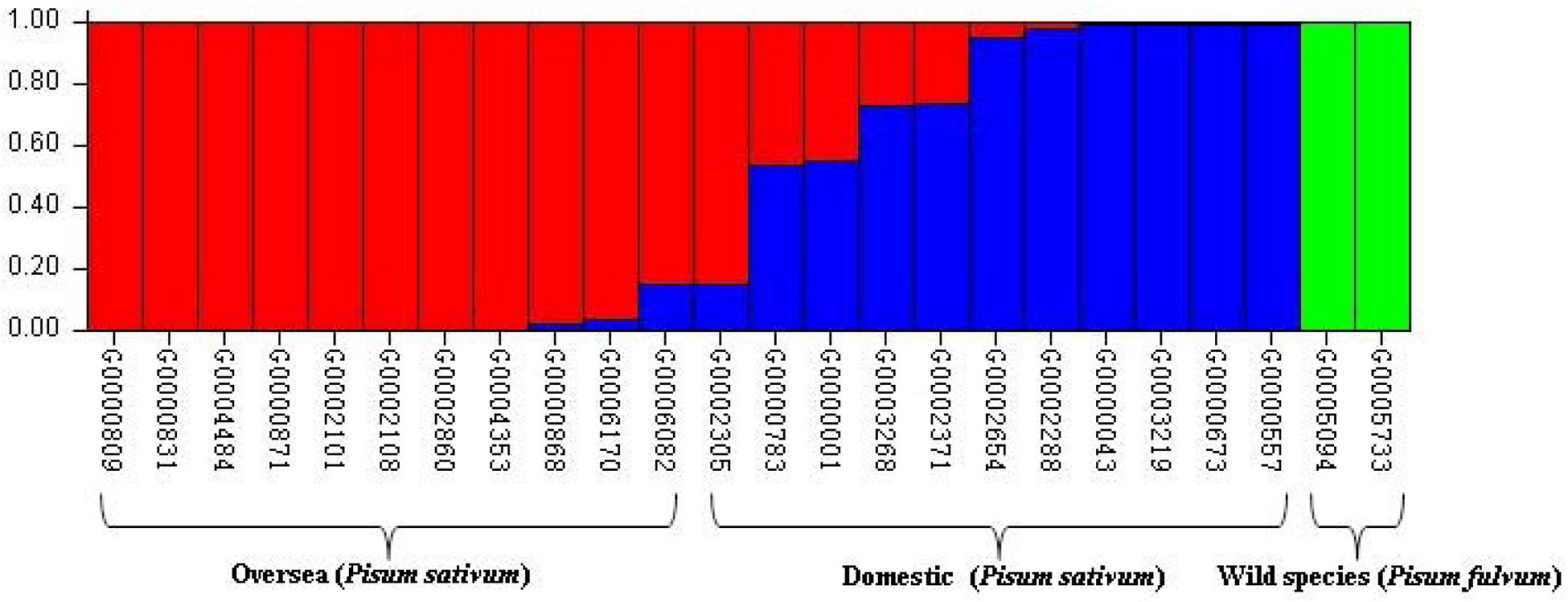
Population structure of K = 3 inferred by Bayesian clustering approaches based on SSR markers.

The population structure of this diverse panel of cultivated pea and its wild relative was inferred by using STRUCTURE V2.3.3 with the dataset of 841 SSR markers. Three sub-populations were identified, based on ΔK values (Fig 3, [24]). The rational for thisΔK is to make salient the break in slope of the distribution of *L*(K) at the true K. The entries from China, from other countries and the wild species were separated into 3 sub-populations (Fig 4), in good according with the three clusters in the UPGMA dendrogram. The results were in accordance with those published earlier [28].

### Using novel SSR markers to enhance the density of genetic linkage map

**Fig 5:**
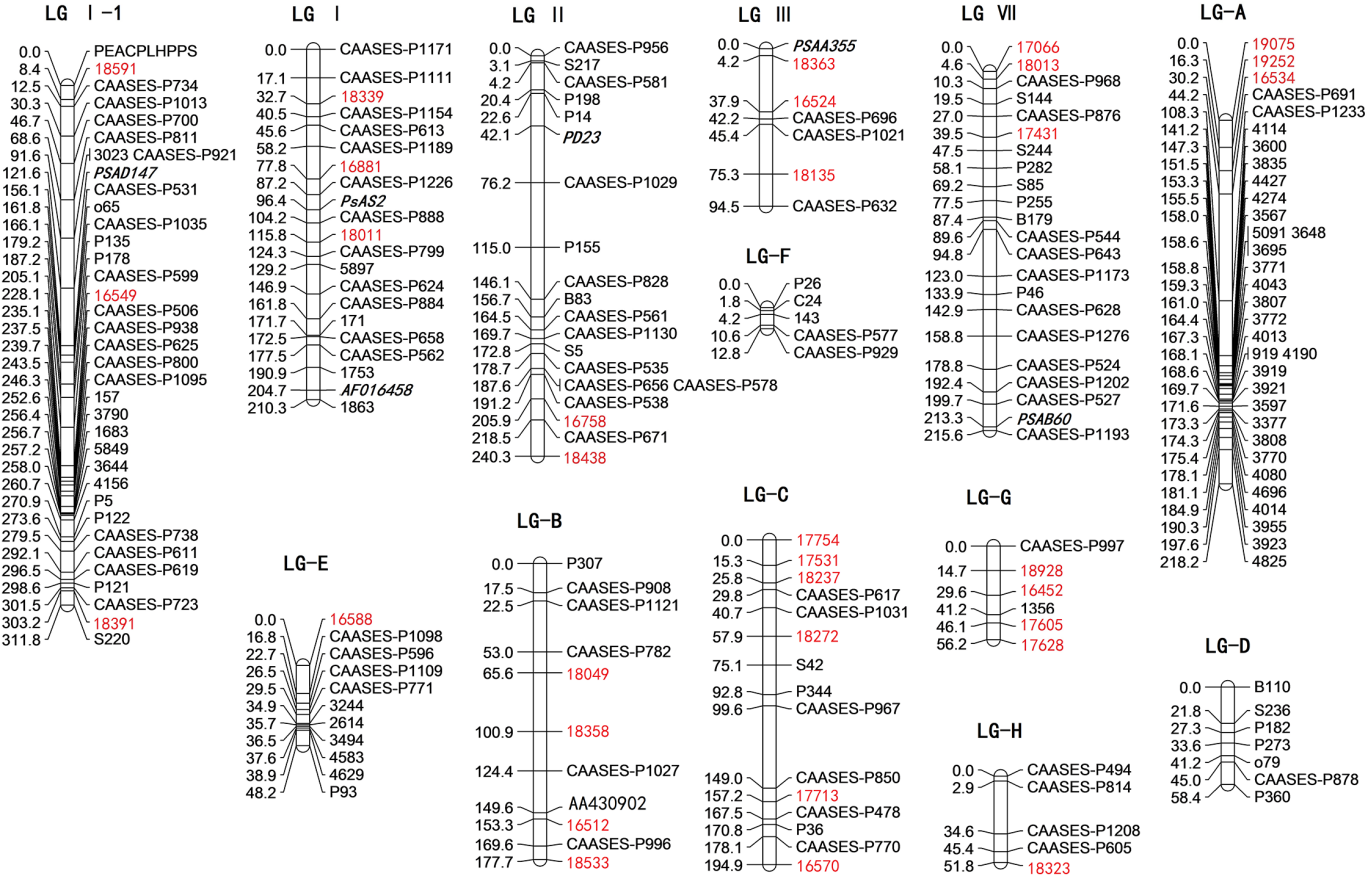
The enhanced genetic linkage map of the G0003973 × G0005527 F_2_ population with newly developed SSR markers (red).

A segregating F_2_ population derived from the cross between G0003973 and G0005527 was used for mapping the newly validated SSR markers. Among the 1,644 SSRs used in genetic diversity analysis, 63 were polymorphic between the two parents. Being amplified in the population, 22 of the 63 SSRs showed significant segregation distortion (P < 0.05) in S4 File. These distorted markers were excluded from linkage map construction. The Map Manager QTXb 20 was used to add the newly developed SSR markers to the genetic linkage map which had been published [29]. Consequently, 41 polymorphic markers that segregated in appropriate Mendalian ratios were used to run Map Manager QTXb 20 software, of which 33 markers were mapped to the existing linkage groups. However, the remaining eight markers were not linked to any mapped markers on the linkage map. The new map contained 199 markers including the 33 newly added markers (Table 3) in 13 linkage groups with an average genetic distance of 9.5 cM between neighboring markers and covered 1890.88 cM (Fig 5).

**Table 3:** Primer sequence and linkage group information of the 33 mapped SSR markers.

No.	Marker Name	Primer sequences(5’-3’)	Linkage Group	Map Positions(cM)	*Medicago truncatula* Chromosome	E-Value
1	18339	F:TGGTTGAACTGGAACGAGTG R:TGAAATTGCAATGTAAGCATGA	LG Ⅰ	32.67	Chr 5	8.9E-20
2	16881	F:ATGGGCTTTAGGGGAAGAAA R:AAAAGCAGCACATGGAGGAC	LG Ⅰ	77.78	Chr 5	7.1E-02
3	18011	F:GACCAACGACTTGGACATCA R:GGTGAGTTCCTAAGATGAATCAGA	LG Ⅰ	115.80	Not found	Not found
4	18591	F:AGGGCCGAATGCTAAGTGAT R:TTTTGAACCCTGGAGGGAGT	LG Ⅰ-1	8.44	Chr 5	1.3E-18
5	16549	F:CAATGAGATGCTGGCGATAA R:GTTCGGTGTTGTGGGTTTTT	LG Ⅰ-1	228.11	Chr 4	9.8E-07
6	18391	F:CCATCCTCCACGTGTCTCTT R:TCGCATATCCAAATGCAAAC	LG Ⅰ-1	303.15	Chr 7	3.3E-19
7	16758	F:CCCTTCAACAAAGCCTAACG R:AGGGTGCGAAGGAGGTTAGT	LG Ⅱ	205.92	Chr 6	2.7E-06
8	18438	F:GATTGAGCCGTGCCAATATC R:GATCCCACCCTAGAGGAAAAA	LG Ⅱ	240.35	Chr 4	5.7E-10
9	18363	F:CATGCATGGAGTTGGAAGAG R:GTCCCAAAATGCAGCCAATA	LG Ⅲ	4.22	Chr 2	9.1E-20
10	16524	F:CCAGAGGATGTGAACCAGGTA R:TTCAACCAAGCTGAACCCTTA	LG Ⅲ	37.88	Chr 6	2.1E-02
11	18135	F:CTTCAACCAACTGCGAGTGA R:TCATTTGAGTTTTGCCATGTTC	LG Ⅲ	75.26	Chr 3	2.2E-20
12	17066	F:TGGGATGAAAATGTTATGAATG R:CAAAACCACCCTTTCCGATA	LG Ⅶ	0.00	Chr 8	3.2E-20
13	18013	F:TCAATTCCGAACCACCTTTC R:CGGCAGAATTAGGGTTTTGA	LG Ⅶ	4.60	Chr 8	1.8E-05
14	17431	F:TTCACAATTCACCACCAATCA R:CCAACGTCAGGTACGATTCA	LG Ⅶ	39.48	Chr 1	9.5E-03
15	19075	F:CACGAGTACAACATGGAGTGAAG R:CAAGCTCAACCTCCTCATACC	LG A	0.00	Chr 6	3.1E-02
16	19252	F:CAATATTGATCGGAATTTGTTTC R:TGCGGTTTGATTGAGTTTGA	LG A	16.34	Chr 7	6.8E-11
17	16534	F:TTGCAAATATACCAATTCCAAAA R:ATTGGAGCCTGGTGAAGACC	LG A	30.23	Not found	Not found
18	18049	F:ACCCCTCTTTGCTAGGGTGA R:ACCACACATCTCGCACACAT	LG B	65.61	Chr 1	2.3E-04
19	18358	F:CCTGAACCGATTTTGGTGAT R:ATTCCGCCCTCTTTCACTTC	LG B	100.89	Chr 4	1.7E-03
20	16512	F:TAAGCCCGACGCTTCTATTC R:GTGCCTCAGTTTCCGTTTGT	LG B	153.32	Chr 6	1.4E-04
21	18533	F:TCCAAAATGCGTGTCATCAT R:TGACCGACACATTCATCTTCA	LG B	177.69	Chr 4	4.3E-18
22	17754	F:AGCAACGGGCAACCTTATAG R:CCTTTTGTTTGGAAGCTCAA	LG C	0.00	Chr 2	9.5E-02
23	17531	F:TGCAGGGGTGTGTGTTACAT R:TGAACATGGTGAAATGGATTG	LG C	15.31	Not found	Not found
24	18237	F:GGGATATGAGAAGGCGATACC R:TGGTTGTAGGATGTGGGATTT	LG C	25.77	Chr 3	2.5E-07
25	18272	F:CCCCAACATTTCTCTAGGTAACA R:TTCTTCGCAGCTCGGTAAGT	LG C	57.91	Chr 1	4.7E-04
26	17713	F:AAAAAGGGGAAAGCAGGAGA R:TTGACTGTGAGGCTGGTTTG	LG C	157.15	Chr 2	4.1E-06
27	16570	F:CAAACACCAACCACCACAGT R:AAGGGGAGACGAAGTGGAGT	LG C	194.92	Chr 6	1.8E-03
28	16588	F:CGGTCTGAGGTTGTTGTGAA R:TTGTAAGACCGACTCGTCCA	LG E	0.00	Chr 2	1.6E-29
29	18928	F:TGAATGTGGAAAGGAGGAATG R:AGGGTCACCACTTTGGAGAG	LG G	14.69	Chr 5	4.6E-06
30	16452	F:CGATGGTTGCTGTTGTGAGA R:ACCCCAAACAAACACCAATG	LG G	29.64	Chr 5	5.6E-03
31	17605	F:CGCCCTTCATCATCATCTTC R:AGAGTCGGTCCCTCCAACAT	LG G	46.14	Chr 8	5.5E-04
32	17628	F:GGTTTTGTTTGCCGTTGATT R:CCACCCCCAAACTTCCTTAT	LG G	56.22	Chr 5	7.4E-28
33	18323	F:CAGACAATGGCAATTATTTGGTAA R:CTGCTGTTGCTTCGATTTCA	LG H	51.84	Chr 3	1.5E-10

## Discussion

SSR markers are excellent genetic markers because they are co-dominant, multi-allelic and reproducible. In genetics, SSRs have been widely used for diversity analysis [30], linkage map construction [31], QTL mapping [32] and association mapping [33].

Pea is important in genetics, because of the work of J.G. Mendel [34]. However, the pea genome is very large, which seriously hindered pea genomic research. The nuclear genome size of pea was estimated to be 9.09 pg DNA/2C, which corresponds to a haploid genome size (1C) of 4.45 Gbp [35], one and half times larger than the human genome of 3Gb [36]. Compared with other legume crops such as soybean (*Glycine max*) of 1.1 Gb [7] and barrel medic (*Medicago truncatula*) of 0.47 Gb (http://www.jcvi.org/medicago/), More efforts are needed to develop molecular tools especially for SSR and SNP (single nucleotide polymorphism) markers in order to build a solid foundation for its genomic research in peas.

### Using NGS technology for the identification of SSR markers is effective

Consistent with previous reports [37–39], results from this study demonstrated that Illumina paired-end sequencing offers an opportunity for high-throughput identification of SSRs with diverse motifs from economically important crop plant species. Within a relatively short time period, our sequencing experiment generated a total of 17.5 GB of raw paired-end sequencing data. From this raw data, 343,849 contigs were effectively assembled and used for SSR markers development. A total of 3,275 non-redundant primers were designed and nearly half of them (1,644 primers) were validated in two different ways, a diversity panel of 24 accessions and a segregating F2 population. For SSR markers development, NGS research strategy is very efficient.

### More reliable validation of the NGS based SSR markers was conducted

In the published studies of other plant species [40–41], only a small proportion of newly developed SSR markers was tested. In this study, more than half (1,644 markers) was carefully tested in two different ways. One way was genetic diversity analysis, the other way was the mapping of the novel markers to a linkage map based on an existing mapping population. More than 51% tested SSRs involved in this study were polymorphic among 24 accessions and clearly divided into 3 sub-groups. Meanwhile, 33 novel SSRs were anchored onto a previous genetic linkage group.

### Chinese pea germplasm differs from that of other countries

The comparison of the diversity of Chinese and foreign peas by using 841 polymorphic SSR markers in our study identified a significant degree of diversity (Figs 2 and 4). This result coincided with a previous study by using 21 informative SSRs to assess and compare the genetic diversity of 1,243 Chinese pea genotypes from 28 provinces to 774 pea genotypes that represented a globally diverse germplasm collection, and the Chinese pea germplasm was found genetically distinct from the global gene pool sourced outside China [28].

On the other hand, our genotype data did reveal an exception. In our experiment, G0002305 is an accession collected from Inner Mongolia. The cluster analysis grouped this accession into Cluster I with germplasm accessions collected outside China (Figs 2 and 4). Analysis of population structure also confirmed that this Chinese accession shared more than 80% of kinship with accessions collected from outside China, especially with G0006082 from Afghanistan and G0006170 from Pakistan (Fig 4). In addition, six Chinese accessions shared variable percentages (approximately 5 to 50%) of closeness with accessions collected outside China and two accessions collected outside China share a small percentage of closeness with the Chinese accessions (Fig 4). Both cluster and population structure analyses clearly separated the cultivated pea from its wild relative accessions (Figs 2 and 4). These results implies the usefulness of the newly developed SSRs.

### More SSR markers were anchored on a genetic linkage map

There was no genetic linkage map based on Chinese accessions previously. In 2014, we constructed the first Chinese pea linkage map constructed with 157 SSR markers [29]. In this study, the existing linkage map has been more saturated. The new map contained 199 markers including the 33 newly added markers. We anticipate that with more effective SSR markers, QTL mapping and association study as well as marker-assisted selection in pea will become available in the near future.

## Supporting Information

S1 File The code of reduce_ssr.py. (TXT)

S2 File Polymorphic SSR markers of *Pisum sativum* L. (DOCX)

S3 File Monomorphic SSR markers of *Pisum sativum* L. (DOCX)

S4 File Twenty two distorted segregation markers. (DOCX)
