# Supplementary material for: High-Throughput Development of SSR Markers from Pea (Pisum sativum L.) Based on Next Generation Sequencing of a Purified Chinese Commercial Variety
Source: PLoS One. 2015 Oct 6;10(10):e0139775. doi: 10.1371/journal.pone.0139775 (PMC4595016; doi:10.1371/journal.pone.0139775)
Supplement: S2 File — (DOCX) [file pone.0139775.s002.docx]

**S2: Supplementary Table 1 Polymorphic SSR markers of *Pisum sativum* L.**

| **No.** | **Marker Name** | **Primer sequences(5’-3’)** | **Repeat motif** | **AlleleNo** | **PIC** | **Ta(℃)** | **Real product Size（bp）** |
| --- | --- | --- | --- | --- | --- | --- | --- |
| 1 | 16144 | F:ATGTATGCTTTGGCCGGTAG R:AATTTTGTGGGCGCTATGAC | (GT)8(T)12* | 4 | 0.41 | 52 | 190-210 |
| 2 | 16145 | F:TGAAAAAGTTGTCATGCAAAGAA R:GAACCGCCTATGCCTGTAAG | (A)10g(A)10 | 2 | 0.11 | 52 | 110-125 |
| 3 | 16147 | F:GACAACAACAATAGTAGCTGGAGAA R:TTGTTCTTGTTTTTGTTTTTGTTATTG | (AAC)6(AAT)5 | 5 | 0.45 | 52 | 110-125 |
| 4 | 16148 | F:GTGAGTGTCGCGGTCCTTAT R:AGCATCAATCTTGCAATGACA | (TTA)5ctggtattactg(TTA)5 | 3 | 0.37 | 52 | 100-120 |
| 5 | 16150 | F:CGACTTTGGGCTCAAATCTAA R:TCAAGAACCGTTGACGCTAA | (A)10gtg(T)10 | 3 | 0.37 | 52 | 110-130 |
| 6 | 16151 | F:GGGGTGTGCGTCTCTATCTC R:AAGGCAACCCACACACAGAC | (TG)9cgcgcaaccatgc(GT)7 | 3 | 0.28 | 52 | 110-130 |
| 7 | 16152 | F:ATGCACACCATGTTGCAGTC R:ACATCAACGCCAGGAAGTTT | (TG)6a(TGG)5 | 3 | 0.08 | 52 | 110-130 |
| 8 | 16153 | F:CGTGTGTGTGTGTGCTTGAG R:ATGCAAACTCACAGGCAGAT | (TG)7tttatgtgtc(TG)10 | 3 | 0.27 | 52 | 110-150 |
| 9 | 16154 | F:GGAGATTTCGCTGGAGTCAA R:CCTCCTCAAGATCCCTCACTC | (CTT)5tcgatc(CTT)5 | 4 | 0.34 | 52 | 120-140 |
| 10 | 16157 | F:CCCTCATCACTACCAGCTTCA R:CAGAGCACTAGCTGGCCTGT | (CAT)6(CAG)5 | 6 | 0.42 | 52 | 90-120 |
| 11 | 16159 | F:CGTTAACTCCCACCACCATA R:GTTTCGGTCGTGGCTTACAT | (GA)7gtattcatttgatgctttcg(TC)7 | 3 | 0.32 | 52 | 120-140 |
| 12 | 16162 | F:TCCTCCATTTTTGGTGTGCT R:CTAAACCGGGGCCAAAAATA | (TG)6t(AC)6 | 2 | 0.21 | 52 | 120-140 |
| 13 | 16168 | F:AACGCGCCTCTTTTCATTAG R:AGAGAACCAATAACACACTTAAACTCT | (T)12ac(T)11 | 3 | 0.44 | 52 | 120-145 |
| 14 | 16170 | F:TTCTACCGGTTTGGTTTGGTA R:GAGGCTTAAAAGGGCGAGAT | (CA)8t(AC)7 | 4 | 0.53 | 52 | 120-150 |
| 15 | 16172 | F:TCGGTTGTCGTTTCTTGTTG R:TCATCCATAAACCCCCTCTC | (GA)6tagtttaagcgagaatggc(GA)8 | 6 | 0.75 | 52 | 150-170 |
| 16 | 16180 | F:TGTAGTTATTGTTGTTGTGGTTGAAA R:CGTCTCCCGTATCAGCAAGT | (TTG)5aggttgttgttggtaaca(TTG)5aattggtattatgaaagt(TTG)5 | 4 | 0.59 | 52 | 110-130 |
| 17 | 16183 | F:GACGCTCTGGCATTCTCTGT R:TGTGCACCATTGGAGGAATA | (GTT)5gtggttgtcctc(GTT)6 | 2 | 0.14 | 52 | 150-165 |
| 18 | 16185 | F:TGATCATGCTCCATAGGTTGA R:TTGGGATCCTACAACACGAATA | (TCA)5(TCT)5 | 4 | 0.37 | 52 | 140-160 |
| 19 | 16191 | F:GGGTTGTCGATAGAGGGTGA R:AAAACAAAACCTTTTTCGTCTTTC | (TG)10(AG)10 | 3 | 0.4 | 52 | 165-185 |
| 20 | 16194 | F:GTTCTGGCACGACAAAAATG R:CACATACAAACACGCACACAC | (TG)7tatgtata(TG)8 | 6 | 0.48 | 52 | 160-180 |
| 21 | 16195 | F:CGTGCATGCGTGTGTATGTA R:ATACCCACACGCCAACTCTC | (GT)8atgcttgg(GT)8atgtgtatgtgtatgtgta(TG)7 | 3 | 0.29 | 52 | 120-130 |
| 22 | 16196 | F:ATGCCAAAAGGCAGAAAAGA R:TCAGAACTCCCAAAACCCTTA | (CA)7cttagataaaaag(CA)6 | 4 | 0.42 | 52 | 90-100 |
| 23 | 16199 | F:GAAGCAACACAGCAGCTCAC R:AATGCGATGCAATGCAATAA | (CT)9tagggtttct(TTC)6 | 4 | 0.66 | 52 | 170-185 |
| 24 | 16201 | F:TTGCATGGTCAACCATTTTT R:GGGGTTGAGCTTGGTTGTT | (TC)6tg(TC)7 | 3 | 0.42 | 52 | 170-185 |
| 25 | 16204 | F:ACCGCTGAATCTGACGTACC R:AGTGAAAACCGAGGTGCAGT | (TC)7tggtcca(TC)7 | 4 | 0.59 | 52 | 180-190 |
| 26 | 16205 | F:ACACTTGGGATGGCTCTACG R:TGCTCCATTGGTTGAGAGTG | (GAT)7a(ATG)9 | 3 | 0.48 | 52 | 170-190 |
| 27 | 16206 | F:TTTGAATTCCCCAGAACCTG R:CCGGAGGACGTACCTGTAAA | (AC)11at(AC)9 | 3 | 0.36 | 52 | 160-185 |
| 28 | 16208 | F:GGGTATTTGAGGGGAGAGTCA R:CACGTGCCCTTTTGAGTTTT | (CT)9t(TC)6 | 7 | 0.68 | 52 | 180-210 |
| 29 | 16211 | F:TGCCCTAGGCCCTACCTTAT R:GGTTGCGGTGTTGGATATTG | (CAT)6tatccatcatggag(AAC)5 | 2 | 0.11 | 52 | 180-190 |
| 30 | 16213 | F:GATCGCCACCTTCCATTCTA R:GCTTGAGGGGAAACTTTGAA | (CT)11caaactcattcacct(TC)7 | 3 | 0.39 | 52 | 175-190 |
| 31 | 16214 | F:TGTGCGTATGTGTGTGTGGT R:TTTGCATCAAAACCGCATAA | (GT)6gcgtgtgtgggcgcgtgc(GT)7 | 2 | 0.14 | 52 | 180-200 |
| 32 | 16216 | F:AGCACAAGGCATCGAGATTT R:GAGGCACATGTTGCTTTTGA | (AGA)5at(AAG)13 | 6 | 0.72 | 52 | 150-190 |
| 33 | 16223 | F:AAGCATAAGGGCTTTCTCATAA R:CCGGGATAACCACGAACTAA | (AC)7atgcatg(CA)6 | 4 | 0.49 | 52 | 195-215 |
| 34 | 16224 | F:TCGAACCTTTGAACCCATGT R:TGATATGGGTGCCAGGAAAT | (AC)14(AT)6 | 3 | 0.43 | 52 | 140-160 |
| 35 | 16225 | F:GTGCTTCGGTGGAAAATCAC R:CTCAACCGCGAGGTAGGTAG | (TC)12c(CT)6 | 3 | 0.12 | 52 | 170-210 |
| 36 | 16226 | F:GCGTGCATACGTGTGAGTTT R:ACACGCACATGCAAATGCTA | (GT)8acgtgcatgtgtttgcatgc(GT)6gcgctagcatttgc(GT)8 | 4 | 0.5 | 52 | 170-210 |
| 37 | 16227 | F:GCAGTCACAGAATCTCGTTCA R:TCCAGCACCCACATAGTCAA | (CT)10t(TC)7 | 5 | 0.55 | 52 | 190-210 |
| 38 | 16228 | F:CTCACCACACGCACTTCCTA R:CGTGACCGGAGATTCCACTA | (AAG)8cattgag(AGA)6 | 3 | 0.19 | 52 | 190-210 |
| 39 | 16234 | F:GAGGAAGCGTAAAAGCTCTCAA R:TGTGTGTGTCTGTTTCTGTGTG | (AC)9atat(AC)13 | 2 | 0.31 | 52 | 190-210 |
| 40 | 16237 | F:GCAAACGAAGCAGGCTTATC R:TTGGCTGATCCTGAAACTGA | (CATTTC)5 | 3 | 0.53 | 52 | 150-170 |
| 41 | 16238 | F:GTTGGAGGCGAATCTAGTGG R:CACGCTTCTTCGTCAAAACA | (GATGTT)5 | 3 | 0.48 | 52 | 150-175 |
| 42 | 16239 | F:CTAAGAGCCCAACACCAACA R:GTGGGGATAAGGGGAGAGAG | (ACACCG)5 | 5 | 0.68 | 52 | 180-210 |
| 43 | 16240 | F:TGATGGGTCTGCCTCTTCTT R:TCACAAGCTCTTTGTTTCTTTTTC | (AAAAAG)5 | 2 | 0.31 | 52 | 185-200 |
| 44 | 16241 | F:TATCTTTGGTCGCGAACCTC R:ATACGGCGAAGGAGGGTAGT | (AATCCC)5 | 3 | 0.58 | 52 | 185-210 |
| 45 | 16242 | F:GGGAAGTGTTTTGGTCAATCA R:CGGCCACTTCCTATTCTTCA | (GAAAC)5 | 3 | 0.48 | 52 | 100-120 |
| 46 | 16243 | F:GAGATCCAGCGCCAACTTAG R:CACCATGTAAACTCTAAACATGCATAC | (TATGT)5 | 3 | 0.5 | 52 | 120-130 |
| 47 | 16244 | F:TTTTCGATTTTCGCATCCTC R:ATGGTTTCAGCTACGGTGGT | (ACAAC)5 | 3 | 0.45 | 52 | 180-200 |
| 48 | 16245 | F:TCGATGTTTGACCCGATTTT R:AGGTCCTTTGGAGCCATTTC | (AAGAG)5 | 3 | 0.34 | 52 | 190-210 |
| 49 | 16246 | F:CCTACCCCACAAACTCTGTAGC R:TCTCGGTTGCTTCCTGATTC | (CTTT)5 | 3 | 0.43 | 52 | 110-120 |
| 50 | 16248 | F:CCCTGCACAAGAAAATGTCA R:GGTGATGATCCATGGCAGTA | (TATG)5 | 4 | 0.59 | 52 | 120-130 |
| 51 | 16249 | F:AAGCACAATGATCTGTAAGGAAAA R:TTTGGAGATGGAGAGCACTG | (CTAA)5 | 3 | 0.35 | 52 | 100-130 |
| 52 | 16250 | F:GGAATGGGGTGGGTAGTTTT R:CGATTTTGCAGACGCATTTA | (TTAT)5 | 3 | 0.18 | 52 | 110-130 |
| 53 | 16251 | F:GCTGAAAGTCAAAACGTGGA R:GTTTCTGGCCAAACATGGTC | (ATGC)5 | 3 | 0.4 | 52 | 125-140 |
| 54 | 16252 | F:TCAAGAAATTTCACGTACTCATTCA R:AAGCCAGCTCCTAGCCATTA | (TTCA)6 | 2 | 0.37 | 52 | 130-140 |
| 55 | 16253 | F:GGAACTTGGCGAGTGTGAAT R:TTCATGTGCCCGTTAATCAG | (ATAG)5 | 5 | 0.65 | 52 | 125-140 |
| 56 | 16255 | F:TTCAAACCGCCTACATGAGA R:AGCACCGTCGACTTACTGGT | (AATA)5 | 4 | 0.43 | 52 | 160-180 |
| 57 | 16257 | F:GACATCCCCTTCCAACAAAG R:TCTTTCCACTGGCTCATTTTG | (TCAA)5 | 2 | 0.14 | 52 | 160-170 |
| 58 | 16258 | F:TCCAAATCCTCCCTTTACACA R:GGTCTTTTGCATGCTGATTG | (AATC)5 | 2 | 0.08 | 52 | 160-180 |
| 59 | 16259 | F:TCACGAGCAGCTCCTTTTCT R:GAAACCGTAACCGATGCAGT | (CTAA)5 | 4 | 0.48 | 52 | 190-200 |
| 60 | 16260 | F:CAAATTGCTGTAGAAGCCAATG R:CGCGCAGCTCAGTAGAGA | (AACC)5 | 4 | 0.36 | 52 | 190-210 |
| 61 | 16262 | F:TATGGAAGGGGAATGCAAAA R:CTTAACAGCCCTGGGAATCA | (TTAA)5 | 2 | 0.33 | 52 | 190-200 |
| 62 | 16265 | F:GGCTACCTCGGTTGTTGAGT R:TCCCTCAAAGACCTTGTCGT | (TGG)5 | 7 | 0.38 | 52 | 100-120 |
| 63 | 16267 | F:TGACTAACACCACCGCATGT R:GGCAGTTGTTGCACATCTCA | (TGA)5 | 2 | 0.26 | 52 | 110-120 |
| 64 | 16268 | F:CAATGGCTTCACTCATCACC R:TGTTGTCGTTAAGGATCTTCTCA | (TCA)5 | 3 | 0.23 | 52 | 110-120 |
| 65 | 16270 | F:GTTGGGGTACGAGGAGAGTG R:CACTCACAGCACAATGTCTCAA | (GAA)5 | 3 | 0.15 | 52 | 110-120 |
| 66 | 16273 | F:TGTGCCAATTGTTGATGAAG R:GGTTGAAGACGATCCCAATTT | (GAT)7 | 2 | 0.08 | 52 | 110-120 |
| 67 | 16274 | F:TGCCACCGGGTAGTCATTAT R:GTTGAGGTGGAGCATGTTGA | (CTT)6 | 2 | 0.14 | 52 | 100-120 |
| 68 | 16275 | F:CATTTTCGTTTCTCCGTCAAA R:CGCCAGGAAGAGTAGGAGTG | (CTT)5 | 4 | 0.52 | 52 | 100-125 |
| 69 | 16276 | F:TCAATGTTATCCACCAAGGAAAG R:GCTCTTGCGCATCTCACATA | (CAA)7 | 2 | 0.08 | 52 | 110-125 |
| 70 | 16278 | F:GTGAATCCAGCGGTTTGAAG R:TGGAGGGAGTTTGGAGTAGC | (ATG)7 | 2 | 0.14 | 52 | 90-110 |
| 71 | 16279 | F:TCCCCTCTCTAAAATCGTGAC R:TTTACAATCAGGGTGGGTTTG | (ACA)5 | 2 | 0.37 | 52 | 110-120 |
| 72 | 16281 | F:AACTTCCTCCGCCAAATCTT R:TGTGCGTTAAATGAATAAAAATGAA | (TCT)5 | 5 | 0.55 | 52 | 110-125 |
| 73 | 16283 | F:CGAGATTGAGGGAATGTCGT R:TAAGACTCGGGCGTCAACTT | (TAA)5 | 4 | 0.31 | 52 | 110-120 |
| 74 | 16288 | F:CAGCCAAAATAGGGGTTTGA R:CCAGGGTCGACTTTTATCTGA | (ATT)5 | 2 | 0.08 | 52 | 110-130 |
| 75 | 16291 | F:TAGTCAGCAACACGCTCCTC R:GCCCTGGGAGTAAGGAAGTC | (TTC)5 | 3 | 0.15 | 52 | 110-125 |
| 76 | 16292 | F:TTCAACCTTCTTTTTCACTTGAC R:GGTTTTATGGGATCCGTGTG | (TTC)5 | 2 | 0.34 | 52 | 110-125 |
| 77 | 16293 | F:CCCCATGTTGAGTTGTTGTC R:TTATTCAGACGCTGCACCAC | (TGT)5 | 2 | 0.37 | 52 | 110-125 |
| 78 | 16294 | F:GCCAACTGTGAGTGAGCTGT R:GCCGAAAATGTAGGGATTCA | (CCT)6 | 6 | 0.56 | 52 | 100-130 |
| 79 | 16295 | F:TTCCTACCGATCAACGAACC R:AGGCCTTCTCTGCCTTTGTT | (CAA)5 | 4 | 0.57 | 52 | 110-130 |
| 80 | 16296 | F:CTGCTTCTCCAGCTGCTTGT R:TCTGAGGAAACAACGTGAGAAA | (ATC)6 | 3 | 0.32 | 52 | 110-125 |
| 81 | 16297 | F:TGTTGTGGTTGCTGTTGTTG R:TCAACCACCCCGTTAACATC | (TTG)5 | 3 | 0.25 | 52 | 110-120 |
| 82 | 16298 | F:TGAATGTACGACATTGGTACTGG R:CCACAAACGCTATCGTCAGA | (TTG)5 | 3 | 0.15 | 52 | 110-120 |
| 83 | 16301 | F:GAGGCACAAGTTGCTTCTGA R:TCGCAAATCAAACCCCTAAC | (TCT)5 | 3 | 0.43 | 52 | 110-125 |
| 84 | 16302 | F:ATAACAACGGGGAGGAGGAG R:ACCTCTCCGACCAACAACTG | (GGT)6 | 2 | 0.19 | 52 | 100-120 |
| 85 | 16303 | F:GGGTGGGTGTGATGAAATCT R:TTGATATGACTGGCGGTGAA | (GGT)5 | 2 | 0.28 | 52 | 100-120 |
| 86 | 16306 | F:CCCATCATCAGCATCATCAA R:GGTAATGTTCCTGCGACTGTT | (CTT)6 | 3 | 0.26 | 52 | 100-120 |
| 87 | 16309 | F:TGAGTGTTGAGCCGAATCTG R:CACTTCACCACACTCTCTCTCCT | (TGA)5 | 3 | 0.23 | 52 | 110-125 |
| 88 | 16310 | F:ATTCGTGGGAGCCCTTTATT R:TCAACATTATCCTTCTATAACATCTTG | (GTT)5 | 3 | 0.48 | 52 | 110-125 |
| 89 | 16311 | F:TCATCACGATGAACGATAACC R:CCAGGGGAAAAAGAATGTGA | (CTT)6 | 2 | 0.14 | 52 | 110-125 |
| 90 | 16312 | F:ACCGGTAGGATTTCCCAAAC R:TCATTGGAACTCGACGCTTA | (CCA)6 | 3 | 0.35 | 52 | 110-125 |
| 91 | 16318 | F:GGCCATCTCTTCCCATCAG R:ACCACTTCACGCTCCTCCTA | (GAG)5 | 3 | 0.15 | 52 | 110-125 |
| 92 | 16320 | F:CTCATGGAAGTCAAAGAAACAA R:TGGTGGTGATGCTTTATTGAA | (CAA)6 | 3 | 0.23 | 52 | 110-125 |
| 93 | 16322 | F:TGTCGTGGCCATTCTAGTCA R:TCCTAGGTTGTCTTGGCTGATT | (CAA)5 | 4 | 0.58 | 52 | 100-125 |
| 94 | 16323 | F:GCGGTTTTATCTCGTTGCATA R:TCAAACCGAAGAAGCGAAGT | (ATT)5 | 3 | 0.21 | 52 | 100-120 |
| 95 | 16324 | F:AGACCTTCGTGTTCATGTGCT R:GGCCATAGCTCGTTCATCAT | (ATG)5 | 2 | 0.24 | 52 | 100-120 |
| 96 | 16325 | F:TGCACGACAAGAGGATGAAG R:CCTCAGGTGGTTGAGACTCC | (AGG)6 | 2 | 0.08 | 52 | 100-120 |
| 97 | 16327 | F:CCTCACCTCACCGTTTCCTA R:ACCGGAGAAGACGATCAGAA | (AGA)7 | 3 | 0.46 | 52 | 110-125 |
| 98 | 16328 | F:GAAGCTTCGATCTTGCTTGAG R:GACTTTGCCTTGCACGTTTT | (AGA)6 | 2 | 0.08 | 52 | 110-130 |
| 99 | 16329 | F:CAAAAGTTACCGTTTGTTTCCATA R:ACGAAGCATTGGATCACACA | (TTG)5 | 3 | 0.58 | 52 | 110-130 |
| 100 | 16333 | F:GGATCGGTTCTCAACCTCAA R:CTTCGCTTCTGCTCGAATCT | (GAA)5 | 3 | 0.21 | 52 | 100-120 |
| 101 | 16334 | F:TGTGGTCTGAATCAGGGTTTC R:GATCAATCACTTGATTCTCCTGAA | (CAT)5 | 2 | 0.35 | 52 | 100-120 |
| 102 | 16336 | F:TACGTAGGAGCCGGATTTTG R:TGTGAATTGTTTGCCATCTTCT | (ATA)5 | 3 | 0.5 | 52 | 110-130 |
| 103 | 16337 | F:CTGTCAGCATCCCTCACAAA R:AGCACGCATACAGGGAAGAG | (AAT)5 | 2 | 0.08 | 52 | 110-125 |
| 104 | 16338 | F:TCAGCCACCAAACTTCTTGA R:AAGGAGCTCAGAGGCACAAG | (AAG)6 | 2 | 0.08 | 52 | 110-125 |
| 105 | 16340 | F:TTCGCCGAAGTTAGGTAAGC R:CCTCCTCAAGATCCCATTCA | (TTC)6 | 10 | 0.82 | 52 | 110-130 |
| 106 | 16341 | F:AATCACCTCGGGTGAGTGAG R:TCGAGCTTTAACCTTCCACTG | (TTC)5 | 4 | 0.5 | 52 | 110-130 |
| 107 | 16349 | F:TGAAACTGACGATGATGAACAA R:ATGGCTCTTGCACGTGGTTA | (GAA)5 | 3 | 0.15 | 52 | 110-125 |
| 108 | 16350 | F:CACCTCCACCCTTTCACCT R:CTGGAGGTGGGAGATTGTCT | (CTC)6 | 2 | 0.11 | 52 | 110-125 |
| 109 | 16351 | F:CACATTCATCCAGAGAATACAAAAA R:TGAGGATGGTTGTGCTTCAA | (CAT)5 | 2 | 0.37 | 52 | 110-125 |
| 110 | 16352 | F:AGGTGAAAACGGAAGACCTG R:CTTCAGCTGCCATGTTGTGT | (CAT)5 | 3 | 0.24 | 52 | 110-125 |
| 111 | 16353 | F:CAATGCAACAGCAAGCAAAT R:AAAAGCCCCAAATCCTGAAT | (CAG)5 | 2 | 0.37 | 52 | 110-125 |
| 112 | 16356 | F:GTGCTGGTGTTGTTGGTGAC R:CCATCTTCGATTTGGCTCAG | (TTG)5 | 2 | 0.08 | 52 | 110-125 |
| 113 | 16357 | F:AGTGAGGAGCGCATTTTTGT R:GTTTGGGAAATGGGAAGGAT | (TTC)7 | 3 | 0.52 | 52 | 110-130 |
| 114 | 16358 | F:CGGAGAGGACGATCAAAAAC R:GCCACTGCATTCTCATCTCA | (TTC)5 | 4 | 0.49 | 52 | 110-130 |
| 115 | 16359 | F:AAGTCTCACCGAAGCACACA R:TTTGCAACGCACAGAAGAAG | (TTC)5 | 3 | 0.38 | 52 | 110-125 |
| 116 | 16364 | F:CCGCCGGTAGTAGTGAAAGA R:TGGACTCTGCTGCACATCAT | (CAG)5 | 3 | 0.21 | 52 | 110-130 |
| 117 | 16365 | F:TCCGTTTGCTTCCTTTTCAC R:CATAGCGGGAATTTTGAGGA | (CAC)7 | 4 | 0.43 | 52 | 110-130 |
| 118 | 16366 | F:TTGGGGGTTTGATGGTTTTA R:ATGAACTCCGGTCCAGTAGC | (ACC)6 | 4 | 0.28 | 52 | 110-130 |
| 119 | 16368 | F:AGAGGCATTTGGTGCTTCAC R:GGGCAAAACATAGAAAAGGTG | (TTA)5 | 3 | 0.18 | 52 | 110-130 |
| 120 | 16373 | F:CAGGGCTGACAGTGATGAAA R:AGGACTGTCGTCTCCACCAT | (GAA)5 | 3 | 0.43 | 52 | 100-130 |
| 121 | 16375 | F:GAAATGCTCCCATTGCATGT R:CAACACTGACATCTCCACACC | (CAC)5 | 3 | 0.15 | 52 | 120-140 |
| 122 | 16377 | F:GCACCCAAGATGGTGAAATC R:GGAGGAGCATGGTGATGAAT | (ATC)5 | 4 | 0.28 | 52 | 110-140 |
| 123 | 16378 | F:CAACTGCGCTCAGAAGAATG R:TGAAGTGGTGGCAAGAAACA | (AAG)6 | 2 | 0.08 | 52 | 120-140 |
| 124 | 16381 | F:TGGAATGATGTTGGTACCTTTG R:GCGGTTGTTGACGACACTTA | (TTC)7 | 2 | 0.3 | 52 | 110-125 |
| 125 | 16383 | F:AATCCCTTGACGTTTTCACC R:AAAGAGATGAACTTGGGGAATG | (CTT)6 | 3 | 0.15 | 52 | 120-140 |
| 126 | 16386 | F:CGTCAATAACAACCGCAGAA R:GGGCAGAGTTAGGCGTATGA | (ACA)5 | 2 | 0.37 | 52 | 110-120 |
| 127 | 16390 | F:ATCCTCCTCCTCGAGGTCAT R:AATGCTGCTCTGGGAGGTAA | (TTC)5 | 5 | 0.64 | 52 | 120-130 |
| 128 | 16392 | F:TTGGACTGGTGTGATGGAGA R:AAATCGTGCAGCAACATGAG | (GTT)6 | 3 | 0.24 | 52 | 120-130 |
| 129 | 16393 | F:ACGAGCAAATTGAAGGGAAC R:TTTCTTCTCCTGGCACATCA | (GAA)5 | 2 | 0.14 | 52 | 120-130 |
| 130 | 16395 | F:TGAACGACAAGCACAACTCC R:GCGGTCAGAGTTGCTGATAA | (CAA)6 | 4 | 0.28 | 52 | 120-140 |
| 131 | 16396 | F:CCATGGTTCCTCCACCATAC R:TAAGGCTTGGTGGTGGTTGT | (CAA)5 | 3 | 0.15 | 52 | 120-130 |
| 132 | 16397 | F:AGGGCCAGGTTTATTTCCAC R:TTTCCCAATGGCAAGTTAGC | (ATC)8 | 4 | 0.41 | 52 | 120-140 |
| 133 | 16401 | F:ACCCCACCATCTCTTTTGTG R:TCAGGGTGGTAGGAGGATGA | (TCA)5 | 4 | 0.46 | 52 | 120-140 |
| 134 | 16402 | F:ACTCCGTTCGGTCATTTCTT R:TCTTCCCTGTTGCTGCTTTT | (GCA)5 | 2 | 0.36 | 52 | 120-140 |
| 135 | 16403 | F:CTTGTTGGCCACCTTTGTTT R:TGGTGCAGTTGTCGTAATTTG | (CTT)6 | 2 | 0.17 | 52 | 120-140 |
| 136 | 16404 | F:ACAACACGAAGCGTGAACAG R:CGCTTTTTGCTTCTCACACA | (CAA)5 | 3 | 0.15 | 52 | 120-140 |
| 137 | 16405 | F:GACCGGAGCCACAAAAATAG R:GGAAAGGGTTCTGCCATGAT | (AGG)5 | 3 | 0.42 | 52 | 140-150 |
| 138 | 16406 | F:TTCAGAAGTTGCAGCACGAT R:TTGCATAACGAGAGGCCATA | (AGA)6 | 2 | 0.34 | 52 | 120-140 |
| 139 | 16407 | F:GTTTCACTCACGCGTTTCAA R:AGAAAAAGGGGAGCAACTCA | (AGA)5 | 2 | 0.14 | 52 | 120-140 |
| 140 | 16408 | F:CACGCAAAAACACAAACACC R:GTTGCCGGTTTTGAGAAGAG | (ACA)5 | 3 | 0.15 | 52 | 120-150 |
| 141 | 16409 | F:TTGGACTGGCTCCCAGATTA R:AAAGGCATTGGTTGCTTCTG | (AAG)5 | 3 | 0.44 | 52 | 120-150 |
| 142 | 16410 | F:AAGGTCATGCTTCTTCATCTCT R:GGGTGAGGTGTTATGGCACT | (TTG)6 | 3 | 0.43 | 52 | 120-140 |
| 143 | 16412 | F:ATGCTTCTCCGGGAGGTAAT R:ATCCTCCTCCTCGAGGTCAT | (GAA)5 | 4 | 0.52 | 52 | 120-140 |
| 144 | 16414 | F:TGCTGTCGTAGTGCCTAGTGA R:TTCTGAAAACCCGAGTAAAATCA | (ATT)5 | 2 | 0.29 | 52 | 125-140 |
| 145 | 16415 | F:TTCAGAGGTATGCTTGTGAGAAA R:TCCAAGGGTTTAGGGATTCA | (ATG)5 | 3 | 0.42 | 52 | 120-130 |
| 146 | 16422 | F:TTGGCAAGCCTATTGTTGTG R:CCCTCTGTGAAGAGGAAGGAC | (TGT)5 | 2 | 0.14 | 52 | 120-140 |
| 147 | 16423 | F:GCACATGCTGCTTCTGAGTC R:GCCTGGACTGGCTCCTAGAT | (TCT)7 | 2 | 0.35 | 52 | 120-140 |
| 148 | 16424 | F:ACAAAAGCGGGAAACGACTA R:TTGGATCTCACATGCCTCAT | (TCA)10 | 8 | 0.57 | 52 | 100-130 |
| 149 | 16431 | F:GGGGGAGTGGTTGAGAAAAT R:CAAAATGCCATCAGCATCAC | (GTT)7 | 3 | 0.08 | 52 | 120-150 |
| 150 | 16433 | F:CACCGCAAACATAGCAAAAA R:TCTCATAGCTGCGAGGTTCA | (GAA)6 | 3 | 0.4 | 52 | 125-135 |
| 151 | 16434 | F:CTTCTGGCCATCCAAGTCAT R:GTTGTTGGCTTATGCATCTGT | (CTT)5 | 6 | 0.69 | 52 | 120-140 |
| 152 | 16435 | F:AAAACGAGAAGGCAAGACCA R:TGGTTGTTGAACGGGAATAA | (CAA)5 | 2 | 0.34 | 52 | 120-140 |
| 153 | 16436 | F:GAATTAGCGGTCTTGGGTGA R:CCTTCTCAAATCCCCAATCA | (ATA)5 | 2 | 0.24 | 52 | 125-140 |
| 154 | 16437 | F:TTGTTTTTGTTGTTCTTGTTGTTG R:TTTTCGGGTTTTGCTTATGG | (TTG)7 | 4 | 0.58 | 52 | 160-180 |
| 155 | 16438 | F:AAACGGTATGCTTCCACCTG R:CCTGCAAAAGCTTCAATCCT | (TTC)5 | 2 | 0.08 | 52 | 125-140 |
| 156 | 16443 | F:AGCAAATGAGCCACGGTAAG R:TTTGCAATCAACCGAGAGAA | (GAT)5 | 3 | 0.34 | 52 | 120-130 |
| 157 | 16444 | F:TCATCACTCTCCTCTCACGTGTAT R:TCGAATCCGAAGCTATCACA | (GAA)5 | 2 | 0.08 | 52 | 120-130 |
| 158 | 16445 | F:TCAAACCGCTGAAAAACAAA R:GCGGTGGGAGGGAGATAC | (CCA)7 | 3 | 0.45 | 52 | 120-140 |
| 159 | 16446 | F:CAAAATCACATTCGCTCATCA R:GCCTTCCATGGTAGCCATAC | (CAT)5 | 3 | 0.44 | 52 | 125-140 |
| 160 | 16447 | F:CACTGCAAGGAATGCAACAA R:CTGCCTGATTTGGCTAGGAG | (CAG)5 | 2 | 0.37 | 52 | 125-140 |
| 161 | 16448 | F:TGGGGACTAAGAGCACTTGAA R:CGGAGGTCTGTTTCTGCACT | (AGA)6 | 4 | 0.51 | 52 | 110-140 |
| 162 | 16449 | F:TCACCTCAGAACCACGACAC R:GCCGACGTCAGGTAAGATTT | (AGA)10 | 4 | 0.57 | 52 | 120-140 |
| 163 | 16450 | F:TTGCAAAATCGGGAAAACAC R:TCCACCACCACCTTTACCAT | (AAC)6 | 5 | 0.43 | 52 | 120-140 |
| 164 | 16452 | F:CGATGGTTGCTGTTGTGAGA R:ACCCCAAACAAACACCAATG | (TGG)6 | 4 | 0.64 | 52 | 125-140 |
| 165 | 16453 | F:CGGTGAAAACGAAATGGAGT R:TCAATTCATGCATCCGAAGA | (CTT)8 | 5 | 0.64 | 52 | 120-140 |
| 166 | 16454 | F:ACGTCACAGCCACAACACAT R:AAAGATGGGACGAACGTCTG | (AGA)11 | 2 | 0.37 | 52 | 125-140 |
| 167 | 16456 | F:GAAGTGAATAAAGATGAGCGTGAA R:TCACCTTAAAGAACCGGAAAA | (AAG)6 | 2 | 0.19 | 52 | 125-140 |
| 168 | 16459 | F:TACACCCATGGCTGAACCTT R:GGGGTCCTGTTGATGAGAAA | (GCC)6 | 3 | 0.26 | 52 | 120-140 |
| 169 | 16460 | F:CATTGAAGCTTCGCATTGAA R:TTGATCTTGTTTTGCACTCTCC | (GAA)5 | 7 | 0.7 | 52 | 120-150 |
| 170 | 16465 | F:TGGTGAGTTTCCAAAGGATGT R:CATCAGCCATGGCATACACT | (TGA)5 | 2 | 0.08 | 52 | 130-140 |
| 171 | 16467 | F:TTCTTCATGGATGGCAACAA R:GGGGTGGATGAAGATGAAGA | (CCA)5 | 4 | 0.25 | 52 | 130-140 |
| 172 | 16468 | F:TCCTTTTCCACGTGCTCTTT R:CACGCCCTGGTCATACTCTC | (CAT)5 | 3 | 0.37 | 52 | 130-140 |
| 173 | 16469 | F:CTCCTCCTTCTCCCACATTG R:TGGTTGGTTTCCATGAGGAC | (CAA)5 | 3 | 0.21 | 52 | 125-140 |
| 174 | 16470 | F:TCTGCTTGAGCTAAGCCACT R:GGTCGGAAACTTCAGCAGTC | (AGA)6 | 4 | 0.31 | 52 | 110-135 |
| 175 | 16471 | F:TTACCGCGATGACAGAAGAA R:GCGTAACAGAATAACTCGGTACA | (TTC)5 | 3 | 0.38 | 52 | 125-140 |
| 176 | 16474 | F:TATCGCGACTTGGTCATCTG R:TCCATCAGACGATGGTAACG | (GAG)5 | 2 | 0.35 | 52 | 125-140 |
| 177 | 16476 | F:CACCCTCTCTGCAAATGTTCT R:TCTGGAATCCCTTCCAGTTG | (CAA)5 | 3 | 0.12 | 52 | 130-150 |
| 178 | 16482 | F:ACCCTCGGATCAGCATAGGT R:GCGAACAACTTTCCCAACAA | (GTG)6 | 2 | 0.35 | 52 | 130-150 |
| 179 | 16485 | F:CGAATTCCTTGCTTTGCACT R:CTTCAGAACATGTCCCAACATC | (TCT)5 | 3 | 0.15 | 52 | 125-140 |
| 180 | 16487 | F:GAACATCTTCTGGAGTTGAAGGAT R:TCTTGTTTCACCGCCTTCAT | (GTA)5 | 2 | 0.08 | 52 | 130-140 |
| 181 | 16488 | F:GGACGTGGTGAACCTAGTGG R:TACCTTGCCGATCAATCTCC | (GAT)6 | 3 | 0.33 | 52 | 130-140 |
| 182 | 16491 | F:GATGGGAGTGGAGGAACTGA R:CTCGATTCGCATGAAACAGA | (AGA)6 | 4 | 0.21 | 52 | 125-140 |
| 183 | 16493 | F:GCATAGCGGTTCACACACAC R:TCAATCGTTGCATGCTGAAT | (AAC)5 | 3 | 0.24 | 52 | 130-150 |
| 184 | 16497 | F:CCGGTCATCACAACTCTTCA R:CGGGAGCGGATCAATTACT | (CAA)6 | 3 | 0.43 | 52 | 125-140 |
| 185 | 16499 | F:ATGTCGCCAAGGAGTGAAAA R:TGGAACACTTGAGAGAGGATGA | (AAC)5 | 2 | 0.08 | 52 | 130-150 |
| 186 | 16501 | F:ACGGTCGTTGTTGTTGAGGT R:GTAGCCGCAGTGGATCTCAT | (TGT)5 | 2 | 0.37 | 52 | 125-150 |
| 187 | 16502 | F:TGAGACGAGTTGTTCGAGTGA R:TCCTCATTTTGACCGCAATC | (TGT)5 | 3 | 0.4 | 52 | 125-150 |
| 188 | 16504 | F:TACGGAGGTGGAAGCAATTC R:TGAACCAGCTGAGGATGATG | (TCA)5 | 3 | 0.41 | 52 | 125-150 |
| 189 | 16505 | F:AATGTTGGGGTAACTGATGTTG R:TCCCGAATGAAACACATTCA | (GTG)6 | 3 | 0.15 | 52 | 130-150 |
| 190 | 16507 | F:GGAGAATCGCAACTATGATTTGA R:CGAGTTCTCGCAATGGATTA | (GAA)7 | 3 | 0.18 | 52 | 130-150 |
| 191 | 16508 | F:CCTCAATCCACATCCCATTC R:GAACCCCCATTAGGGAACTC | (CTT)6 | 2 | 0.37 | 52 | 130-150 |
| 192 | 16509 | F:GGCCGCTTCGGTTAGAAT R:GGAGAAAAATGGAAATTCCTCTG | (ATC)5 | 3 | 0.15 | 52 | 130-150 |
| 193 | 16510 | F:TTCAAGCCGAAACTGATGAA R:CCACATGTCGTTCTTTGTCC | (AGA)5 | 2 | 0.37 | 52 | 130-140 |
| 194 | 16511 | F:CCCCCGACTTCCTAAAGAGA R:CGTGAAGGTGCTGAAGAAGTC | (AAG)7 | 4 | 0.51 | 52 | 130-150 |
| 195 | 16512 | F:TAAGCCCGACGCTTCTATTC R:GTGCCTCAGTTTCCGTTTGT | (AAG)5 | 5 | 0.47 | 52 | 135-150 |
| 196 | 16515 | F:CATCGGAATGGGATCAAAAG R:TTCAATCAACACTAGTTGAACAGC | (GTT)8 | 2 | 0.36 | 52 | 120-140 |
| 197 | 16517 | F:GCAGAGGAGACCAGTGAACC R:CAAGCTCAATCGACATCAACA | (CTT)5 | 2 | 0.08 | 52 | 135-155 |
| 198 | 16519 | F:CAAGTTTGCACCGTGAAATG R:TTTCCCAACACCCAGAGAAC | (ATC)6 | 4 | 0.55 | 52 | 130-160 |
| 199 | 16520 | F:TCAAGAACCCCAAAAACCAG R:CAGTCGAGACCTCAGCAACA | (ACC)5 | 3 | 0.27 | 52 | 135-150 |
| 200 | 16524 | F:CCAGAGGATGTGAACCAGGTA R:TTCAACCAAGCTGAACCCTTA | (CTT)5 | 4 | 0.47 | 52 | 135-150 |
| 201 | 16527 | F:ACCGGTTCCTCGAAAAACTT R:CAAGTTGGCATTCTCCCTACA | (ATG)5 | 2 | 0.19 | 52 | 135-150 |
| 202 | 16531 | F:AACCATGGGTTTGGTGTGAT R:CACATCCAATTCACCGTAAAGA | (TGA)6 | 2 | 0.24 | 52 | 135-150 |
| 203 | 16532 | F:CAATGGTGCCAATGTTGAAT R:GCTTCAAACATCCCAAGCTC | (TGA)5 | 2 | 0.14 | 52 | 135-150 |
| 204 | 16534 | F:TTGCAAATATACCAATTCCAAAA R:ATTGGAGCCTGGTGAAGACC | (GAG)5 | 6 | 0.51 | 52 | 120-150 |
| 205 | 16535 | F:TGTGAACATGCAGGGTAAGAA R:AACACATTGAGATTGAGCAATACAG | (GAA)6 | 3 | 0.36 | 52 | 130-150 |
| 206 | 16537 | F:GCTTCTGCATGCATTCAAATATAG R:TGAAATGGCTGCACTCTCTG | (ATG)5 | 2 | 0.37 | 52 | 135-150 |
| 207 | 16538 | F:AACACACCTCTCTCCATTTGTG R:GAGAAGAAGGCCGGAAAAAC | (AAG)5 | 6 | 0.61 | 52 | 130-150 |
| 208 | 16540 | F:TACGCTTGGCCAATTTATCC R:CCCTTCCAAAGGAGGGTTTA | (TCA)5 | 2 | 0.22 | 52 | 140-150 |
| 209 | 16541 | F:TCCATCACGGATAGCATCAA R:CTGCTGCGAGAGAGAGGACT | (GAA)5 | 2 | 0.31 | 52 | 140-150 |
| 210 | 16542 | F:AGTGGAGTGACCCGTAGTGG R:TCAACAACAAGTGTCGGATTC | (CTT)5 | 2 | 0.08 | 52 | 130-140 |
| 211 | 16543 | F:CCACCAACAAGAGGGAAAAA R:GGAGAGGGTTCCTCTTGTGTC | (CCA)7 | 2 | 0.29 | 52 | 140-150 |
| 212 | 16544 | F:TGGCTTGTTGCTGATACAGG R:TGTTTATGTTTATGTTTGCCATGA | (CAT)7 | 3 | 0.27 | 52 | 140-160 |
| 213 | 16548 | F:GGTGGTGCGAATAAACTTGAA R:CCTTCCAACTTGCCACTTTC | (AAG)5 | 2 | 0.14 | 52 | 140-150 |
| 214 | 16549 | F:CAATGAGATGCTGGCGATAA R:GTTCGGTGTTGTGGGTTTTT | (AAC)6 | 4 | 0.3 | 52 | 140-150 |
| 215 | 16550 | F:TGTCTTCGTTATTTTCTTCCTCTTC R:ATCGGTCAGCGCGACTATTA | (TCA)5 | 3 | 0.38 | 52 | 140-160 |
| 216 | 16551 | F:GGCTTCACATGACGCTGT R:GAGTGCCTCCAACCGATCTA | (GTG)5 | 3 | 0.53 | 52 | 140-150 |
| 217 | 16552 | F:GACGATGATTCCGAACAACA R:GAGGGATTCGTCGTCAACAT | (GAA)5 | 3 | 0.32 | 52 | 140-150 |
| 218 | 16555 | F:CTGCCTCATCCCATTCATCT R:TTACGGGTGACTTGTGTGGA | (TCA)8 | 5 | 0.59 | 52 | 130-150 |
| 219 | 16556 | F:GGTGGTCTTCCCCAGTACAA R:TTCCAATGCCTCTCCATCTC | (GGT)5 | 2 | 0.22 | 52 | 140-160 |
| 220 | 16558 | F:TGGTGTTCAGGCAACAACAT R:TTTGGTCATTCTGGGCATTT | (ATC)5 | 3 | 0.15 | 52 | 140-160 |
| 221 | 16559 | F:CCAATCTCCTGCCACGTTAT R:TGCCGTTTGAAGCTTTTTCT | (AGA)7 | 4 | 0.54 | 52 | 140-160 |
| 222 | 16561 | F:TGGATTGTTGTCGCGTAAGA R:CCGAGTTTCAGCCTTTGAAC | (AAG)5 | 2 | 0.14 | 52 | 140-150 |
| 223 | 16562 | F:AGCAACAATGGAAGCAACAA R:GGCTACCTCTGTGTGGGTATG | (AAC)5 | 4 | 0.46 | 52 | 125-150 |
| 224 | 16563 | F:CATCTCTCCATTTCACGGTTC R:ATTTCTGCCTGCATTTCACC | (TTC)5 | 2 | 0.08 | 52 | 125-150 |
| 225 | 16565 | F:TGAAAGGACAACCACCTCAA R:CCCCCTCTAAGCAACAACAA | (GTT)5 | 2 | 0.08 | 52 | 120-150 |
| 226 | 16566 | F:ATCGCTTGGTTTGGAATGTC R:TCATTACTCCCTTCATCTTCAAC | (GTT)5 | 3 | 0.12 | 52 | 140-160 |
| 227 | 16567 | F:GGAATCCAGCAGGAATAGCA R:GTGATTCCTGCCCATTATCG | (GAG)5 | 2 | 0.08 | 52 | 140-150 |
| 228 | 16569 | F:TGGCCCTAGTCCAACAATCT R:AACCGGGTTGAACCTTCTCT | (ACA)5 | 4 | 0.5 | 52 | 140-160 |
| 229 | 16570 | F:CAAACACCAACCACCACAGT R:AAGGGGAGACGAAGTGGAGT | (ACA)5 | 4 | 0.61 | 52 | 140-160 |
| 230 | 16571 | F:CAACAACTCCCCAACAACAA R:CCATGTAGTCAACCCCACCT | (ACA)5 | 3 | 0.5 | 52 | 140-160 |
| 231 | 16572 | F:CAAATCCTAAAGACTAAGTCCAATTTT R:GGAGCGCTTGAAGATGAAGA | (AAG)5 | 3 | 0.41 | 52 | 140-160 |
| 232 | 16575 | F:GCTACATTTCTGTGATGGATCTG R:TTCGGAGGCTTAGGACAGAA | (GAT)5 | 3 | 0.4 | 52 | 140-160 |
| 233 | 16577 | F:GAAAAGGGATTGTCCGATCA R:ACAAATTTTCCGAGCCAGTG | (CAA)5 | 3 | 0.15 | 52 | 140-160 |
| 234 | 16578 | F:CCGAAGAAGAACCCATTGAT R:CGCATTGCATTATTCCTCAA | (ATG)5 | 3 | 0.3 | 52 | 140-160 |
| 235 | 16579 | F:TCCAAACCGGTGGTTCTTTA R:GGATTTTGAGGCCATGTTTG | (ACC)6 | 3 | 0.49 | 52 | 140-160 |
| 236 | 16580 | F:CACCGAGCTCTAAGCCTCAA R:TGTGCTGTTGAGATGTTGTGA | (ACA)5 | 2 | 0.08 | 52 | 140-150 |
| 237 | 16582 | F:CCTCGACGGTTCTGATTAGG R:TCACAGCAACCCAATTTCAA | (GTT)5 | 4 | 0.51 | 52 | 120-150 |
| 238 | 16583 | F:TTCTGCCATAGCAGGGTCTT R:TCTTCCTGCACCAGAAGTCA | (CTT)5 | 6 | 0.72 | 52 | 125-150 |
| 239 | 16585 | F:TTGTGTTTGTGGGTGTGATG R:GACTTGCAGCCTTGGTTCAT | (ATG)8 | 3 | 0.21 | 52 | 140-160 |
| 240 | 16587 | F:GTGTCAGAGTGCGCGAGTAG R:TGCGTGATTTCCTCCTTTTC | (TGG)5 | 3 | 0.49 | 52 | 140-160 |
| 241 | 16588 | F:CGGTCTGAGGTTGTTGTGAA R:TTGTAAGACCGACTCGTCCA | (GGT)5 | 2 | 0.37 | 52 | 140-160 |
| 242 | 16589 | F:ATTCAACGCCAAGCTGAAGT R:ACTCTCGGTGGAGCAACAAT | (CCT)5 | 3 | 0.24 | 52 | 140-160 |
| 243 | 16590 | F:TATCAGCAACTGGTCGTCGT R:TCGGGTCTTCTTTGTCTTTGA | (CAA)6 | 2 | 0.33 | 52 | 140-160 |
| 244 | 16592 | F:TGGTGGAATGGGTGATGATA R:ACAACCACCACCCACAAATA | (TGT)6 | 3 | 0.46 | 52 | 130-150 |
| 245 | 16595 | F:TAGGCACTGGATCGAACCTT R:CGCCAGTTCAGAAACAACAA | (GTT)5 | 3 | 0.48 | 52 | 140-160 |
| 246 | 16596 | F:TCAACACGAGCCTTGTTCAT R:AACCATCGCAATCAAAAACA | (GAT)5 | 3 | 0.49 | 52 | 140-150 |
| 247 | 16597 | F:TGAGAGGCACCTCATCTTCA R:CAATGGTTGGCCCATTTAAC | (CTT)5 | 4 | 0.67 | 52 | 140-150 |
| 248 | 16600 | F:GGCAATAAGGAGGCGTCATA R:GCTCACGTTTCTCCGACTTC | (CAA)5 | 3 | 0.42 | 52 | 140-150 |
| 249 | 16603 | F:ATGTGGTGGTGGTGCTACAG R:GTTACCGCTTTCGGATCAAC | (TTG)6 | 4 | 0.57 | 52 | 140-160 |
| 250 | 16607 | F:CCAACTTCTACTTCACCAACCAT R:GTCCTCCCAATTTTGGACCT | (CTT)5 | 4 | 0.27 | 52 | 140-160 |
| 251 | 16608 | F:CTTCTTCCGTTGCACCATCT R:AAAGGAAAAAGGAATTCAGAGGA | (CTT)5 | 2 | 0.08 | 52 | 140-160 |
| 252 | 16609 | F:CGCAAAACACGACACACTTT R:GGCTTTTTGGTTGGAAGGAT | (CAC)6 | 2 | 0.11 | 52 | 140-160 |
| 253 | 16610 | F:GCGCCAGTCCAGCAATAATA R:ATCTTCTCTTGGGGCGTCTT | (CAA)5 | 4 | 0.19 | 52 | 140-160 |
| 254 | 16611 | F:CTCACGGGGAACACAACTTT R:ATCCAGCCACCTGAACTTTG | (CAA)5 | 2 | 0.24 | 52 | 140-160 |
| 255 | 16612 | F:CACACGAAAGATGGCAAGAA R:CCAGAGTCAAGATACCATAATCCAC | (ATG)5 | 3 | 0.51 | 52 | 140-160 |
| 256 | 16613 | F:TTGGAACCATCATCTCATTCTT R:GGAAAACGGTGGATTTTGAA | (AAC)7 | 3 | 0.34 | 52 | 140-160 |
| 257 | 16614 | F:AAGCACGAGGGACAAGAGTG R:GGATCCATCTGCATTGTCGT | (AAC)5 | 6 | 0.65 | 52 | 140-160 |
| 258 | 16616 | F:ATCATTGGCTTTGGCTTCAG R:CTGGCCAAAAGGCATAAGAG | (TCT)5 | 4 | 0.57 | 52 | 140-160 |
| 259 | 16621 | F:GCATTCTCTGCACTCTCACAA R:ATTCTGGCGAACGTTGAATC | (GAA)5 | 3 | 0.58 | 52 | 140-160 |
| 260 | 16623 | F:TCCTATTGGCTCATGTTCAATG R:TTGCCAAGCCTAAACCCTAA | (T)11 | 3 | 0.43 | 52 | 100-120 |
| 261 | 16624 | F:ACATGCACCACCTGATTTGA R:TGAAGAGGTGGGGAAACTATC | (T)10 | 3 | 0.55 | 52 | 110-120 |
| 262 | 16625 | F:ACCTGGCTTCACGCTAAACA R:ATCATCTTGTCTCGGGAGGA | (T)10 | 3 | 0.39 | 52 | 110-130 |
| 263 | 16626 | F:GGAGTTGCTATGCTTAGTGTGTG R:TGGGAAGCGATGAGTAGGTT | (T)10 | 2 | 0.14 | 52 | 110-130 |
| 264 | 16627 | F:TGCACCTTTCCATTCTCTGA R:CTAGCCGGTAAAACCACTGC | (T)10 | 3 | 0.42 | 52 | 100-125 |
| 265 | 16628 | F:CATGGGTGAGAACCTGTGGT R:GGTCAAGTGCCTCAAGTGGT | (C)10 | 6 | 0.53 | 52 | 100-125 |
| 266 | 16629 | F:GGGTGCCACCTTAGTTTGAA R:TGTGTTAGGACCCCAAGAATTA | (A)11 | 2 | 0.15 | 52 | 100-125 |
| 267 | 16630 | F:AAAGTTGCCAAAGCCAGGAT R:CAGCCTAGCGCGTTTCTGT | (A)10 | 4 | 0.5 | 52 | 100-125 |
| 268 | 16631 | F:TGAAGCAACAGCGTCAAAAC R:TTTTTCTTTGCTCTTTCTTTGATG | (A)10 | 5 | 0.72 | 52 | 100-120 |
| 269 | 16633 | F:CAGTCGCATTCACATTCACA R:TGCAAGTAACATAAACAAACATAATCA | (A)10 | 3 | 0.36 | 52 | 110-125 |
| 270 | 16634 | F:CATATTTTCTGCGGCATGTG R:GCGGGGTAGTCCAGTTTCTA | (A)10 | 3 | 0.4 | 52 | 100-125 |
| 271 | 16636 | F:CAGGCCCTCTTGATTGTGTT R:CCACACAAGCTCAGACTTAAGAA | (T)11 | 3 | 0.5 | 52 | 100-125 |
| 272 | 16637 | F:AATGCTACTGCAGGTGGTGA R:AGCCCACAAATGAAAAGAGA | (T)10 | 3 | 0.47 | 52 | 100-120 |
| 273 | 16643 | F:CAACCGATTGTCACCAAGTTC R:CTGGGAAAATTGCCCTCAT | (T)10 | 5 | 0.53 | 52 | 100-120 |
| 274 | 16644 | F:TGTTTGGGGATTCTGCTAGG R:GCCATTTAAACCAGCAATCCT | (T)10 | 3 | 0.26 | 52 | 100-120 |
| 275 | 16646 | F:GAAAGCATATAGGAATTCATTGTTCA R:TCATGGTTTGGTGTCACTGC | (A)12 | 4 | 0.6 | 52 | 100-120 |
| 276 | 16647 | F:TAGACATGCAGTGCCACACA R:TCGCAAGAAAATCAACCAAAC | (A)10 | 2 | 0.3 | 52 | 100-120 |
| 277 | 16648 | F:GTTGGGCTTCCCACAACA R:GCCCAACGAATCTTGGATAA | (A)10 | 2 | 0.14 | 52 | 100-120 |
| 278 | 16650 | F:CATGTTGGGCATGATGTTCT R:TGCCTATAAGGGCCATGTGT | (T)11 | 3 | 0.4 | 52 | 110-120 |
| 279 | 16652 | F:GAATGGGAATTGTCGGTGTT R:AGCCCTCTGAACCTGTGAGA | (T)10 | 2 | 0.37 | 52 | 110-120 |
| 280 | 16653 | F:TTCGAAGCTCTTTGTTTGTTTG R:CGTTGCGTTACCATGCTTATT | (T)10 | 2 | 0.19 | 52 | 110-120 |
| 281 | 16655 | F:CCGACTATGCGAAAGAGGAC R:TGCATCAACCTCCAGTACTCA | (T)10 | 4 | 0.44 | 52 | 110-120 |
| 282 | 16656 | F:GGCCTCTTATGGATGGTGTG R:CGTCAGCACCCTAAATCACC | (T)10 | 2 | 0.2 | 52 | 110-120 |
| 283 | 16657 | F:ACGCCATCCCTACAAATCCT R:TAGTGGCCTGCAGATCCTGT | (A)11 | 3 | 0.49 | 52 | 100-120 |
| 284 | 16658 | F:TCGATTGTCATGGTTGTTTTTC R:CGGACGAAGATCATGTGTGA | (T)11 | 4 | 0.61 | 52 | 110-130 |
| 285 | 16660 | F:TTTTGCTGAGGACGGAGTCT R:GATGCCTTGGGAACTTGAAA | (T)10 | 2 | 0.14 | 52 | 110-120 |
| 286 | 16662 | F:TGCATCAAATCATGGTGGTC R:ATCATCCTCGTCGTCACTCC | (T)10 | 2 | 0.37 | 52 | 110-120 |
| 287 | 16663 | F:TCGGGGTAAATGGAGCCTAC R:GCCAGAAGAACAAGGCGATA | (T)10 | 3 | 0.42 | 52 | 110-120 |
| 288 | 16664 | F:TCTGCAGAATTTGATCCATCTT R:TCCACCGCACAATGATTTTA | (T)10 | 4 | 0.55 | 52 | 110-120 |
| 289 | 16666 | F:TTTCCAATATCTTCTTCCACACC R:ATCCATGGGAAGGAGAATGA | (A)10 | 4 | 0.61 | 52 | 110-120 |
| 290 | 16668 | F:CGATGGGAATCGAAGTGAGT R:GCCTTTCACATTCACGTCCT | (A)10 | 2 | 0.14 | 52 | 110-120 |
| 291 | 16669 | F:CACCCGTTTGGACAAAACTT R:CACTTAAGGCAGATGGAATAGG | (A)10 | 3 | 0.48 | 52 | 110-120 |
| 292 | 16670 | F:TTCAGGGGAGCAAGTTATGG R:GGGCTCAACCTGAAAAGAGA | (T)10 | 3 | 0.48 | 52 | 110-120 |
| 293 | 16671 | F:CAGAAGGGGTTTGGAAATTG R:AAATCAACTACTACACCCTACACTCTC | (T)10 | 4 | 0.49 | 52 | 110-120 |
| 294 | 16672 | F:AAATCTGATGGGATTGAAATAAGC R:TTAAATTGGTTGCCACGTTTC | (T)10 | 4 | 0.63 | 52 | 100-120 |
| 295 | 16673 | F:TCGTGTTCCTCGTGTTGAGA R:TTTCCACCGTCTTCCTCAAT | (T)10 | 2 | 0.08 | 52 | 100-125 |
| 296 | 16674 | F:AGGAAAGGAACACCGAGTCA R:GACGATTCCCTTGGGAAGAT | (G)11 | 5 | 0.58 | 52 | 110-125 |
| 297 | 16675 | F:GCATTCCAGGTCTTGGGTAG R:TTGAAAAAGTGCTTATTTTCCAAAC | (G)10 | 3 | 0.47 | 52 | 110-120 |
| 298 | 16676 | F:CAAATAAAAATTAAAAAGCTGGTAACT R:GGTCATTCTCCGTTGCCTAA | (A)13 | 3 | 0.44 | 52 | 110-125 |
| 299 | 16677 | F:CCTGGTACAACAACCCATCA R:AGGTGATTGAATTGCATTGAGA | (A)11 | 4 | 0.53 | 52 | 110-130 |
| 300 | 16679 | F:CAAACCCATTACCCATGACC R:CACCTCATTCTCATCCTCTCCT | (A)10 | 2 | 0.28 | 52 | 110-125 |
| 301 | 16680 | F:AGCCCCTTTTCTTCACCTGT R:AGGGATGGGAAAGCTGTCTT | (A)10 | 3 | 0.26 | 52 | 110-125 |
| 302 | 16681 | F:GCCTGACAAGATGCACAGAG R:CTATCGCCGCAAGTCAATTT | (A)10 | 3 | 0.47 | 52 | 110-125 |
| 303 | 16683 | F:CCAAGATAAACGGTCATGATTTG R:CATGTTTGCTCACGCACCTA | (A)10 | 2 | 0.24 | 52 | 110-125 |
| 304 | 16684 | F:CTCACCACCACACCATTGTC R:CCAACCGTGGAGAAGAATTT | (A)10 | 3 | 0.4 | 52 | 110-125 |
| 305 | 16685 | F:GCGCGCTCAAAAATAAATTC R:GAACCCCTGTTCGATTCTCA | (A)10 | 2 | 0.14 | 52 | 110-140 |
| 306 | 16687 | F:AAAACAAAAACGTGGGGTGA R:TTCCATTTTGGCGCTATTTC | (T)10 | 3 | 0.48 | 52 | 110-125 |
| 307 | 16688 | F:GGCCATCTCGTTTCAGTACC R:GACTATGTGTGTGCATGAATTTGA | (A)12 | 3 | 0.41 | 52 | 110-125 |
| 308 | 16691 | F:TTTTTGAACCAACGCAAACA R:GGGACTGAAAAATATGGTTGTTG | (T)10 | 4 | 0.45 | 52 | 110-125 |
| 309 | 16692 | F:GATCCGTGTCCTTGAGGTGT R:CCATGCTCCAATGGTACAAA | (T)10 | 4 | 0.59 | 52 | 80-110 |
| 310 | 16693 | F:CCCCAAACTTTCCAAATCAA R:GGGTGATGGATGAGGAAGAA | (A)11 | 2 | 0.33 | 52 | 110-125 |
| 311 | 16694 | F:GCAACATGGACTAGGGATTTG R:GATTGGAAAGGGGCCATTAT | (A)10 | 3 | 0.44 | 52 | 110-125 |
| 312 | 16695 | F:CACAGGATCCAACACAGAACA R:GGAAGAAGCTGGATCCATTG | (A)10 | 2 | 0.35 | 52 | 110-125 |
| 313 | 16696 | F:GAAAAGGCACATCAGGACAAG R:CATTGTCTTCCCATGCATCA | (A)10 | 3 | 0.4 | 52 | 110-125 |
| 314 | 16697 | F:GTTCTGGGTGGAGGAAAGGT R:GAAGGACGTGAGGAGACCAG | (A)10 | 3 | 0.54 | 52 | 110-125 |
| 315 | 16698 | F:TTTCCTCTTTGTCACCCTTTTT R:TGGGGGTTTCTGCAAATAAA | (A)10 | 2 | 0.08 | 52 | 110-125 |
| 316 | 16699 | F:CAAAAGTACCGTTGCCTCAAA R:CCTCAGCTGGAACAATCCTC | (A)10 | 4 | 0.37 | 52 | 110-125 |
| 317 | 16701 | F:GCGAGATTGATCCAAAGAATTT R:TGTGAAATCATTGTCGTGTAAGA | (T)10 | 3 | 0.39 | 52 | 110-125 |
| 318 | 16702 | F:CGTGCTAAGACGAGTCCAATG R:AATTCACATGCGGAGGAAAC | (T)10 | 3 | 0.36 | 52 | 110-125 |
| 319 | 16703 | F:GGCAAAATGGGACATCTCAG R:GGTCCGGCTATGACTACACC | (T)10 | 2 | 0.33 | 52 | 110-125 |
| 320 | 16705 | F:TTTTTGCCATGTGGGTTGTA R:GGCCAAGATCTTTTTGGTGA | (T)10 | 2 | 0.11 | 52 | 110-125 |
| 321 | 16706 | F:TTCTTCATTGGAAAGTATCAGGTG R:CGTTATGCAATACCCCCTCT | (T)10 | 4 | 0.42 | 52 | 110-125 |
| 322 | 16707 | F:TCCAGCCCCACTACTTAGGA R:AGGAATGGGAGTTGGGTTTT | (C)10 | 5 | 0.66 | 52 | 110-130 |
| 323 | 16708 | F:TCCAAATCGTTCTCCCTCTG R:GGTGGTGTTTATTTTGGTGGA | (A)10 | 2 | 0.12 | 52 | 110-130 |
| 324 | 16710 | F:CATCCTTAGATGATCCCCACTC R:AATTAGTGGTGGCCTCCTTTT | (A)10 | 3 | 0.43 | 52 | 110-130 |
| 325 | 16712 | F:CGGCAGATATCGCAGGTATT R:TTCGTACTGGTGAACGACTG | (T)11 | 3 | 0.34 | 52 | 115-125 |
| 326 | 16713 | F:GAAGTGGTGGAGGGAATGAA R:CCATACCACCTTTCATTGATGT | (T)11 | 2 | 0.28 | 52 | 115-125 |
| 327 | 16717 | F:TAAGCCCTCCAAGCCTCATT R:GCTTTGGTGAGGGAAAGAGA | (T)10 | 2 | 0.14 | 52 | 140-150 |
| 328 | 16718 | F:GGGTGGCCCACTTTCTTACT R:TAGGTCGGGGGAAAGAAAAC | (T)10 | 2 | 0.14 | 52 | 110-130 |
| 329 | 16757 | F:CGCTCATTCTCTCTCAACTCC R:TTTTGCTGTGAGGGGAAGAT | (TC)6 | 2 | 0.08 | 52 | 110-125 |
| 330 | 16758 | F:CCCTTCAACAAAGCCTAACG R:AGGGTGCGAAGGAGGTTAGT | (CT)7 | 5 | 0.75 | 52 | 110-130 |
| 331 | 16759 | F:CAAAGCAATGCCACTCTTCA R:ACCGCGTTTACACACATTCA | (CT)6 | 2 | 0.08 | 52 | 140-160 |
| 332 | 16760 | F:CAATTGCATGAGCATTTCGTA R:TGAGAGCTCTGAGATTTTGTGTG | (CA)6 | 5 | 0.68 | 52 | 140-160 |
| 333 | 16863 | F:TGCAGGTAAAAGAGCATTTCA R:TTGTTGGGGTAAGCATGTCA | (AC)8 | 4 | 0.56 | 52 | 120-140 |
| 334 | 16864 | F:GGATCAGGTGCTGCAGTTTA R:TAACGTTTGCACCTCCCTCT | (TC)6 | 3 | 0.37 | 52 | 130-150 |
| 335 | 16865 | F:ATTTTATCGCTTCCCCCACT R:TGAATCCGAAGAGAGCATGA | (TC)10 | 4 | 0.44 | 52 | 120-140 |
| 336 | 16866 | F:CAATGATGTTTTGGTTAATGTGGT R:CAAGGAGCAAGAGGGACAAG | (GT)6 | 4 | 0.62 | 52 | 130-140 |
| 337 | 16867 | F:TGGTTCCCATGGAAAAGAAA R:CAATGAGGGGAGGAATTTCA | (GA)9 | 4 | 0.52 | 52 | 130-160 |
| 338 | 16868 | F:TGGGATAAATCACGATAAGAATTG R:CCACTCCCATCTCTCGTTGT | (GA)10 | 6 | 0.67 | 52 | 130-150 |
| 339 | 16869 | F:ACCCGCAACAACATAACACA R:GGAGCCGTCAAATTCAAGAG | (CT)9 | 3 | 0.26 | 52 | 130-150 |
| 340 | 16870 | F:TGTCACACGCGGTAGAAGAG R:GCGAAAAATGATATTGAAGTGTT | (CT)7 | 4 | 0.36 | 52 | 130-150 |
| 341 | 16872 | F:TGAAGGCCAAACCAACCTAC R:TGGCTTTCATGGTGAACTTG | (AG)8 | 4 | 0.41 | 52 | 130-155 |
| 342 | 16873 | F:TGAGCACTCTTGCATTGACA R:TCATGGCTTTCGATTCCACT | (AG)6 | 4 | 0.33 | 52 | 130-150 |
| 343 | 16874 | F:TTTTTCACACGCAACAATGA R:ACGGGTGCTCTTGGTATTTG | (AG)15 | 6 | 0.65 | 52 | 130-150 |
| 344 | 16875 | F:ATGCATAGCCTGGCGTATTT R:TTTTGGGGATCCCAATAGAAT | (AG)10 | 5 | 0.59 | 52 | 130-150 |
| 345 | 16877 | F:TGGACAATTCCACATGAACC R:CACGCACATACGACCACAC | (TG)8 | 4 | 0.53 | 52 | 130-160 |
| 346 | 16878 | F:CCATTTTCACGCAACCTTTT R:TGGAGTGTTTGCTTGGAGTG | (TC)6 | 3 | 0.42 | 52 | 130-150 |
| 347 | 16879 | F:TCCGAAGTTCGCCTCTTTTA R:CCTACACCGAGATTCAAATGGT | (TA)6 | 3 | 0.57 | 52 | 130-150 |
| 348 | 16881 | F:ATGGGCTTTAGGGGAAGAAA R:AAAAGCAGCACATGGAGGAC | (GA)7 | 5 | 0.5 | 52 | 130-150 |
| 349 | 16888 | F:TTCGATCAAAGGGGAATGTC R:TCCATGTTGGTTGGTAGTGG | (TC)8 | 4 | 0.56 | 52 | 130-150 |
| 350 | 16892 | F:CTCTTGGCAGGGAATTTGAC R:GAAGCAACCCTTTCCTCTCC | (GA)6 | 3 | 0.53 | 52 | 130-150 |
| 351 | 16893 | F:TTGCCAGCTATCACCTAACG R:CCCCAGAGGAGAAGGGTAGT | (CT)6 | 3 | 0.15 | 52 | 130-150 |
| 352 | 16896 | F:ATATGAGGTGGTGGCTGACC R:CGGAGGGTGATCTCAAAGAA | (CT)6 | 2 | 0.37 | 52 | 130-150 |
| 353 | 16899 | F:GCCTCCTTCGATACCAGATG R:GCCTCCAAAAGCTCCAAAAT | (AG)8 | 3 | 0.49 | 52 | 130-150 |
| 354 | 16901 | F:CCACACGTCCTCCTCTATTCTT R:CCATTTCTGCAATTCGAGTG | (TC)7 | 3 | 0.36 | 52 | 130-150 |
| 355 | 16907 | F:TTCTCCGACAAGGGTGATTT R:TTCACGCCTCTCTCTCCATAA | (CT)6 | 3 | 0.5 | 52 | 130-150 |
| 356 | 16908 | F:TACATGGTCGATTGGGGAAC R:ATGGTTGAGCCACCAACTCT | (CT)12 | 3 | 0.27 | 52 | 130-150 |
| 357 | 16910 | F:GGGAAGACGAAAGCTTCACA R:CAACACTCGACCAACACCAG | (AG)6 | 2 | 0.14 | 52 | 130-140 |
| 358 | 16911 | F:TTCCAATACCAAACCCCTTTA R:AACTCGATCGTCTCCACCAG | (CCA)5 | 2 | 0.08 | 52 | 140-160 |
| 359 | 16912 | F:AATGAAACCGCCGTTGTTAG R:TGTTGTGGTTGTTGATAATGTTGA | (CAA)5 | 2 | 0.14 | 52 | 140-160 |
| 360 | 16914 | F:AACCTCGAGCAACAACAGGT R:TTAGGTTGGCGTTTTTGGTC | (ATC)6 | 4 | 0.46 | 52 | 150-170 |
| 361 | 16916 | F:GGTGCTGAAGAGCTCAGAGG R:CTTCAGCTTGGACTGGCTCT | (TCT)7 | 2 | 0.08 | 52 | 150-160 |
| 362 | 16917 | F:GAATGGAGGTTGAAGGTGGA R:CAAGCTTCTAATCTCACTCTCACAA | (GAA)5 | 5 | 0.64 | 52 | 140-160 |
| 363 | 16919 | F:ATGTGCATGTTCCTCCTCCT R:CACAAACAGTGCCACACTCA | (ATG)5 | 2 | 0.36 | 52 | 145-155 |
| 364 | 16920 | F:CATCACCGAAGGACTGTGAA R:CATTCTCTGCCTCGATTGAA | (AAC)6 | 3 | 0.15 | 52 | 150-160 |
| 365 | 17056 | F:AGAAACCAGCCTCCACCAG R:TGTTTGTTTTCCGACGATGA | (AAC)5 | 5 | 0.63 | 52 | 160-175 |
| 366 | 17057 | F:CTTCCTCTTTTTGGCGTCAC R:CGACATGGTCGAAGAAAACA | (TGT)5 | 2 | 0.08 | 52 | 160-175 |
| 367 | 17059 | F:GATAGGACTGCAGGCGAAAG R:TTGTGGTTGAGGAGCTACGA | (CAA)5 | 3 | 0.54 | 52 | 160-180 |
| 368 | 17060 | F:CAGCATGCTCAATCACCACT R:AAAACATGGCAACCCTTCAG | (AGA)8 | 5 | 0.65 | 52 | 160-180 |
| 369 | 17063 | F:TGCTGTGTGGAGTGGAAGAC R:CGATGACATCCTCGGCTAAG | (AAC)5 | 4 | 0.63 | 52 | 165-190 |
| 370 | 17064 | F:TCAACATCCAAAGTGGGTGA R:AACAAGACAATCAAGCTGGAAAT | (TTG)5 | 2 | 0.09 | 52 | 165-190 |
| 371 | 17066 | F:TGGGATGAAAATGTTATGAATG R:CAAAACCACCCTTTCCGATA | (TGG)7 | 4 | 0.65 | 52 | 160-180 |
| 372 | 17068 | F:GAAAGGCTGTGGCTTGTCTC R:CAACATGAATTTCCCCCTACA | (TCT)5 | 3 | 0.18 | 52 | 160-180 |
| 373 | 17070 | F:AAGGAGTGAACATTGGTTTTCAA R:CACTTTCCACATGCCCTGTA | (GTC)5 | 3 | 0.12 | 52 | 160-190 |
| 374 | 17071 | F:TCCTCCCAGTCAACAACGTA R:TAAGGAGGCCCAACCATCTA | (CTT)7 | 3 | 0.37 | 52 | 160-190 |
| 375 | 17073 | F:TTGGAATCCTTGGCCTATCA R:TGGCATAATCTAGGCATTGTTTC | (ATG)5 | 3 | 0.32 | 52 | 150-175 |
| 376 | 17074 | F:CGACGAACAAACGAATTCAA R:TCGACCTCGATCCAACTTTC | (ATG)5 | 2 | 0.08 | 52 | 160-180 |
| 377 | 17076 | F:TGCCTCATGCTTTAGCTGACT R:TTGATTTTGTTTTGTTGTTGTTGTT | (ACA)5 | 7 | 0.63 | 52 | 155-185 |
| 378 | 17077 | F:CAAAAGGCCCTTCAATTGTT R:TTTTGTCTTCTTCATTCATATTGCTTA | (AAG)6 | 3 | 0.23 | 52 | 165-180 |
| 379 | 17079 | F:TCCTCATGTTGGTCGTAGCA R:GGGAGGACTCTGATCCTGGT | (GTT)5 | 4 | 0.38 | 52 | 165-190 |
| 380 | 17080 | F:CAACATCAACAACATTGCAGAA R:CCGGGAGTTGTAGGAATTGA | (GAA)5 | 2 | 0.22 | 52 | 165-190 |
| 381 | 17081 | F:GAACAAAGACACCGCAAACA R:TCCTCTTCCTCCTCTTCCTTTT | (GAA)5 | 3 | 0.5 | 52 | 110-125 |
| 382 | 17085 | F:ATGAGGAAGCACCCATAGCA R:CACTCGCTCCACAAACTCAA | (ATG)6 | 3 | 0.4 | 52 | 160-175 |
| 383 | 17087 | F:CCACTTCCCACAGAAACACC R:GGGGAGCAAAAACAGTTTGA | (ACA)8 | 3 | 0.58 | 52 | 150-175 |
| 384 | 17088 | F:TGCAGGACACCAGAAGAAGA R:GCGAAGAACACGTCTTTACAA | (ACA)7 | 3 | 0.45 | 52 | 160-180 |
| 385 | 17089 | F:CAATATGAACAACGTCATCAACAA R:CGTTGGTGGTGGTCCTAACT | (ACA)5 | 4 | 0.58 | 52 | 160-180 |
| 386 | 17090 | F:AGCCAAAGTGCGAGAAAAAG R:TCATGTCATCATGCCTAAACCT | (AAT)6 | 2 | 0.08 | 52 | 160-175 |
| 387 | 17093 | F:GTGCCAACGAAGGTTTCATA R:TCTCAAGGGGTTCAGCATCT | (TTC)5 | 3 | 0.48 | 52 | 160-190 |
| 388 | 17097 | F:GGAAGGACCAAAAAGCACAA R:TGGTCCATACAATGCAGTTCA | (TGA)5 | 3 | 0.32 | 52 | 160-180 |
| 389 | 17098 | F:GAGGCTAGAGGCTCGTATGG R:CGCAACAACGTGTGTTACTTC | (GCT)5 | 3 | 0.37 | 52 | 150-180 |
| 390 | 17099 | F:TCACACTTCCATTGCCACTC R:GTTCAGGCAATGAGGGAAAA | (CAT)6 | 4 | 0.31 | 52 | 160-180 |
| 391 | 17101 | F:AACCCACCATGGCTTAATCA R:CAGCAGAAGCGGATGTATTG | (CAT)5 | 4 | 0.32 | 52 | 160-180 |
| 392 | 17102 | F:TCTGGTCATGTTCCTTTGATTC R:TCTCCGAGGGAGATTCTTCA | (CAC)5 | 3 | 0.38 | 52 | 160-180 |
| 393 | 17154 | F:CGGACCAATCCTTTTTCAGA R:TGAATGAGGAGGATTCACCAT | (ATC)7 | 2 | 0.33 | 52 | 170-190 |
| 394 | 17156 | F:TCGACCAAGCTCTGTCACTG R:TCGGTTTCTCGTTCTCTGGT | (ACA)7 | 3 | 0.57 | 52 | 170-190 |
| 395 | 17157 | F:TGAGCTGTACAGGACAACAGC R:TGAAGGGCATGTAGGACTGG | (AAT)5 | 2 | 0.33 | 52 | 90-110 |
| 396 | 17158 | F:CTCCCGAGTCTTGGCTAATG R:AGGCGCTCATAAACAGTTCC | (TTG)6 | 2 | 0.37 | 52 | 170-190 |
| 397 | 17160 | F:TTGCGGCAACAGTACAGAAG R:ACTCTTGCAAAAGGCATGGT | (TGA)5 | 3 | 0.5 | 52 | 175-200 |
| 398 | 17161 | F:GGCTTCTTGGTCGATGTTGT R:ACATATCGTGCCGCATATCA | (GTT)5 | 3 | 0.42 | 52 | 170-190 |
| 399 | 17163 | F:TTGCGCCAACAGAATTACAG R:CGAACAGGGAAGGAAAACAG | (CAC)6 | 3 | 0.4 | 52 | 175-190 |
| 400 | 17164 | F:CAGCATTGGAATGCACAAAC R:AATCGTAATGCTGCTGCTTCT | (CAC)5 | 3 | 0.32 | 52 | 170-190 |
| 401 | 17165 | F:ACCACAACAACAGCAACCAC R:GAGGAAGAACCGCAAGTGAG | (CAA)6 | 2 | 0.14 | 52 | 170-190 |
| 402 | 17166 | F:CACCCTCCTTCACCTTCAAA R:GCTTCTGGAAAATGGGTGAA | (AGT)5 | 3 | 0.4 | 52 | 175-200 |
| 403 | 17168 | F:CACTTCTTTTAATCAAGCGCAATA R:TGGTCATGTGTTTGGCCTTA | (TCA)5 | 3 | 0.29 | 52 | 175-190 |
| 404 | 17169 | F:AGACGTTACAGGCCCGTCTA R:CGCAGCAATGTGTGCTACTT | (GCT)5 | 2 | 0.08 | 52 | 120-140 |
| 405 | 17171 | F:CCTCCTGCTTCCTCTTGATG R:AGTAATAGCCACGGCCACAG | (CCA)5 | 3 | 0.21 | 52 | 175-190 |
| 406 | 17173 | F:GAGGACATGGAGCAACACCT R:CGCTCCTCATTTCCTTCATC | (AAG)8 | 5 | 0.65 | 52 | 170-190 |
| 407 | 17175 | F:TGGGGACTACTGTGTGTGTCA R:CCACTTTTGAACCCCAGATG | (TGT)5 | 2 | 0.31 | 52 | 100-120 |
| 408 | 17177 | F:ATTGTTGAGGCGAAACATGA R:TGGTGAGGCTTATGATAACTGC | (CCA)5 | 4 | 0.56 | 52 | 175-190 |
| 409 | 17179 | F:TGTGGCCATAGCATGAGTGT R:TAATTCCCTGCCAGAACCAC | (AGA)5 | 3 | 0.39 | 52 | 175-190 |
| 410 | 17180 | F:TTCACACCTGGCTCACTCAG R:CTGTCTTGGCCGACACTGTA | (AAC)5 | 3 | 0.15 | 52 | 175-190 |
| 411 | 17181 | F:AGTCCTCACAACGCCATCTT R:GCCCACTGTCTCAACCTTGT | (TTG)5 | 5 | 0.57 | 52 | 175-190 |
| 412 | 17186 | F:CCCTTCATGGAAAACTCAAAA R:TCCATCTTCCTGAGGGTCTTT | (CAA)6 | 2 | 0.26 | 52 | 175-190 |
| 413 | 17189 | F:TAGAGGGAGGTTCCGACGAT R:AAACCCAACCTAGCGATTCA | (ACA)5 | 3 | 0.43 | 52 | 150-180 |
| 414 | 17190 | F:GGCGAAGCGAATCTAACATC R:CCACCTGCTCAATTCCAAAT | (AAG)8 | 4 | 0.62 | 52 | 160-180 |
| 415 | 17191 | F:CGACAATGGACCTTGAATGA R:TTCTTCTTCTTTCTGATTCTTCCTC | (AAG)5 | 3 | 0.15 | 52 | 170-180 |
| 416 | 17192 | F:TTGCTATTGCTTCCCGCTAT R:ATATCCGGTTGAGCAACGAC | (AAC)5 | 3 | 0.47 | 52 | 170-190 |
| 417 | 17193 | F:CACAGCCATACCCAAGTTACAA R:GGTTGCGAGGGATGAGAATA | (AAC)5 | 3 | 0.43 | 52 | 170-190 |
| 418 | 17196 | F:CAGTGGGGGAGAAAGCTACA R:TGAGGGAATCTGGACTCTTG | (GAA)5 | 5 | 0.39 | 52 | 170-190 |
| 419 | 17197 | F:CTCGCCAGCATCATCAAGTA R:CATCGGAATGGGATCAAAAG | (CAA)6 | 4 | 0.5 | 52 | 170-190 |
| 420 | 17199 | F:AAAATAGAGATGCAGAAAGAATAAAAA R:TGCATAGGCATCTTGTCCAC | (AAT)5 | 4 | 0.47 | 52 | 170-190 |
| 421 | 17201 | F:CTATGTTCCGCAAGCAGGTT R:GCATTAAAGCAAGCCATCAA | (TTG)5 | 3 | 0.39 | 52 | 180-200 |
| 422 | 17203 | F:CCTGGAAAGCCTTTGAATGA R:CAGGTATGGCTTTTGGCACT | (GCA)5 | 5 | 0.47 | 52 | 180-200 |
| 423 | 17205 | F:CACAGAGATCCCTTGCATCA R:AAGCCACGTGCAGAGGTAAG | (CCG)6 | 4 | 0.37 | 52 | 180-200 |
| 424 | 17206 | F:AAGCACGAGTGCAGGTTGTA R:TTGAATCATGGTTTGGATTGA | (ATC)6 | 3 | 0.29 | 52 | 180-200 |
| 425 | 17208 | F:AGATGTTCTGGTTTGGGTTGA R:ATCACCGCTTCAGGTACCAC | (AAG)5 | 2 | 0.37 | 52 | 180-200 |
| 426 | 17210 | F:TTCGGAGACGAGTTCCATTT R:CTCGTATCGTCGAAGGTGGT | (TAG)6 | 3 | 0.3 | 52 | 180-200 |
| 427 | 17211 | F:CTGTTCGCTTTCCGTTCAAT R:GCGAGAACAATGCGATTATG | (GAA)5 | 2 | 0.14 | 52 | 180-200 |
| 428 | 17212 | F:GCCTCTTAACCGGCATTGTA R:TCAGGAGTGAGGAGGCAAGT | (CAT)6 | 2 | 0.14 | 52 | 180-200 |
| 429 | 17217 | F:GGTTTGATGACGGGCTTATG R:GCAGTTGTGTTGGTGCAATAG | (ACC)6 | 3 | 0.35 | 52 | 180-200 |
| 430 | 17218 | F:GCAACATGCACCCTAACCAT R:GCGGAGTTGGATCTTGTTGT | (ACA)5 | 3 | 0.27 | 52 | 180-200 |
| 431 | 17219 | F:TCATGTGCATGTGATGAAGAAA R:GGTGTACCCATGTGCCATTT | (AAC)9 | 5 | 0.66 | 52 | 180-200 |
| 432 | 17220 | F:CAGCTTCATTTCCTCCATCA R:GGTGACGGTGTTGATGTTGT | (AAC)5 | 4 | 0.42 | 52 | 180-200 |
| 433 | 17221 | F:TCCTCTCCATGCACTGTACG R:TGTGGTGTTTTATGCGTGGT | (AAC)5 | 3 | 0.53 | 52 | 180-200 |
| 434 | 17224 | F:CCACGCTCCAGTACACTCAA R:GCCACGACATTCTCATCTCA | (CTT)5 | 3 | 0.43 | 52 | 170-190 |
| 435 | 17225 | F:GTTGCAAGCTGCTACCATCA R:AGACGGATCCAACAATCTCC | (CCT)5 | 3 | 0.5 | 52 | 180-190 |
| 436 | 17226 | F:GATCAGCAGCAACCCTTGAT R:CCATTTGCCTCACAGATTGA | (CCA)5 | 2 | 0.33 | 52 | 180-195 |
| 437 | 17228 | F:CGCAATTCACCTCAACAGAC R:GGGAAGGCGATGATGAAGTA | (AAC)5 | 4 | 0.62 | 52 | 180-200 |
| 438 | 17230 | F:TGGGAGAAGGTTTGGTTTTG R:ACCCAACTTTCCCTCCATCT | (TTG)6 | 4 | 0.61 | 52 | 180-200 |
| 439 | 17233 | F:CCAGTTTCAACAACCGTTCC R:TGTCTCTGGTTGCGATTCTG | (AAC)5 | 3 | 0.52 | 52 | 180-200 |
| 440 | 17241 | F:GGCCGAAGATTTCAACAAAA R:GAAGCAGTTGAAGCTCACCAT | (TTC)8 | 4 | 0.48 | 52 | 180-200 |
| 441 | 17244 | F:TTGTTCCAACGCCAGTAACA R:TGACCTTTTCAGCCCACTCT | (TCC)5 | 5 | 0.4 | 52 | 180-200 |
| 442 | 17245 | F:TTCCATCATCAACATCATCATC R:GGTTGTTGACGGACGAAAGT | (TCA)6 | 4 | 0.51 | 52 | 180-200 |
| 443 | 17343 | F:AGTAGTTGGGCCCTGAGGTT R:ATAAAGTTGCCCCAGGAGGT | (TTC)5 | 3 | 0.47 | 52 | 150-160 |
| 444 | 17344 | F:AACCCCAGGACAACTCACAA R:GTTTCCCCATCAGGGTTTCT | (TTC)5 | 3 | 0.51 | 52 | 130-150 |
| 445 | 17352 | F:TGATACCAAAACCGCACTCA R:TCTTCACTGGAGCTGCTGAA | (AAC)5 | 3 | 0.46 | 52 | 190-200 |
| 446 | 17353 | F:TTTGCACTCTTTGGGACATTC R:ATCGCGAAAGACAGCAATTC | (TTG)5 | 3 | 0.34 | 52 | 190-210 |
| 447 | 17360 | F:CGGTGACATTTGTTCGTCTG R:CGGTTGCATCTCTCAAACCT | (GTT)5 | 3 | 0.51 | 52 | 190-210 |
| 448 | 17361 | F:AGGGTTTTGGGCTTGTTCTT R:CCCACCTCCATCATTCTTTG | (GAT)5 | 2 | 0.08 | 52 | 190-210 |
| 449 | 17362 | F:GAGAAACTCGTGTACGGAAACA R:TCTTCTTCCACTGAGGCGATA | (GAG)5 | 2 | 0.37 | 52 | 190-200 |
| 450 | 17364 | F:ACCGAATAACTTCCGCACAG R:TTTTTGTCAAAGGGATGTTTTT | (CAA)5 | 3 | 0.15 | 52 | 180-200 |
| 451 | 17365 | F:ATGTGAGCGAAAACGGAATC R:CACCACCCCATCATACAACA | (AGA)5 | 4 | 0.37 | 52 | 190-210 |
| 452 | 17368 | F:GAGGACATGGATTTTGCTGAG R:GCACCAGGAGCACCTATGAT | (TTG)5 | 2 | 0.08 | 52 | 190-210 |
| 453 | 17370 | F:TTCTGCAGCTATGCGCTCTA R:AGCTGGAGAGAGGCTACAGG | (TCT)5 | 5 | 0.53 | 52 | 180-210 |
| 454 | 17371 | F:CACCACCACCTTGAACTGAA R:ATTGGCAAAGGAGGAGGAGT | (TCA)5 | 2 | 0.37 | 52 | 190-200 |
| 455 | 17380 | F:TAGGCCAGCTCCAACACTTT R:CCAACTTGTCAGTCTGATGAATCT | (TCA)5 | 4 | 0.55 | 52 | 190-210 |
| 456 | 17381 | F:CCTTTGATGATCCTGCTGGT R:TCAAGTTGGGCCTTCAAATC | (TAT)6 | 4 | 0.45 | 52 | 190-210 |
| 457 | 17387 | F:GGCAAAGGAGGCCTTAGATT R:CTCACAATTGCGGGTGATT | (ACA)6 | 5 | 0.28 | 52 | 190-210 |
| 458 | 17388 | F:CCGTGTTGGTGCAGAATATG R:ATACCCGACGAGGAGGCTAC | (TTC)6 | 3 | 0.43 | 52 | 190-210 |
| 459 | 17389 | F:TGTTCACTTTTCCTTTTCTCACA R:AAGAGGGAGGATCTGGTGGT | (TTC)5 | 2 | 0.36 | 52 | 190-210 |
| 460 | 17395 | F:TTTCTTGTAAATTGCACATCAAG R:CAATGCGGGAATTTTCATTT | (TAA)5 | 4 | 0.42 | 52 | 190-210 |
| 461 | 17397 | F:TGCTGCTCAGAAACAACCAT R:GTCGGCATAAAAGCAAAGGT | (CTC)5 | 3 | 0.48 | 52 | 190-210 |
| 462 | 17399 | F:TATCAGGCCCTCGAAGATGA R:GGTTGTTCCAGTCGAAGGAG | (AAG)6 | 2 | 0.11 | 52 | 190-200 |
| 463 | 17403 | F:GCATGGCAGAGATCCAATTT R:GTTTGATCCAACGGCTAGGA | (TGG)6 | 3 | 0.31 | 52 | 190-210 |
| 464 | 17404 | F:ACCTTCAAGGCGGAGATTTT R:TCCACACACAACAACGACAA | (TCT)5 | 2 | 0.14 | 52 | 190-210 |
| 465 | 17405 | F:CATTGCATCCCTCCATCTTT R:CATCTCCAGCGATGCTACAA | (TCT)5 | 3 | 0.48 | 52 | 190-210 |
| 466 | 17408 | F:CCGTGCTGCAAATACTTCAA R:TATGTAGCCGACCCCACCTA | (CAA)5 | 2 | 0.28 | 52 | 190-210 |
| 467 | 17409 | F:TTTCTCCGCAACCCTAAAAA R:TGTTGTCAAAATCCACGATCA | (CAA)5 | 2 | 0.34 | 52 | 190-210 |
| 468 | 17410 | F:TCAGCCACACAACACAAACA R:TGTGAATGAAGTTTGGGATCA | (CAA)5 | 3 | 0.46 | 52 | 195-220 |
| 469 | 17411 | F:AGGAGGCCACATGTCAAAAG R:AAGATACCTTCTTCCTCCCAGAA | (ACA)5 | 4 | 0.5 | 52 | 180-210 |
| 470 | 17412 | F:CCATCATCTCCCACATCTCA R:GAGGTGGAATGGGTGAAAAA | (ACA)5 | 4 | 0.48 | 52 | 180-210 |
| 471 | 17413 | F:GCATCCAAGGGGTTCCTAAG R:TGAATCCTCTCCTTCAGTGACA | (AAG)6 | 3 | 0.24 | 52 | 190-210 |
| 472 | 17415 | F:TGGGGTCAAAAGAGACCTTCT R:GAGGCCTCCTGTCAGAAAAA | (TGA)5 | 2 | 0.14 | 52 | 200-210 |
| 473 | 17416 | F:ATCTTGGGCACCATGAAAAC R:GCGGCGGTAACTAACTCAGA | (TCA)6 | 4 | 0.27 | 52 | 200-210 |
| 474 | 17418 | F:TGGAGGAAGCCTCTTCTGAG R:TCCAGCTAGCTTCCCACTGT | (GAT)5 | 3 | 0.21 | 52 | 190-210 |
| 475 | 17422 | F:ACCACAAATGCTTCCGCTTA R:GTTGTTGTTGCTGCTGCTGT | (AAT)5 | 5 | 0.71 | 52 | 190-210 |
| 476 | 17423 | F:TTCGAGTTTCACCATGTCCA R:GTCGGTTCCTTCATCGTCAT | (TGG)7 | 2 | 0.33 | 52 | 190-210 |
| 477 | 17424 | F:GAGAGCATTCAAGGATGTCCA R:CATGCCACCAGATGCTAGAG | (TGG)6 | 3 | 0.45 | 52 | 190-210 |
| 478 | 17427 | F:ACCGGATTGTGGATAACCTG R:TAAATGTTGTCGCCATCACC | (TAA)5 | 3 | 0.43 | 52 | 190-210 |
| 479 | 17428 | F:TTGAAGAAAAACCCCCAGTG R:GCAATCAGACCCCACTCAAT | (CAT)5 | 2 | 0.08 | 52 | 190-210 |
| 480 | 17431 | F:TTCACAATTCACCACCAATCA R:CCAACGTCAGGTACGATTCA | (AGA)9 | 6 | 0.7 | 52 | 190-220 |
| 481 | 17433 | F:TTCAGCAAGATAAACCATTCACA R:TGAAGATGGTGGGAGGTAGC | (AAC)5 | 4 | 0.27 | 52 | 200-210 |
| 482 | 17434 | F:AGCAGTCGAAAGGAAACCAC R:GCTTTCGTCTTGCCTGAAAC | (AAC)5 | 3 | 0.12 | 52 | 100-110 |
| 483 | 17435 | F:TGGATGGAGCTGAAATTGTG R:CGGCGAAAGAGAGAGAAATG | (TGT)5 | 2 | 0.14 | 52 | 190-210 |
| 484 | 17436 | F:TGCAGCAAACTGGACAAGAA R:TCTTCTTCTGAAATGATCAATGAAA | (TGT)5 | 3 | 0.21 | 52 | 190-210 |
| 485 | 17437 | F:AGGCTTCATCTGCTCCAAAA R:AACCCTTCAAAACAACAACAAAA | (TGA)6 | 3 | 0.32 | 52 | 190-210 |
| 486 | 17438 | F:GTCATGCCACGAGGGATATT R:TGATGGTCCTTAAGGGTTTTG | (TCA)5 | 3 | 0.4 | 52 | 190-210 |
| 487 | 17496 | F:TCGAAGTTCCATAACAACACAGA R:GTGGTGAACGCGGTAGATTT | (GAA)5 | 3 | 0.4 | 52 | 200-220 |
| 488 | 17499 | F:CAATGGCTGGTTTGTTCTCA R:CTGTTGGTGTTGTTGGTGCT | (ACA)5 | 3 | 0.25 | 52 | 200-220 |
| 489 | 17503 | F:GGATGGTGGGTCCTTTGTTT R:AAGTGAGTGTTTCGTTGTGAGAA | (TAG)5 | 4 | 0.28 | 52 | 200-215 |
| 490 | 17506 | F:TCACTGACGTTCCCACTCTG R:CGTCGCTGTCAGTTCCAGTA | (CCA)6 | 4 | 0.27 | 52 | 190-210 |
| 491 | 17511 | F:TTTCTGGGCTAAGGGTTGAA R:TCACCCTCTCCCAAACTCTC | (AG)6 | 2 | 0.29 | 52 | 130-150 |
| 492 | 17513 | F:TGTTCCTCTCCTCTCTCAGGTT R:TGCATAGGGAGGTTGAGGTT | (TC)6 | 7 | 0.79 | 52 | 130-150 |
| 493 | 17514 | F:CCGATTGCTAAAGCGTCTCT R:GCATCAAAACCGTTGGATTA | (GA)8 | 4 | 0.48 | 52 | 130-150 |
| 494 | 17517 | F:CACTCCCCAAACCTCTCAAA R:GCTTAGGAAGCAAGCAGTGG | (TC)6 | 6 | 0.74 | 52 | 130-150 |
| 495 | 17518 | F:GCCCTGAAAGGCTAAAGGAT R:ATCGAAAGCAGGTGGAGTTG | (TC)11 | 5 | 0.45 | 52 | 135-155 |
| 496 | 17520 | F:TTCAATTCCCTTCCCACATC R:CCTGGCAGGTTTATTCAAGAAG | (CT)8 | 4 | 0.48 | 52 | 135-150 |
| 497 | 17521 | F:CGGGATGACCGCTATATGAT R:ACATTTCCGCAAAGCAAAAT | (AG)7 | 3 | 0.15 | 52 | 135-150 |
| 498 | 17523 | F:GACGGTCTCCGGATAAATCA R:CCATGGACAAGGTTGGTTTT | (AC)6 | 3 | 0.46 | 52 | 130-150 |
| 499 | 17524 | F:TGCAATTGAATTTTCACAATGAT R:TTGCTTGCATGAACACTTGA | (AC)13 | 8 | 0.68 | 52 | 125-150 |
| 500 | 17525 | F:TGTCGGGCAAAATCAGTACA R:TTTGCCAGTGGAAGTGATGA | (TG)6 | 5 | 0.53 | 52 | 140-160 |
| 501 | 17526 | F:CAAGCTTCCATGCTCAACCT R:AGCCAGGCTTGGAATGACTA | (TG)6 | 7 | 0.76 | 52 | 140-160 |
| 502 | 17528 | F:AATCTACAGCAAGCGGTGGT R:AACCCACACGTTTCCCTCTT | (GA)8 | 2 | 0.25 | 52 | 140-150 |
| 503 | 17529 | F:GCATGCTCCTAGGAAACCAC R:AGTTGCAGAGCGAAGGAAAG | (GA)7 | 3 | 0.38 | 52 | 140-150 |
| 504 | 17530 | F:CCATTGAATTGTCAGGATCAGA R:TGTTTTCTCTGTGAAAGTGTTGTT | (GA)7 | 3 | 0.54 | 52 | 140-150 |
| 505 | 17531 | F:TGCAGGGGTGTGTGTTACAT R:TGAACATGGTGAAATGGATTG | (GA)6 | 4 | 0.49 | 52 | 130-150 |
| 506 | 17532 | F:TTTTCCTTTTTCAGCCACCTT R:TTTCGGCCAAAATCTTAGTGA | (CT)6 | 3 | 0.41 | 52 | 140-160 |
| 507 | 17533 | F:ATGACGGGAAGATGGACGTA R:TTTTGTCGTGACATTAGTTTTCAA | (CT)6 | 5 | 0.54 | 52 | 140-150 |
| 508 | 17585 | F:AAAGCAGTGCATGCAACAAT R:CAACCACACTGCAATAACAGG | (AG)7 | 3 | 0.28 | 52 | 150-170 |
| 509 | 17588 | F:TGCTTGCAATGCTAATGGAC R:GCCACATGCACATGACACAT | (GT)6 | 3 | 0.53 | 52 | 140-160 |
| 510 | 17591 | F:CCAATGTCATGCTTCCATCA R:ATTGTGGCCTCCATTATCAG | (CA)7 | 4 | 0.48 | 52 | 140-160 |
| 511 | 17593 | F:CATCCTCCTCCTCCATACCA R:TCATCATCAATGCAAAGGACA | (AG)6 | 4 | 0.49 | 52 | 140-160 |
| 512 | 17595 | F:TGACGATGGATCGAGTGAAA R:AGGCTAGGGGCTATCCAAAG | (TC)7 | 3 | 0.48 | 52 | 140-160 |
| 513 | 17596 | F:TGTTTGCCTGTGTGTGTGTG R:CGCATGCAAAAACCATCGTA | (GT)7 | 2 | 0.38 | 52 | 125-150 |
| 514 | 17597 | F:AACTTTGGCATGCCTATTTCA R:GCGTGTGTGTGGTTATGTGTG | (CT)6 | 5 | 0.65 | 52 | 145-160 |
| 515 | 17598 | F:GCGCATGCTCATACACTCAT R:TGCGTGTGTCTCTACGTGTG | (AC)8 | 2 | 0.37 | 52 | 140-150 |
| 516 | 17600 | F:TGCAACCTTTGGGTTTATCTG R:CATGTTGTTTTTGTGGAGTTGA | (AC)6 | 3 | 0.49 | 52 | 140-160 |
| 517 | 17601 | F:TACCATGAAATCGCCTGTCA R:GATTGATCCTCAACCCAACAA | (AC)6 | 3 | 0.43 | 52 | 140-160 |
| 518 | 17604 | F:GGGGAGTTGGGACCACTAAT R:GTCCCACATCGCCTATGAAT | (GA)7 | 4 | 0.31 | 52 | 150-160 |
| 519 | 17605 | F:CGCCCTTCATCATCATCTTC R:AGAGTCGGTCCCTCCAACAT | (CT)7 | 4 | 0.25 | 52 | 150-170 |
| 520 | 17609 | F:TGTGTGGGATAGGTTGTTTAAGTTT R:TGCTAAACCATCAGAAGCACAT | (TG)10 | 4 | 0.61 | 52 | 150-175 |
| 521 | 17616 | F:CACGACAAAGCTGTTGCACT R:AGCCTTCTCCACAAGCAAAA | (AG)7 | 3 | 0.24 | 52 | 150-170 |
| 522 | 17617 | F:TGCACCTGTGCATGAGAAAG R:GTGCACGTGTGTGTGTGTGT | (AC)6 | 5 | 0.47 | 52 | 150-170 |
| 523 | 17618 | F:CTATGAAGTTCGGGCCAAAG R:CGCCCTAACACCCATCATAC | (AC)6 | 2 | 0.14 | 52 | 150-170 |
| 524 | 17619 | F:GCCGGGAAATTTTCAACAAC R:AAATCACCCAAAACGCAGAG | (TC)8 | 4 | 0.42 | 52 | 150-175 |
| 525 | 17625 | F:AGCCACCATCTTCATCTCAAA R:GATGGCCTGGTTCAGATAGG | (TC)6 | 5 | 0.64 | 52 | 140-160 |
| 526 | 17628 | F:GGTTTTGTTTGCCGTTGATT R:CCACCCCCAAACTTCCTTAT | (GA)8 | 3 | 0.57 | 52 | 150-170 |
| 527 | 17629 | F:GGGTTGGATTGAGCATTTGT R:AACCAACTTTGAAGGCAGTTATT | (GA)7 | 4 | 0.49 | 52 | 140-160 |
| 528 | 17631 | F:TGCTGTTCCTTCTGCAGCTA R:CATGCCCACCATTTGAGACT | (CT)7 | 3 | 0.31 | 52 | 150-160 |
| 529 | 17633 | F:TCCCAAACACCTCATAGTTCC R:TGTGGTTTGAACACCCTGAT | (AC)6 | 5 | 0.58 | 52 | 140-160 |
| 530 | 17634 | F:CCTTTGTGAGGGGGATACCT R:AGGCAAGCAACGTTGAAAAG | (TC)7 | 5 | 0.66 | 52 | 140-160 |
| 531 | 17636 | F:CCATCCTTTCCAGAGCTCAC R:CAACCTCACATTCCCCTCAT | (GA)7 | 3 | 0.2 | 52 | 150-170 |
| 532 | 17637 | F:AAGGGTTATTTGGCCTTCTGA R:GGACACACCGCTTAGGTAGG | (CT)8 | 2 | 0.32 | 52 | 150-160 |
| 533 | 17638 | F:GGGCAGAAAACGAGAGACAA R:TTCCGTTTTGGCATGTGTTA | (CA)6 | 5 | 0.38 | 52 | 140-160 |
| 534 | 17639 | F:TCATGTGAAAGTGACTGATATCTTGA R:TTCAGCTTCAACACTAACCAACA | (AG)7 | 4 | 0.38 | 52 | 150-170 |
| 535 | 17640 | F:CTAAGTGGGGAGCCAAAACA R:TTTTTCAGACAAAAGGAGGAGAA | (AG)6 | 3 | 0.15 | 52 | 150-170 |
| 536 | 17642 | F:TGGCTTATGGAGGCAATTCT R:ATTTGATTCCAAGGCACCTG | (AG)6 | 3 | 0.46 | 52 | 150-170 |
| 537 | 17643 | F:TGATGAACAGTGCCAAAAGAA R:TGTGTGCATTCTTCATTCACC | (AC)8 | 4 | 0.56 | 52 | 120-140 |
| 538 | 17646 | F:GGGCTTGCATGGGTAAATAA R:AGGGGGAGGCACAATAAAGT | (TC)6 | 2 | 0.14 | 52 | 150-170 |
| 539 | 17647 | F:GTATGCGTGTGTGCAGGTTC R:CACGCAAATGCAAAGGTACA | (GT)7 | 3 | 0.5 | 52 | 150-170 |
| 540 | 17650 | F:TTCCATACCTGAATCCAAACCT R:CTTTGAATAGTGCGCATGGT | (TC)6 | 3 | 0.21 | 52 | 150-160 |
| 541 | 17652 | F:CCGAAGGCAACCTCTAACAC R:ACCAAGCCCAGCAGAAGTAA | (GA)7 | 4 | 0.28 | 52 | 150-170 |
| 542 | 17654 | F:CCTCCATCAAGCAACAAATG R:TGGTGAGGGATTCAGGGTAG | (CT)6 | 2 | 0.37 | 52 | 150-170 |
| 543 | 17658 | F:TCCGTGCTTATTGTCACACC R:AAAGCTCCCTCTCTCCCTCT | (TG)8 | 3 | 0.24 | 52 | 150-170 |
| 544 | 17659 | F:TCATCTGGATATGCCTCAAAAA R:GTGCCAATGTGAGTGACCAG | (TG)6 | 3 | 0.28 | 52 | 150-170 |
| 545 | 17660 | F:TCCCAAAGACATGGAAGAGAA R:CAAAAACCTTTTCGAGATCACA | (TC)6 | 3 | 0.48 | 52 | 150-170 |
| 546 | 17663 | F:AAAAGGTGTGGGATGCAAAG R:GGGGAAGAAGAACCGTGATT | (CT)6 | 5 | 0.67 | 52 | 150-170 |
| 547 | 17664 | F:TGTATGCGTGCATGTGTTTG R:TGTCAGTGTTGAGCGATTTG | (TG)6 | 3 | 0.48 | 52 | 150-170 |
| 548 | 17665 | F:TCATCAAAAACCACCCATCA R:TGAGGGGAGTTGCAAGTTTT | (TC)6 | 3 | 0.24 | 52 | 150-170 |
| 549 | 17666 | F:GTGCATTGGCTCGTACTCAA R:TCCACAATATAGCCCAGACCA | (TC)6 | 4 | 0.42 | 52 | 150-170 |
| 550 | 17667 | F:AGTGGTTGGACTTATGGTCGT R:TCGCAAAGCCTCTACGAAGT | (GA)7 | 3 | 0.48 | 52 | 150-170 |
| 551 | 17671 | F:TCTTCCTACACGCACGTTCA R:AGCAATCCTCGCATTTTAGC | (AC)6 | 7 | 0.74 | 52 | 160-190 |
| 552 | 17673 | F:TCCCAGCCAGTCCTTAAATG R:CCTGCTTACCACCGTCTTCT | (TC)13 | 8 | 0.66 | 52 | 150-170 |
| 553 | 17677 | F:GGTTCGATGGAGAATGGTGT R:CCTGAATGTCGGTGCTAAGG | (CT)21 | 3 | 0.33 | 52 | 140-160 |
| 554 | 17678 | F:CCCGAGTGCTTGGAGACTTA R:GGAATGAATTTTCCGGTGTG | (CA)7 | 4 | 0.39 | 52 | 150-170 |
| 555 | 17679 | F:TGCACACATCATGCACACTT R:CGCATGCATACGTGTGAGTT | (CA)10 | 4 | 0.44 | 52 | 155-170 |
| 556 | 17682 | F:GGCGAGCTTAGCGAATTATG R:TGTGAGTGAGAGGGAAAATGAA | (TG)7 | 4 | 0.35 | 52 | 160-180 |
| 557 | 17684 | F:TGATTGAAGCTGATGGTGCT R:CAATTGCAGACCGCAGTTTA | (TC)6 | 4 | 0.6 | 52 | 150-170 |
| 558 | 17689 | F:TGTTCTAATTTTATTCGCATTGTTC R:CATTGGATGATGATGGTGGA | (TC)6 | 3 | 0.22 | 52 | 160-180 |
| 559 | 17690 | F:GCACAAAGGATAAGTACTCAGTGTAT R:GCAATGGAGGTAGGAGGTCA | (GT)8 | 2 | 0.14 | 52 | 160-180 |
| 560 | 17692 | F:TGAAATATTCAACGTGTTATAGAGAGA R:CCCTGTCTTCCAGCTCCATA | (CT)6 | 2 | 0.37 | 52 | 160-180 |
| 561 | 17697 | F:TGGTTCGAGGAACTTGAGAGA R:CTCATTTGATCATTGCAACACA | (AG)6 | 3 | 0.15 | 52 | 150-170 |
| 562 | 17702 | F:GCCGGAGATAGCCATGATAA R:CAAAATCACTCAGATTTCGATCC | (AG)8 | 3 | 0.29 | 52 | 160-180 |
| 563 | 17703 | F:TTGGAGATCCCCGTTAACAC R:AAAGGCCTCACGACAAACTG | (AC)15 | 6 | 0.39 | 52 | 160-170 |
| 564 | 17707 | F:GGGTGTGTGAAAAGCAATGA R:CCAAATCAAGGGTTGTGGTC | (GA)7 | 3 | 0.4 | 52 | 160-170 |
| 565 | 17708 | F:TTGGAAAATGAACTCCCAAAA R:CCAAACTTCAGGTTCGATGA | (AT)6 | 3 | 0.49 | 52 | 160-170 |
| 566 | 17711 | F:TGGATCTGAACCGAAGCTCT R:TGCAACCAAATTCTGTGTGT | (GA)8 | 6 | 0.66 | 52 | 155-180 |
| 567 | 17714 | F:CGCATAACCATTTAGCATAGCA R:GAAAAGGAATGGATGGAGTGA | (AG)8 | 4 | 0.53 | 52 | 160-170 |
| 568 | 17719 | F:AGAAAGCCCATGCTAGTGGA R:TTGAACGCGTTGGATATTGA | (GA)8 | 3 | 0.31 | 52 | 160-180 |
| 569 | 17720 | F:TGATGGTCATGGTGAGGTGT R:AAGGCACATTGTGGTTCTCC | (GA)10 | 4 | 0.64 | 52 | 160-180 |
| 570 | 17723 | F:TAAGCGTGGGAGCAAAAGTT R:ATGAGTCCTCATGCCCTTGT | (TG)7 | 2 | 0.14 | 52 | 160-170 |
| 571 | 17726 | F:AGCGCTGTGGAGAAGAAGAG R:CAGCTCATACGGATACCAAAAA | (GA)6 | 2 | 0.15 | 52 | 160-170 |
| 572 | 17728 | F:CTCACTTGCACTTGCCTCAG R:TCAAGAGTGGGGAGGTTTTG | (CT)6 | 2 | 0.14 | 52 | 160-170 |
| 573 | 17730 | F:GTGGAACCTGAACCAGGAGA R:AACCCAACTGCAGGAGCTAA | (AG)6 | 5 | 0.67 | 52 | 160-180 |
| 574 | 17734 | F:TTCAAAGTCCTCCAACCTAGTCA R:TGCAGACATGGGCTTGATAC | (GT)8 | 3 | 0.56 | 52 | 160-180 |
| 575 | 17739 | F:TGCCAATCTGTTGGAATGAA R:CAATTAAAATGTGAATGGAACAATG | (AG)6 | 4 | 0.44 | 52 | 150-170 |
| 576 | 17740 | F:CCATTGTGCATGGACACTTTT R:CTGAGCCTCGACATGAAACA | (TG)12 | 4 | 0.54 | 52 | 150-170 |
| 577 | 17741 | F:TTCCTTTACGGGGATGTGAG R:TCCACTGAAATGGGAAGGAG | (TC)7 | 5 | 0.47 | 52 | 160-175 |
| 578 | 17742 | F:GCGTCTGATCTCTGCCTCAT R:GCATCGAAAGCAAGTGGAGT | (TC)6 | 5 | 0.43 | 52 | 150-175 |
| 579 | 17743 | F:CACTTGGCAAGCATCAGAGA R:GGTTTGAACGGTAGGGATGA | (TC)6 | 4 | 0.48 | 52 | 160-180 |
| 580 | 17746 | F:TGAAGTGGCTGCTTCTTGTG R:TCAACAATTTAAAGCACTCACCAT | (GA)7 | 3 | 0.34 | 52 | 160-180 |
| 581 | 17747 | F:CGGGAGTGAGGAGTTTCTGA R:ATTCATGTCGCGCTACACAC | (GA)6 | 4 | 0.34 | 52 | 160-180 |
| 582 | 17748 | F:CCTTTGTCCTTTTCCCTCTTG R:AGGGGTCATCAACGTGATCT | (CT)8 | 2 | 0.34 | 52 | 160-180 |
| 583 | 17751 | F:TCCAAACTTCACAATCCTCTTC R:TGTTGACGTGTCAGTGTTGTG | (CA)8 | 7 | 0.73 | 52 | 150-180 |
| 584 | 17752 | F:CGGGGTGGAATCTGTGATAC R:TTCGTTGGGCCTCTCTAAAA | (AG)6 | 4 | 0.34 | 52 | 160-180 |
| 585 | 17753 | F:CAGAAGAAATCCCCCAAGGT R:CGGTTGTGTTCACCCTTTTC | (AC)7 | 3 | 0.16 | 52 | 120-130 |
| 586 | 17754 | F:AGCAACGGGCAACCTTATAG R:CCTTTTGTTTGGAAGCTCAA | (TA)6 | 3 | 0.38 | 52 | 160-180 |
| 587 | 17756 | F:TTCATGCAGTATGAGTATGAGAAAGA R:TGCGATAAAGCAAAAGAACAAA | (GT)7 | 4 | 0.33 | 52 | 160-180 |
| 588 | 17757 | F:GATGGTGGTTGGATTCAAATG R:GATGAACCGAATGGTGGAGT | (CT)6 | 3 | 0.32 | 52 | 160-180 |
| 589 | 17758 | F:CAACACGCATCACCCTTATG R:TTGGATTCCCCTTCTCTCCT | (CT)6 | 2 | 0.19 | 52 | 120-140 |
| 590 | 17761 | F:AGCAACGGCTCCTTATCAAA R:CCATCCACCTTTTTCCTTCA | (AG)6 | 2 | 0.37 | 52 | 150-170 |
| 591 | 17762 | F:CAGACACACAGACACACATACACC R:GGGGCCAGAAAGGGTAAGTA | (AC)7 | 3 | 0.27 | 52 | 150-170 |
| 592 | 17765 | F:CTCCCTCACCCACTGACACT R:AAGGTGATGCGAAGGTTTTG | (TC)6 | 3 | 0.32 | 52 | 170-190 |
| 593 | 17766 | F:TGAAATTGGCTAAACAACAAGGT R:GGCAAAATCTCTTCATTTTGTG | (TA)7 | 4 | 0.61 | 52 | 170-190 |
| 594 | 17767 | F:CCACAGTCCTCCCTGATTGT R:TGGAGGTTAACAATAAGTGAAATGAA | (GT)6 | 4 | 0.28 | 52 | 170-190 |
| 595 | 17768 | F:CGCGTTTAGTTTGTTCGTAGG R:AAACGATCTGATGGCTCACC | (GA)8 | 3 | 0.44 | 52 | 170-190 |
| 596 | 17769 | F:GCAATTGAGTGGAAGTGTGTG R:TTTCTGCATTTTTGCCATTG | (GA)6 | 4 | 0.48 | 52 | 170-190 |
| 597 | 17770 | F:CCAAATTAGATAACACTTGCACAT R:TAGGCAAATTCCTCGCAGAT | (CA)7 | 3 | 0.48 | 52 | 170-190 |
| 598 | 17771 | F:CCATTTCTTTCCACGACTGG R:GGATGGCGGAGGTATTTGTA | (CA)6 | 2 | 0.36 | 52 | 170-190 |
| 599 | 17773 | F:TTCCACACGAGGCTATTTTC R:TGCAAAAGCGACATCTTGAC | (AG)7 | 4 | 0.53 | 52 | 170-190 |
| 600 | 17774 | F:CTCCTCGACAACTCCACCAT R:CCCATGGAATTTGTGTTTCTG | (AC)7 | 3 | 0.34 | 52 | 170-190 |
| 601 | 17907 | F:AATGCATGACCACCACAATG R:CCTTAAGGTTTTGAATTAAGATGTG | (AG)10 | 5 | 0.53 | 52 | 170-190 |
| 602 | 17910 | F:CATGCCTGCTTCCTTCTGTT R:TTGCAATTTCAAGCCTTCAC | (TC)8 | 2 | 0.34 | 52 | 180-200 |
| 603 | 17915 | F:AACTGAAAAGTCTCACTTCAAGAGC R:TTTGTTTTCCAATGCCACAA | (CT)7 | 2 | 0.08 | 52 | 185-200 |
| 604 | 17916 | F:AGGTTGGCCGTGATATAGGA R:CAACCAAGTGGCATCATCAG | (AG)7 | 3 | 0.54 | 52 | 185-200 |
| 605 | 17917 | F:GCAAGCAAGCACTGTGTTAGA R:TGCATTTGGAGCTTCCTTCT | (AG)6 | 3 | 0.41 | 52 | 185-210 |
| 606 | 17943 | F:AATCCTAGTGCCGCCTTTTT R:CATGTTTCCCCTCAAGCTGT | (CT)6 | 4 | 0.65 | 52 | 190-200 |
| 607 | 17947 | F:AAGCCCATCGATACCTTTTG R:CCTTGACCCACCTACGTCCT | (AC)7 | 4 | 0.55 | 52 | 190-210 |
| 608 | 17949 | F:GGGGTGCGAAGTTAAATCCT R:TCGCAAGTCGGAGAGAAAAT | (TC)6 | 3 | 0.4 | 52 | 190-200 |
| 609 | 17950 | F:ATACATGGGGGACTGCATCT R:GGGACTTAGGCCTTTGCATTA | (TC)6 | 2 | 0.14 | 52 | 190-200 |
| 610 | 17951 | F:TTTGGAAATAGGTGTCATTCATAA R:GGTGAGCCTCATATCCAAACA | (TC)23 | 5 | 0.65 | 52 | 175-200 |
| 611 | 17952 | F:TCATCCAATATATCAAACATACCTCT R:TGCTCGATCGTGTTTTTCTG | (GT)7 | 3 | 0.27 | 52 | 190-210 |
| 612 | 17954 | F:GGGGAAGGAAATTCCAGCTA R:ATTGAATCGTGGGTGAGAGG | (CT)8 | 3 | 0.4 | 52 | 190-200 |
| 613 | 17955 | F:TGCCTTCCCAAAGCTAGAAA R:CGATTTTGTTTTTCAGAACTCA | (CT)7 | 3 | 0.34 | 52 | 180-200 |
| 614 | 17958 | F:ACTCGAGCGAGTGTGGCTAT R:TCTCAAACAAGAGGGGAAAAA | (AG)6 | 2 | 0.36 | 52 | 190-200 |
| 615 | 17959 | F:TGAGTGGTATGTCACCACCAG R:TTGGCAACCTAAAGTCATTGTG | (TG)7 | 2 | 0.14 | 52 | 190-200 |
| 616 | 17960 | F:TTTGATAGTTCACTGTAACCAAGATG R:AGCACGGTGACGACTCTTGT | (TG)6 | 4 | 0.53 | 52 | 180-200 |
| 617 | 17963 | F:GCGGAATTCGGTATGTCTTG R:TCCTCTTCTCAAAGCCAGGT | (TC)8 | 4 | 0.56 | 52 | 190-200 |
| 618 | 17964 | F:GGGTTGATAGTGGCCCATAA R:CTGCCACGATATGCCTTCTT | (TC)7 | 3 | 0.32 | 52 | 190-200 |
| 619 | 17965 | F:AAACCATCCCCACAAATTCA R:ACTACGGAACCATCGAGCAC | (AT)8 | 3 | 0.5 | 52 | 190-200 |
| 620 | 17966 | F:GAACTTGACACTGGCGAGAA R:ACGGCGCCACTATTGACTAC | (AT)6 | 5 | 0.63 | 52 | 190-200 |
| 621 | 17979 | F:GCTTTTAGCGTCGGTTTTTG R:ACAATTGCTCGGTGGATTTC | (GA)9 | 4 | 0.22 | 52 | 190-220 |
| 622 | 17980 | F:CAATTCACAACGTTCCACTCA R:TTTTCGTGAAATTGAAATGACC | (CT)9 | 7 | 0.7 | 52 | 190-210 |
| 623 | 17981 | F:TTAAAAATGCCCGGACAAAA R:ATGGCAATGCAGGAGTCTCT | (CT)16 | 4 | 0.45 | 52 | 190-210 |
| 624 | 17985 | F:AAACTCAAGCAAGTGTCTACAAAA R:TGAGAAAAATCAATTTGAAAATATGTC | (AC)10 | 4 | 0.49 | 52 | 180-200 |
| 625 | 17986 | F:CCATGGTGGACGTCATTGTA R:GGCCTTTCACCTCACAAGAG | (TG)6 | 2 | 0.08 | 52 | 180-200 |
| 626 | 17987 | F:TGCCACCGACCTAATTCCTA R:CCCGCTCATGAAAAACTAGC | (TC)6 | 2 | 0.29 | 52 | 190-210 |
| 627 | 17988 | F:GGACAATTTCTCCCCTGTGA R:CGCGTGTCCATAACTTCAAA | (GA)7 | 3 | 0.41 | 52 | 190-210 |
| 628 | 17989 | F:CAGAGCCGGAGTTCTGGATA R:TTTGGTTGACATTAGCACATGA | (GA)12 | 7 | 0.79 | 52 | 190-210 |
| 629 | 17990 | F:GGAAAATGTGCAAGTAAAGAGAAA R:AAGAAACCCCTTTCAGAGTGC | (GA)11 | 2 | 0.37 | 52 | 190-210 |
| 630 | 18008 | F:GAGGTTGAGCTCACTCTTCTTGA R:AGCCTAGCCAAATCCCAAAT | (GA)6 | 3 | 0.24 | 52 | 190-210 |
| 631 | 18010 | F:CGAGGCGATCAGCATATTGT R:CGATGGCGAAATTTTTCTGT | (AG)6 | 2 | 0.11 | 52 | 190-210 |
| 632 | 18011 | F:GACCAACGACTTGGACATCA R:GGTGAGTTCCTAAGATGAATCAGA | (AC)6 | 5 | 0.33 | 52 | 175-200 |
| 633 | 18013 | F:TCAATTCCGAACCACCTTTC R:CGGCAGAATTAGGGTTTTGA | (TC)9 | 5 | 0.68 | 52 | 180-210 |
| 634 | 18014 | F:GGAGGGGGAGGACAATAACT R:GCGGAGTGAGGTTGTAGAGG | (TC)9 | 5 | 0.64 | 52 | 190-210 |
| 635 | 18042 | F:TGCCATCCACTCAAAAGTTG R:GTGTGAGGGTGGAGAGTGGT | (CT)6 | 3 | 0.21 | 52 | 190-210 |
| 636 | 18043 | F:AACATCACGGGTGCATTTCT R:AGGTGGGAGATGTAAGAGAGAAG | (CT)19 | 2 | 0.26 | 52 | 180-200 |
| 637 | 18044 | F:AATGGATGGAGTTGGCAGTC R:TTTGGATGTGAAAGCGTGAG | (AG)6 | 2 | 0.14 | 52 | 190-210 |
| 638 | 18046 | F:TGTGGGTGTCTTTTATTTTTCA R:TTTTTCCCACACCCATTCAC | (TC)6 | 2 | 0.15 | 52 | 190-210 |
| 639 | 18049 | F:ACCCCTCTTTGCTAGGGTGA R:ACCACACATCTCGCACACAT | (GA)9 | 3 | 0.42 | 52 | 190-210 |
| 640 | 18052 | F:ATCGGATGAGCTTCCAAGAA R:GGGACACCGAGGGAGTACAT | (AG)8 | 2 | 0.08 | 52 | 190-210 |
| 641 | 18054 | F:GATACTTGCCACCGTGATCC R:GCAGGGATGGTGATGATTTT | (AC)9 | 6 | 0.74 | 52 | 190-210 |
| 642 | 18057 | F:TGGTGAACCAAATGCGATTA R:TTGTACCTGGTGGAAGCTAGG | (CA)8 | 3 | 0.41 | 52 | 190-210 |
| 643 | 18058 | F:AAGAAATGGGAAACGCATTG R:CCGCAAGAAAACAAAATTGA | (AG)8 | 2 | 0.35 | 52 | 190-200 |
| 644 | 18059 | F:TTGCAAAGGTGATTCCATGA R:TGCAAACATGAACAATGCTAA | (AG)6 | 2 | 0.08 | 52 | 190-210 |
| 645 | 18060 | F:CCCCAAGAGCACAAGAAGTC R:AGCTTTTGCAAGCAGAGAGG | (AG)6 | 3 | 0.59 | 52 | 190-210 |
| 646 | 18062 | F:AGTTGGGAAGTTTGCAGGTG R:TTCTTGCTATCAACTTGCATTGT | (AG)10 | 2 | 0.08 | 52 | 190-210 |
| 647 | 18135 | F:CTTCAACCAACTGCGAGTGA R:TCATTTGAGTTTTGCCATGTTC | (A)11 | 5 | 0.72 | 52 | 110-125 |
| 648 | 18137 | F:TCTCTAACACCTTAATTCCCATAATTT R:TGGACGAAGATGGATGTGTT | (A)10 | 2 | 0.3 | 52 | 110-125 |
| 649 | 18138 | F:AAAAAGTCTATCTCACATGTTTGTTG R:GTCTTTGGGGTTTGGGTTTT | (A)10 | 3 | 0.53 | 52 | 110-125 |
| 650 | 18140 | F:AATTTGAAGGCGGAAACAAA R:GAGCTAGGATTAAGGAACGTCTT | (A)10 | 2 | 0.34 | 52 | 110-120 |
| 651 | 18141 | F:GGTGTTTTATTGCGGAAAAGTT R:CATATACTCCAACCCCCACCT | (T)11 | 3 | 0.52 | 52 | 110-125 |
| 652 | 18143 | F:GAGGTACCACAAAGGGTGGA R:TCCCATTGCAAATGATCAAA | (T)10 | 2 | 0.14 | 52 | 120-130 |
| 653 | 18144 | F:GGGTCGATGTGGAAGATGAA R:AAAATGCCCACACGTCTCTC | (T)10 | 3 | 0.4 | 52 | 120-130 |
| 654 | 18145 | F:CTCAAGCTTTAAGGCGAGGA R:GAAGTCCCGATGCACTTTGT | (G)12 | 5 | 0.69 | 52 | 120-140 |
| 655 | 18146 | F:TGGCTAGCAAAAACAAAACTTG R:ACCCCTTCGGAAGAATTGAT | (G)10 | 3 | 0.55 | 52 | 120-130 |
| 656 | 18184 | F:TGCGTGGAGTAATCCTTCTACA R:TGCTGTTAATTTTCCCCATTTT | (A)10 | 3 | 0.48 | 52 | 120-140 |
| 657 | 18185 | F:CTGGTGGTATGACCCGAAGT R:GGTTTTCCCCTGCTCTAGGT | (A)10 | 4 | 0.48 | 52 | 120-140 |
| 658 | 18187 | F:CTTTAGTTTAATCCAAACCCTAAAAC R:TGCTCTTTTGCTGTTGCTGT | (A)10 | 3 | 0.27 | 52 | 120-130 |
| 659 | 18188 | F:GCCGTGATGAAAGTCAGTTG R:TCCAATTCCCATTCCATTTT | (A)10 | 2 | 0.34 | 52 | 120-130 |
| 660 | 18189 | F:CATGCACAATCGATTTAAAGAAA R:GGTTGACCATCGCAAAACAT | (A)10 | 3 | 0.38 | 52 | 120-130 |
| 661 | 18190 | F:TCGGTCAGAAAAGGGTATGG R:CCCAAAGACATCCGAATCAA | (A)10 | 2 | 0.37 | 52 | 120-130 |
| 662 | 18192 | F:GCCTCTTCTGCATTTCAGGT R:CTTCAAATGCTGCACCTTCA | (T)10 | 4 | 0.53 | 52 | 120-140 |
| 663 | 18193 | F:TCTGTCATATTGCGGTCAGAA R:CATCCAAAGATCACCGGAGT | (T)10 | 2 | 0.28 | 52 | 120-130 |
| 664 | 18194 | F:TGGTCCCTTCATACCAGGTG R:ACTCACCAGCACACGCATAC | (T)10 | 2 | 0.36 | 52 | 120-130 |
| 665 | 18231 | F:CCCTCATTCGTTGGATCAAT R:ACGGCAGGGACAACATACAT | (T)10 | 3 | 0.44 | 52 | 120-130 |
| 666 | 18233 | F:TGCCAAGGCAATAGGAAAAT R:GGGAGAAGAAGACACGGACA | (T)10 | 3 | 0.43 | 52 | 120-130 |
| 667 | 18235 | F:AATGCGTTCATGCAGTGCTA R:TGGGGAAACATGGTTATTTTG | (T)10 | 2 | 0.08 | 52 | 120-130 |
| 668 | 18236 | F:CGGACCTTCAAGTATGCAGTT R:CAGGCAATGAATGGAAACCT | (T)10 | 2 | 0.37 | 52 | 120-130 |
| 669 | 18237 | F:GGGATATGAGAAGGCGATACC R:TGGTTGTAGGATGTGGGATTT | (T)10 | 3 | 0.36 | 52 | 120-140 |
| 670 | 18238 | F:TCCTTGGATCTTTACCCTGTTC R:TCCAAATTGCATCCACTGAA | (A)11 | 3 | 0.43 | 52 | 120-150 |
| 671 | 18240 | F:TCTCGTACGAAAGAAAAAGAAAATG R:GTGCGTGAGAGTGGGAGAAG | (A)10 | 2 | 0.14 | 52 | 120-140 |
| 672 | 18242 | F:AAAGGGCAAGAAATGGTAGGA R:TTTCGCAGTTAGCGACAATG | (A)10 | 2 | 0.25 | 52 | 125-140 |
| 673 | 18255 | F:TGTCAAATCCAATAAAAACACACA R:TTTGTGCACACCGTCAATTT | (T)10 | 3 | 0.12 | 52 | 125-140 |
| 674 | 18256 | F:TGCTTTCTCTCGCGTAGGTT R:TGCTTTAAAAGGGGAGGAGA | (T)10 | 4 | 0.61 | 52 | 125-140 |
| 675 | 18259 | F:GAATTGTCCCTCATTATCACGTA R:TGTCCCCATTTGTCCATCTT | (T)10 | 2 | 0.14 | 52 | 125-140 |
| 676 | 18260 | F:AACCTTGAAATGGAGGTACATGA R:GACCATGATCGGATGTTGTG | (T)10 | 3 | 0.47 | 52 | 120-140 |
| 677 | 18262 | F:GGACGATAAGTACATGGGTATGA R:GACGGGGAAGATGCTGTCTA | (A)10 | 3 | 0.12 | 52 | 125-150 |
| 678 | 18263 | F:TTGAACTCCTCTCCACAAGATG R:CACGCCATGTCCAATTATCA | (A)10 | 3 | 0.53 | 52 | 120-140 |
| 679 | 18264 | F:ATGAGGGGAGAGGGAGAAAA R:GGGCACCAATAACCAATAGC | (T)10 | 6 | 0.55 | 52 | 120-140 |
| 680 | 18265 | F:TCAACTTCGCCTTGTCACAT R:GGGAAGGACGTTCAATCTCA | (T)10 | 3 | 0.3 | 52 | 125-140 |
| 681 | 18267 | F:AAATGAAAAAGGGAACAAAGGAG R:GACTCGGTCCTTGCATGTCT | (A)10 | 2 | 0.25 | 52 | 130-140 |
| 682 | 18268 | F:TTTTCGATTGGATGGGAAAG R:GCTCGACCATCACGTAACAA | (T)11 | 4 | 0.55 | 52 | 130-140 |
| 683 | 18269 | F:CAATCGCATTGCATGTATGT R:TAATTCCGGCGTTTGCTAAG | (T)10 | 2 | 0.3 | 52 | 130-140 |
| 684 | 18270 | F:TTCGACGACGATCTCCTTTT R:CGCTGGAGACCAGTGCTTAT | (T)10 | 4 | 0.52 | 52 | 130-140 |
| 685 | 18271 | F:ATGGTGACAAGTGGTGGTGA R:GAAACGAAGCTGGGAAAGAA | (A)10 | 4 | 0.48 | 52 | 130-150 |
| 686 | 18272 | F:CCCCAACATTTCTCTAGGTAACA R:TTCTTCGCAGCTCGGTAAGT | (A)10 | 2 | 0.33 | 52 | 130-150 |
| 687 | 18273 | F:ACTTTAGGAGGGCGAACGTC R:CCCACCTTATTTTGGCTCCT | (A)10 | 3 | 0.32 | 52 | 130-150 |
| 688 | 18274 | F:GAGCACACTCGTCCAACTGA R:CAGAAAATAAACCAAACACCGTCTA | (A)10 | 2 | 0.08 | 52 | 130-150 |
| 689 | 18315 | F:CCGACTCAATCGAAACCCTAT R:TATAAGGGGGTGGGCTTTTC | (C)10 | 5 | 0.68 | 52 | 125-150 |
| 690 | 18316 | F:AAGAAATTGGCAGCGACAGT R:AGTCCTTTTGGTCCCCTCAT | (A)12 | 3 | 0.59 | 52 | 130-150 |
| 691 | 18318 | F:TGAAAGAGGATCCCATTGAATA R:CACCCTATGTGCTTCCTGATG | (A)10 | 3 | 0.36 | 52 | 130-150 |
| 692 | 18319 | F:GAAATCATACTCCATTTCACATTTTT R:CCTCCCGATTTGTGAAAGAG | (A)10 | 3 | 0.38 | 52 | 130-140 |
| 693 | 18320 | F:AGAGCGATGACACGGTTTG R:CCGCTATGCTATTCGACACA | (A)10 | 2 | 0.14 | 52 | 130-140 |
| 694 | 18323 | F:CAGACAATGGCAATTATTTGGTAA R:CTGCTGTTGCTTCGATTTCA | (T)10 | 3 | 0.4 | 52 | 120-140 |
| 695 | 18324 | F:GCACCTTTGGTAAAAGCATTG R:GACTATCTGCTGCCCCTCAG | (T)10 | 2 | 0.37 | 52 | 130-140 |
| 696 | 18325 | F:TTCCAATGGCGAAAAGTTGT R:AACCAAAATCCCTTGCAAAA | (T)10 | 4 | 0.56 | 52 | 130-140 |
| 697 | 18339 | F:TGGTTGAACTGGAACGAGTG R:TGAAATTGCAATGTAAGCATGA | (T)10 | 3 | 0.36 | 52 | 135-160 |
| 698 | 18340 | F:GCAACCCCACTATTCACTTAGC R:TGGGTTTGTCTATGGGGTGT | (C)11 | 2 | 0.17 | 52 | 130-150 |
| 699 | 18341 | F:AACCAAACGTAAGCCTCAAG R:TGGCCTTTTAGTGACGGTCT | (A)11 | 3 | 0.54 | 52 | 130-150 |
| 700 | 18342 | F:CATGCAATTCCAATGCAAAG R:TGTAGAGCCGCAAGTGTGTC | (A)11 | 2 | 0.35 | 52 | 130-150 |
| 701 | 18343 | F:CTGCTTCCTCTCTCGGAAAA R:GCAGTAGCAAGGAACAATCTGA | (A)11 | 3 | 0.51 | 52 | 130-140 |
| 702 | 18344 | F:GATTTCCCCAGCTCATTTCA R:CGAGCTTTAATGGAAGGGTTC | (A)10 | 2 | 0.19 | 52 | 130-140 |
| 703 | 18345 | F:GGTCTTCATTCACTTAGGGTTCA R:TGACCTATTTAGCCAGAGAACACA | (A)10 | 3 | 0.46 | 52 | 130-140 |
| 704 | 18346 | F:AGTCCAAAAGTCGCCTAGCA R:TGATGCTTTTAGCCGGTTCT | (T)12 | 3 | 0.32 | 52 | 130-140 |
| 705 | 18347 | F:GGCGTGTTTAGCAGGTTTTC R:ACCAGACGCGGAACTTTAGA | (T)10 | 3 | 0.45 | 52 | 130-150 |
| 706 | 18349 | F:AAAAGCTCAAAGTCTCGGAATAA R:TCTCCTGAGAGCGGAAAATC | (T)10 | 4 | 0.68 | 52 | 130-150 |
| 707 | 18363 | F:CATGCATGGAGTTGGAAGAG R:GTCCCAAAATGCAGCCAATA | (T)10 | 3 | 0.41 | 52 | 135-150 |
| 708 | 18365 | F:CCTTGTTTGCTGAGCCTTTT R:TATGACGGAAGCCCACTAGG | (C)13 | 3 | 0.47 | 52 | 130-150 |
| 709 | 18367 | F:CGGCGATGCTACAGTACATTT R:TCGATCGATCCGTCCATTAT | (A)11 | 3 | 0.36 | 52 | 130-150 |
| 710 | 18368 | F:ATCGAATGGAAGCAAACTGG R:TGTGGAATGTTGACCGAAAA | (A)10 | 2 | 0.34 | 52 | 130-150 |
| 711 | 18369 | F:TCAAAATCTGTTTGCCTCACA R:GTGGCCTGACACTGACACC | (A)10 | 3 | 0.47 | 52 | 130-150 |
| 712 | 18370 | F:CGACAAGGCAAGAGAGAACC R:AAGCGCAATGACAACAACAT | (T)10 | 4 | 0.51 | 52 | 130-150 |
| 713 | 18387 | F:TCGGCATGATGAACATATGAA R:GCCAATCAACGTGTTTCTTC | (T)10 | 6 | 0.68 | 52 | 140-160 |
| 714 | 18391 | F:CCATCCTCCACGTGTCTCTT R:TCGCATATCCAAATGCAAAC | (T)11 | 4 | 0.51 | 52 | 140-160 |
| 715 | 18392 | F:TCATCAGATGCATGATAAGCAC R:CGCTTGGGATCTTCCATTTA | (T)11 | 4 | 0.53 | 52 | 140-160 |
| 716 | 18394 | F:TGCTGAAGGGAACATGGAGT R:GCGCCGCTACTGATAACTCT | (T)10 | 2 | 0.37 | 52 | 140-160 |
| 717 | 18396 | F:AATGGGTGAATGGTCCTCAC R:GCGGTTACCGAGGAGGAT | (T)10 | 2 | 0.14 | 52 | 140-160 |
| 718 | 18397 | F:CACAAGTCTCAACAAAGCAACA R:GCTTCTTCGCCACTTTCTTG | (A)13 | 4 | 0.52 | 52 | 140-160 |
| 719 | 18435 | F:GGTGAGAGGTGATTGTGAAAAA R:CATCCACCCTCCGGTTACTA | (T)10 | 2 | 0.34 | 52 | 180-200 |
| 720 | 18438 | F:GATTGAGCCGTGCCAATATC R:GATCCCACCCTAGAGGAAAAA | (T)10 | 4 | 0.52 | 52 | 140-170 |
| 721 | 18439 | F:ACGCCTTCGTTGTAGCTTTG R:CAACCAGATCGGAACTCCAC | (T)10 | 4 | 0.34 | 52 | 130-160 |
| 722 | 18440 | F:TTGCAGGAGAAAACGTTGAG R:GGAGGCCACATACAGCAGTT | (T)10 | 2 | 0.14 | 52 | 140-160 |
| 723 | 18441 | F:AGCAGAAAGGAGACGCAGAA R:TTATGGCCGCATCAATCATA | (A)12 | 3 | 0.43 | 52 | 140-160 |
| 724 | 18442 | F:CTTGTGTGCCTCGAGTGTGT R:GCTGTGGAATCAGCCTTTTG | (A)10 | 2 | 0.11 | 52 | 140-160 |
| 725 | 18443 | F:TCCCTAACTCAAACCCTAACTCA R:CCAATTTATGGCGAAGCACT | (A)10 | 4 | 0.41 | 52 | 140-160 |
| 726 | 18444 | F:CGATTTGGGTGTTCTAGGTG R:CCTGACGATCTTCCCATCAT | (A)10 | 3 | 0.47 | 52 | 140-160 |
| 727 | 18445 | F:CATCGCTGTGCACTACAACA R:GTGGTTGTGTTTGCGTTCAA | (A)10 | 3 | 0.48 | 52 | 140-160 |
| 728 | 18447 | F:CGGCAAGTGACTCAGATGAA R:AACCCCCTTCAAAGCTGAAT | (A)10 | 4 | 0.52 | 52 | 140-155 |
| 729 | 18450 | F:GCCTGTAGGCCCAACAAGTA R:CACAAAGGGCTATTGCATGA | (T)10 | 2 | 0.37 | 52 | 140-155 |
| 730 | 18453 | F:GTCTCTGTAGACGCGGTGGT R:AACGGCTTGGCTTACAACAT | (T)10 | 4 | 0.6 | 52 | 130-150 |
| 731 | 18454 | F:TCACAATGGATTCCTCTCTCTG R:CCGGTAATGCTTGCTATCGT | (T)10 | 2 | 0.14 | 52 | 140-155 |
| 732 | 18455 | F:GCATGGTGGAACCTCTGTCT R:AAGCCCAAATGACACCAAAG | (T)10 | 4 | 0.61 | 52 | 130-150 |
| 733 | 18456 | F:TCCAGTCAGGGGCTTAGAAA R:ACCAGGCAAAAATTGAGGTG | (G)10 | 5 | 0.64 | 52 | 130-150 |
| 734 | 18457 | F:AAAGTGCAAGCCAACGTTCT R:CTTAGTGTTGCCGTCGGTCT | (A)12 | 3 | 0.29 | 52 | 130-150 |
| 735 | 18458 | F:CATTTCCCTCTCCCCTTCAC R:CCCCTTATTGCAAACGAACT | (A)11 | 2 | 0.35 | 52 | 130-150 |
| 736 | 18459 | F:CACAAAGACCCGACCAAATC R:CTGACCAGCTGGACCCTTTA | (A)10 | 4 | 0.46 | 52 | 140-160 |
| 737 | 18460 | F:AAAGGAATAGAAGGCTAGGGAAAA R:GGGGACCTGGCTTTAATTTC | (A)10 | 5 | 0.66 | 52 | 140-160 |
| 738 | 18470 | F:CTCCTACTCGGGTTCCCATA R:CTCGAGTTTTGAGAAGCAGTCA | (T)10 | 2 | 0.33 | 52 | 140-155 |
| 739 | 18475 | F:GCCCACAATTGCACAAACTA R:TTGCCGAGTCACCTCTTTCT | (A)10 | 3 | 0.43 | 52 | 145-160 |
| 740 | 18481 | F:CTCATCCAACCACCAGGAGT R:ACGTGCTAGCATTGGCTTGA | (T)10 | 2 | 0.14 | 52 | 145-155 |
| 741 | 18490 | F:GCGTGAAGCTGCAAGGTAAT R:CGGGCTAAAAGGAATCGTAA | (A)10 | 2 | 0.35 | 52 | 140-160 |
| 742 | 18493 | F:TTTTCTTCTCGTGGCAGGAT R:CTTCGAATGCCTGGTTCAAT | (T)10 | 5 | 0.53 | 52 | 140-160 |
| 743 | 18494 | F:CCTATTTGTCGGGCTCAGAT R:TAGAGCCCAGAGACGTCCAC | (C)11 | 4 | 0.31 | 52 | 140-160 |
| 744 | 18495 | F:TGAAAATGCACAATGCAACT R:CGTGTTGACGTGTGAGTCCT | (A)11 | 4 | 0.68 | 52 | 140-160 |
| 745 | 18496 | F:CCGATGGGCTACTTTTGTTC R:TGGAGCTGCTGGTGTTGTAG | (A)10 | 4 | 0.52 | 52 | 140-160 |
| 746 | 18498 | F:GCTTTTTCAATGATCCCTGTTT R:TGTGGTGCCTTTGTGTTGAT | (A)10 | 4 | 0.33 | 52 | 140-160 |
| 747 | 18507 | F:TGGCCAGGTCTAGGTTCTTG R:CAAAACTGCACTGGCTTTGA | (T)10 | 3 | 0.27 | 52 | 150-160 |
| 748 | 18508 | F:CTGCAGGATATGTTGCTGGT R:GGCAAAGCACAAATTAAGCAC | (G)11 | 4 | 0.48 | 52 | 150-160 |
| 749 | 18509 | F:AATGCGTGAGCCTTCCTTTA R:GACTCAGATCGAGGTGGACA | (C)10 | 4 | 0.39 | 52 | 150-160 |
| 750 | 18510 | F:TGCATTTCAAAATTGGTGGA R:TCCCCTGCTGTGACTGTGTA | (A)11 | 3 | 0.55 | 52 | 140-160 |
| 751 | 18511 | F:TTCTTGGTTAGAGAAGGCATACA R:AACAACTCTCAATAGATAAGGTCAAGA | (A)10 | 3 | 0.21 | 52 | 150-160 |
| 752 | 18512 | F:ACGCCTCATTCGTTTTTGAG R:GGCGAAATTGAGGAGGTTTA | (A)10 | 3 | 0.5 | 52 | 140-160 |
| 753 | 18513 | F:GCAACCATGGATGGTGAATA R:ATTCGGGGTTTCATCCTACC | (A)10 | 4 | 0.38 | 52 | 140-160 |
| 754 | 18515 | F:GGGTGAATGGGGAAAATATG R:AAGGTCATTCCCCAACTGTG | (A)10 | 3 | 0.44 | 52 | 150-165 |
| 755 | 18516 | F:TGATGTGCAACCAGCACTCT R:TTTCCCTAGCTGATGGTGGA | (A)10 | 5 | 0.62 | 52 | 150-160 |
| 756 | 18517 | F:GAAAGACTTCCGGACCACAA R:GAAAGACTTCCGGAACACACA | (A)10 | 3 | 0.33 | 52 | 140-150 |
| 757 | 18519 | F:ACCTGCAGCAAACTTGTCCT R:GCAATGCTACTGGCATCTCA | (A)10 | 3 | 0.28 | 52 | 145-170 |
| 758 | 18521 | F:GCTGAATGGGCAGAGTTGTT R:ACCGTTTCGACCTGATATGC | (T)11 | 5 | 0.51 | 52 | 150-160 |
| 759 | 18522 | F:TCCAAACTCGGCTCTGTTCT R:ATCCCTCTGAACCGAAGCTC | (T)10 | 3 | 0.44 | 52 | 150-165 |
| 760 | 18524 | F:AATTCTTTGACGAGGGAGGA R:GCCGTTATAGAAGACGCAAGTT | (T)10 | 4 | 0.52 | 52 | 150-160 |
| 761 | 18525 | F:AGAAATGCTTGCCAAGTGCT R:TCCAGTCTGATTCTTTCGTCTG | (T)10 | 2 | 0.37 | 52 | 150-160 |
| 762 | 18528 | F:ATGCCATGAAGCAAGTTTGA R:CAGAGTGGCAGAGACAGCAC | (T)10 | 3 | 0.27 | 52 | 150-160 |
| 763 | 18529 | F:GAATGTGCGTCCAACATCCT R:AGATTTTGATGCGGAAGAGC | (T)10 | 3 | 0.52 | 52 | 150-160 |
| 764 | 18533 | F:TCCAAAATGCGTGTCATCAT R:TGACCGACACATTCATCTTCA | (A)10 | 3 | 0.5 | 52 | 150-160 |
| 765 | 18534 | F:AACCATCTCAGAAAAACCATGAA R:TGCTCCTAAAATCATTGAGGTGT | (A)10 | 3 | 0.39 | 52 | 150-160 |
| 766 | 18536 | F:TTTCGTTAAGTGGTGCCTACA R:TGAGCTCCGACAAACACAAG | (A)10 | 4 | 0.54 | 52 | 150-160 |
| 767 | 18537 | F:TCCTCAAGAAGAATATGGTTCCTAC R:AAAGGGTCATTCCTTTGGAGA | (A)10 | 2 | 0.37 | 52 | 150-160 |
| 768 | 18538 | F:ATCGCACTTCATTCCCTCTG R:TCACCCCCATCAGCTTTATC | (A)10 | 3 | 0.29 | 52 | 150-160 |
| 769 | 18541 | F:GCCTTCAGTATCGAGGAGGTT R:GCGAAAATGTTCCTGGATGA | (T)10 | 3 | 0.38 | 52 | 150-170 |
| 770 | 18542 | F:TGAAAATCAGGGTTTCTTCTTTG R:TCTCCTCCAAACGACCAGAT | (T)10 | 3 | 0.5 | 52 | 150-175 |
| 771 | 18561 | F:TCGAATCACCCCAAGAAGTC R:GCAGGGGGTTTTATGACAAT | (A)10 | 2 | 0.24 | 52 | 150-160 |
| 772 | 18562 | F:TTCTTCTGCTGCTGCTCAAA R:AAAACAAAAACCACAACCAAAAA | (T)11 | 3 | 0.46 | 52 | 140-160 |
| 773 | 18564 | F:ACGAAATGATGTCCCCGTTA R:CGTTTTCGCGATTTTCATTT | (T)10 | 6 | 0.49 | 52 | 150-160 |
| 774 | 18566 | F:AAATCGGGAATGCCATGTT R:ATCCTCGCACGAATCTTCAC | (T)10 | 2 | 0.11 | 52 | 150-160 |
| 775 | 18591 | F:AGGGCCGAATGCTAAGTGAT R:TTTTGAACCCTGGAGGGAGT | (A)10 | 2 | 0.26 | 52 | 155-170 |
| 776 | 18592 | F:CCTCCTAAATAAAAATGTTTACGAAAT R:CCGAAAATCACCTCCTCAAA | (A)10 | 2 | 0.14 | 52 | 150-160 |
| 777 | 18593 | F:TTTGTCCTAGCTTGCGTCCT R:CGTTGATTTCAAAACTCGCATA | (A)10 | 2 | 0.19 | 52 | 150-160 |
| 778 | 18595 | F:ACTTCCACCAGCAACCAAGA R:TTGTGTGAATGCTCGGTTTT | (A)10 | 2 | 0.37 | 52 | 150-160 |
| 779 | 18597 | F:AAGGGAAGCACGTTGATGAC R:CTTGTCGGGATTTTCCTCAG | (T)11 | 3 | 0.43 | 52 | 150-160 |
| 780 | 18598 | F:TCCGCAAACTAGTGGTCTCC R:GACGAACTTCCAGGGTCAAA | (T)11 | 3 | 0.15 | 52 | 150-165 |
| 781 | 18599 | F:TCTGTTATGGCGCTTTCCTC R:CACTGTGGGGCCCTAGTAAA | (T)10 | 3 | 0.15 | 52 | 155-170 |
| 782 | 18600 | F:CCTTACCAGGTGACGACCAT R:TCTTTATGCGAAGCACAACAA | (T)10 | 6 | 0.68 | 52 | 150-165 |
| 783 | 18639 | F:GGACAAACAACTTCAAGCAAAA R:TACGGGGGTGGAATGTAAAA | (A)10 | 2 | 0.14 | 52 | 160-175 |
| 784 | 18644 | F:GTGCATGTGTGCTCGATTTC R:TCGACTTGCAACTAGAAAAATCA | (T)10 | 2 | 0.34 | 52 | 140-160 |
| 785 | 18645 | F:GCCATCATGTTTAGGCCTGT R:ATGCAAAGGAGTCAGCAGGT | (T)10 | 4 | 0.43 | 52 | 150-165 |
| 786 | 18647 | F:TGTGTTGCGTGACTTTTGTG R:TTTTTGGTGTGATGATGGATGT | (T)10 | 2 | 0.3 | 52 | 155-165 |
| 787 | 18650 | F:AATTGGGCATGCGATTTTAG R:GCCCAAATGTTGTAGCCAGT | (T)10 | 3 | 0.4 | 52 | 155-165 |
| 788 | 18699 | F:GAACCGATCCAAGCAAAGAA R:ATCCACCATCGCTTCCATTA | (A)10 | 3 | 0.21 | 52 | 160-180 |
| 789 | 18700 | F:AAGGGACTAAATTGGACCTTGA R:ACTTGTGGAGGCATTCACCT | (A)10 | 4 | 0.36 | 52 | 160-180 |
| 790 | 18701 | F:TGCAACCACAAAATCAGGAC R:CCATGTCATATGGGACGAATA | (T)10 | 3 | 0.15 | 52 | 160-180 |
| 791 | 18702 | F:TGAATTTACCACTACAATATCCAATCA R:GCGTACGAGGGAGAGAAAGA | (T)10 | 3 | 0.55 | 52 | 160-180 |
| 792 | 18706 | F:GCAACCGGATCTTGTGAGAG R:CCACACCACACCTTAATCTTATCA | (A)12 | 4 | 0.61 | 52 | 160-180 |
| 793 | 18708 | F:CTCCATAGCGGAAGCTCACT R:TGATTTGGATTTGCGATAAGG | (A)10 | 5 | 0.49 | 52 | 160-180 |
| 794 | 18710 | F:GCGCAGAGTGTGTGAGTGTT R:TGGTCAACTTTCCCGAAAAC | (A)10 | 4 | 0.28 | 52 | 90-110 |
| 795 | 18724 | F:CATCATTTTCCCATGGACCT R:GCCATGTGTTTTCTATGGGACT | (C)12 | 3 | 0.47 | 52 | 160-180 |
| 796 | 18725 | F:TTCCCTTCTCAACCATCCAC R:AGAAGGAAGGCGTACCATTG | (C)10 | 2 | 0.36 | 52 | 160-180 |
| 797 | 18726 | F:TATGAAGTGCCCCGAGTTTT R:TGATGTTGTGAGCTCATGTTGA | (A)11 | 2 | 0.28 | 52 | 160-180 |
| 798 | 18728 | F:TGGCTGTCACCAGCTCATAG R:AGTGGGTCTCGTGGTGAGAG | (A)11 | 2 | 0.28 | 52 | 110-130 |
| 799 | 18729 | F:AAGGTCCCATGTCCCAAAAT R:ATGGTGGTTGGAGGAATGAA | (A)11 | 4 | 0.6 | 52 | 160-180 |
| 800 | 18732 | F:TGAAACTTATTCTGAGATTGCAAAAA R:AATGCGTGTCGTCTGACTTG | (A)10 | 2 | 0.37 | 52 | 160-170 |
| 801 | 18733 | F:CCAGCAAGAACGTCCTATCA R:ATTCTTGCCTTTGGGCTTTT | (A)10 | 2 | 0.37 | 52 | 160-170 |
| 802 | 18748 | F:TCGAGACTCCGCGAGTAGAT R:TATCCCCATTTTTCGAGGTG | (A)10 | 7 | 0.79 | 52 | 160-180 |
| 803 | 18749 | F:TCAAACACTTCATGTGTAAGATGTAAA R:CGGTGTGGTGATGACAAAAG | (A)10 | 2 | 0.08 | 52 | 165-180 |
| 804 | 18771 | F:CCAACAATAAAACCCCAAACA R:AGCGAGGTGCGTTCACTATT | (A)10 | 2 | 0.14 | 52 | 160-170 |
| 805 | 18773 | F:CCCAATTCCCTTCACTACCA R:TCCATGAAAATGCAATAGAAAAGA | (T)10 | 3 | 0.15 | 52 | 160-170 |
| 806 | 18777 | F:CCTACGGTAGCGGTGACATT R:AGTGGTTGGGGATCCATTTT | (C)10 | 5 | 0.45 | 52 | 160-180 |
| 807 | 18778 | F:TAGCCGGAAAAAGCAAAGAA R:CTTGGGCTGGCCCTAGTAGT | (A)11 | 3 | 0.45 | 52 | 160-180 |
| 808 | 18779 | F:CACCATCTCCATCTGCATCA R:TGTTGATTTCATACTGAACTATTGATT | (A)11 | 3 | 0.3 | 52 | 160-175 |
| 809 | 18781 | F:CAGTCACACCCTCAAAAGTGAA R:AGCCTTCTCCAGCAAAGACA | (A)10 | 4 | 0.54 | 52 | 160-175 |
| 810 | 18784 | F:GAATGAGGGGTGGACGAGTA R:TTCGGTTCATTCTCGCTTCT | (A)10 | 5 | 0.57 | 52 | 160-180 |
| 811 | 18786 | F:GCCTGCCTTCATTCTTCAAC R:CACAACCAGTGGTGGAGCTA | (T)11 | 4 | 0.43 | 52 | 160-175 |
| 812 | 18788 | F:CGCATGACCAACAATCAGGT R:GGGTGCTCCATTCTCCTAAA | (T)10 | 2 | 0.08 | 52 | 160-180 |
| 813 | 18789 | F:CTTGACAAGACCCTAAAAATCAA R:GCCATTGATGTGCTTTCAGA | (T)10 | 3 | 0.18 | 52 | 170-180 |
| 814 | 18794 | F:CCCCCAAAAGAATCAGTCCT R:TGGTGTTCTTATTTGGGAAAAA | (G)11 | 2 | 0.14 | 52 | 130-140 |
| 815 | 18905 | F:TCAAAATGTGTCGGTCTCTGA R:GAAAAACGCCTTTGCTTTTG | (T)10 | 3 | 0.43 | 52 | 170-190 |
| 816 | 18910 | F:GTAGGGGTACGGGGGTAAAG R:TTCAGATTTGCAGAGGGAAAA | (A)10 | 3 | 0.46 | 52 | 170-200 |
| 817 | 18928 | F:TGAATGTGGAAAGGAGGAATG R:AGGGTCACCACTTTGGAGAG | (T)10 | 3 | 0.41 | 52 | 170-180 |
| 818 | 18931 | F:TGCGATGCATAGTAGGTGGA R:CCAATGTCTTCCTCTTGCTTTT | (A)11 | 2 | 0.33 | 52 | 170-180 |
| 819 | 18933 | F:GGAATCAGGAATGTGTTTGTTG R:CACGAAATTGCGAAAACCTT | (A)11 | 4 | 0.55 | 52 | 150-180 |
| 820 | 18934 | F:TTGCAAAGACCTGTACACAAAAA R:CCAGAATTCAAACGCCTTGT | (A)11 | 4 | 0.65 | 52 | 170-190 |
| 821 | 18936 | F:CCTCTCTCACGCATTATTTCCTA R:GCGAAATCCCTCACCTTACA | (A)10 | 3 | 0.42 | 52 | 170-190 |
| 822 | 18937 | F:CCAGTGAAACAGGATTTGAGGT R:GAGTTTTGCAAGAAGCAACAA | (A)10 | 2 | 0.14 | 52 | 170-190 |
| 823 | 18938 | F:GACCTGCAGGACAAGGCTAC R:TCTCTTCAATTGGGAGGTCTT | (A)10 | 3 | 0.57 | 52 | 170-190 |
| 824 | 19073 | F:CATGATCACGGGTCTCTTCA R:TCGGTGTCTCTCGAAACAATC | (T)10 | 4 | 0.57 | 52 | 180-210 |
| 825 | 19075 | F:CACGAGTACAACATGGAGTGAAG R:CAAGCTCAACCTCCTCATACC | (A)13 | 2 | 0.37 | 52 | 185-200 |
| 826 | 19080 | F:CCCTCAAAATCCATCGCATA R:TGGCAGTACTTGCACTTTCTT | (A)10 | 2 | 0.26 | 52 | 180-210 |
| 827 | 19143 | F:GCCCCTTTTCATGTTGTTTG R:CAGCAGAAGAAAAATTGCAAAA | (T)10 | 2 | 0.32 | 52 | 190-210 |
| 828 | 19144 | F:TGCATTCAAACCAAGCAAAA R:ATGGTGGAAGATTTGGTGCT | (A)12 | 3 | 0.38 | 52 | 190-210 |
| 829 | 19145 | F:TCAACTTCATCCTTAGACCAGTCA R:CAATAGGCCACCAAGCCTTA | (A)11 | 2 | 0.14 | 52 | 180-200 |
| 830 | 19202 | F:ATTCACCGCCTATGTGCTCT R:TCCCAATCCACCAATATCGT | (C)15 | 3 | 0.47 | 52 | 190-210 |
| 831 | 19254 | F:AAATCCGAAAAATGCATTATCTTTA R:TCCTCGACGTCATCTCCTCT | (A)10 | 2 | 0.35 | 52 | 190-200 |
| 832 | 19255 | F:CACCAACACCTGCAAAATGA R:TCTAGCGTTGGCATCAAGTG | (A)10 | 2 | 0.33 | 52 | 190-210 |
| 833 | 19256 | F:TGCCACAACCAGCTATACGA R:TTGCAAGGATTAAGGCTCTTC | (A)10 | 4 | 0.37 | 52 | 190-210 |
| 834 | 19316 | F:AAAGCCTTTCGCAATCTACG R:TGTGCCATGTTATTTTTCACAA | (T)10 | 2 | 0.14 | 52 | 120-130 |
| 835 | 19319 | F:CTTGTAGGCTGGAATGTCTCAA R:TCATCTCAGCTGGGACACAT | (T)10 | 2 | 0.37 | 52 | 170-200 |
| 836 | 19320 | F:TTTGAGGCTAGTCTCTAGAGGTATGA R:CTTGTTTCCTCCCCTGACAA | (A)10 | 2 | 0.14 | 52 | 120-130 |
| 837 | 19343 | F:TCCCCTTTGATTCTCCTTTG R:GGTGGGTGTGAATGTTGATG | (T)10 | 3 | 0.57 | 52 | 200-210 |
| 838 | 19344 | F:GCGGAACATGTGACAACGTA R:TTCAATGGTGTTCCTGTGGA | (T)10 | 4 | 0.45 | 52 | 190-210 |
| 839 | 19349 | F:TTGCCTGACAAGGACATTTG R:CCCCAAATGTCAAAATGTGT | (A)11 | 2 | 0.37 | 52 | 170-190 |
| 840 | 19353 | F:TTCATGCTAAAACAAATTCCAAAA R:GCTTTAAATCGCCTCACACC | (A)10 | 3 | 0.45 | 52 | 200-210 |
| 841 | 19358 | F:CGATCAAACCAACTCATATACACC R:GGAAGGGTGTAAGCCATTGA | (A)10 | 3 | 0.15 | 52 | 200-210 |

Note: F= Forward primer; R= Reverse primer, PIC= Polymorphism Information Content, *T*_a_= Annealing temperature
