# Supplementary material for: High-Throughput Development of SSR Markers from Pea (Pisum sativum L.) Based on Next Generation Sequencing of a Purified Chinese Commercial Variety
Source: PLoS One. 2015 Oct 6;10(10):e0139775. doi: 10.1371/journal.pone.0139775 (PMC4595016; doi:10.1371/journal.pone.0139775)
Supplement: S3 File — (DOCX) [file pone.0139775.s003.docx]

**S3: Supplementary Table 2 Monomorphic SSR markers of *Pisum sativum* L.**

| **No.** | **Marker Name** | **Primer sequences(5’-3’)** | **Repeat motif** | ***T*_a_(℃)** | **Expect Size(bp)** |
| --- | --- | --- | --- | --- | --- |
| 1 | 16143 | F:TTGAAATGAAAGTACCCCAGTT R:CCACACCATCCCATCCTACT | (TGA)6(GA)6 | 52 | 115 |
| 2 | 16156 | F:CCAAACGGAAGACCAAAGAA R:AGGAGCAGGTGAGAGCAGAG | (CT)8catacat(AC)6 | 52 | 131 |
| 3 | 16158 | F:CAACCGAGGCCGAGTATATTT R:GGTGGTGTGGTTATGGAGGA | (CT)6ca(CT)15 | 52 | 132 |
| 4 | 16160 | F:AGCGGCAGACCTAATCTTCA R:ACGATTCGTGAGGAAGATCC | (CT)7gaaaacgtgaag(A)10 | 52 | 137 |
| 5 | 16161 | F:GGTGTTGGCACACTCACTTC R:CAGCCCGAACAAAATCCTAA | (CT)7caac(CT)10 | 52 | 137 |
| 6 | 16164 | F:CACACACACCCACACACACA R:CAACTGGCTATGCTCCAAAA | (CA)6cc(CA)6 | 52 | 141 |
| 7 | 16166 | F:ACAACCACCGCCTCAGTATC R:CGTCGGGATTAGATCAAACTG | (GCA)5(ACA)5 | 52 | 142 |
| 8 | 16167 | F:GTGCTTGCGTGCGTGTAA R:CACGTGCACACACACACC | (TG)7tttgtgtgc(GT)7 | 52 | 143 |
| 9 | 16171 | F:TCCCTTCCCCACACTTAAAA R:TGCGATCACCTATCCACCTT | (GA)7ggg(GA)15 | 52 | 153 |
| 10 | 16173 | F:GTGCCGCAACTAAAGTGTGA R:TGTTGTTTATGGGTCGGTGTT | (CT)10cc(CT)7 | 52 | 153 |
| 11 | 16174 | F:TGAACAAGATGGACACAAAACA R:TGCATGAGCTTTTCCATTCT | (AGA)5ataagaat(AAG)10 | 52 | 153 |
| 12 | 16176 | F:GAATACGCCACACCCATTTT R:CACGCGCATATGTTGAAAAC | (GT)6(G)12 | 52 | 155 |
| 13 | 16182 | F:CGTGTGCGTGTGTGTGTATG R:CGCACCAATGATTTTTCTTT | (TG)13agac(AG)9 | 52 | 158 |
| 14 | 16190 | F:GCACACGCACACACATACAC R:CTCGTTTGCCACCCCTAC | (CA)6aacacatatgcacg(CA)8t(AC)8 | 52 | 168 |
| 15 | 16197 | F:CCGAAGCTTTGAACCTATGC R:GCCTCCTGAATGAACCTGAG | (A)10g(A)10 | 52 | 177 |
| 16 | 16200 | F:TGAGGCTGTTAGGCATGTTG R:GGGAGAGGCTCATGTTGTGT | (TGT)6(TGA)5 | 52 | 180 |
| 17 | 16203 | F:GGGACGAGTGTGAATGGACT R:GCAAACTCAAACTCTACTTGCATTC | (GTG)5(GCG)5 | 52 | 182 |
| 18 | 16207 | F:CGCTTGAGGCAGATGGAATA R:GCTTACCGAAACCCCTTACC | (GT)6a(TG)9(AG)9 | 52 | 185 |
| 19 | 16209 | F:ATGCACGCACACGTACTCTC R:AACATCTCTCGACCCGTGAC | (AC)9gtacgcgcgcgcg(CA)10 | 52 | 185 |
| 20 | 16212 | F:CTGCCAACAACCACAACAAC R:GGGTCGCAACTCCCTTATTT | (CAA)6catcagcaaccatagtat(CAA)5 | 52 | 187 |
| 21 | 16217 | F:GCATTGATGGGATGAATATGG R:CGCATATTGCAATTGGTTTG | (A)10tcat(TG)6 | 52 | 195 |
| 22 | 16218 | F:CCAGCTCTCACGTGAATTGAA R:TGCAATACCGTGTTTTCATCA | (T)10ctgacatactaat(TC)12 | 52 | 196 |
| 23 | 16219 | F:ATCCGGCATTCTCTGTCTTG R:TCTAGGGTACCCCGAAGGAG | (TGT)5gtcggtcctcttg(GTT)5 | 52 | 198 |
| 24 | 16220 | F:GCGAAATCTCTACTTCCAGGT R:TTTTTCACACGAATTCACCAT | (TCT)5ctcttcga(TCT)5 | 52 | 198 |
| 25 | 16221 | F:GGAAATTCACACGCATACACA R:GAATGGATTTCTTCAATCTCAGTG | (CA)9cgtgcgcgtgt(AC)6 | 52 | 198 |
| 26 | 16231 | F:CACGTGGCAGATTATGCTTTT R:GCAAGAACTGAGATCTGGATGA | (CT)10cg(CT)6 | 52 | 209 |
| 27 | 16232 | F:CGCTTGATTGTGTCCATTTG R:CATGTGTGCGTGTGTGTGTG | (CA)8tacg(CA)12 | 52 | 209 |
| 28 | 16233 | F:GTGCACGGATTCATGTGTCT R:ACGCATACACGAATGCACAC | (TG)7tttctgtc(TG)7cata(TG)9 | 52 | 210 |
| 29 | 16254 | F:AAAGCGCTGTCTAAGGCATATC R:TGAGCAATAACCGCCTTTTC | (AAAT)5 | 52 | 150 |
| 30 | 16256 | F:TGCAATAAATGCCTCTAAGTTCC R:ACCCATGACCTCATGCTCTT | (AATG)5 | 52 | 165 |
| 31 | 16261 | F:TCCCATAGAAAAAGATGTATACCAAAT R:CCAGAAGGATCATTGGAGGA | (ATTA)5 | 52 | 194 |
| 32 | 16263 | F:TTTACCCCCAAATCAACAGC R:CATTCAGGTCCACCAAAGGT | (ATAA)5 | 52 | 203 |
| 33 | 16266 | F:CTGTTGCTGCTGACGATGTT R:GGAGGAAAGATGTGCGATCA | (TGA)5 | 52 | 110 |
| 34 | 16269 | F:GCGGTTGAAGATGAAAGCTC R:TTGACCCTCTTGGGATCAAC | (GCG)5 | 52 | 110 |
| 35 | 16277 | F:AGCAGCGGAAATCATCAAGT R:TAGCACTGGAGATGGGGTCT | (CAA)5 | 52 | 111 |
| 36 | 16280 | F:GGCTGATAGGCCTACTGTTGT R:GTCCTGCCGTTAACCAACAC | (TGT)6 | 52 | 112 |
| 37 | 16282 | F:GCAACATTGGCCTATTCCTC R:AGAAGGGGCTTATGGCTACG | (TCT)16 | 52 | 112 |
| 38 | 16285 | F:CGTCTTTGCGCTTCAACTT R:CAACGATTGGTCGAGGATTT | (CAA)5 | 52 | 112 |
| 39 | 16287 | F:TCAAAAGCAAGGTCCCATTC R:ATCGGCCTATTTGGGTTCTT | (CAA)5 | 52 | 112 |
| 40 | 16289 | F:AACCAGTCATGTTCGCATCA R:AAAGGATATGCGTATGCGAGTT | (AAT)7 | 52 | 112 |
| 41 | 16290 | F:ATCAGCAACGGGTCAAAATG R:CCAACAACTCAGCCTCAACA | (TTG)5 | 52 | 113 |
| 42 | 16304 | F:ATGCATCTTCATGGGCTGAT R:TCTCTGGCTTGTCCTCACTG | (GAT)5 | 52 | 114 |
| 43 | 16305 | F:ACGCATATGAAGCCGACCTA R:CGCCATCTTCAATGTTGTTG | (GAA)5 | 52 | 114 |
| 44 | 16307 | F:TGAGCTAAACCACTGCATTCTC R:CGGAAAACTTCAGCAAGTGA | (AGA)5 | 52 | 114 |
| 45 | 16308 | F:TGATTTCTCACCCCTTTGTTG R:AACAACCTCATGCCATCCTC | (TTG)5 | 52 | 115 |
| 46 | 16313 | F:ATATGGCTGGCGGTGAAAC R:CGGGTGGTTGTGATGAAATC | (CAC)6 | 52 | 115 |
| 47 | 16314 | F:AGTCGTTGCTGCAAGAGGTT R:AATGGGTGTCCCTGTGAATG | (AGC)5 | 52 | 115 |
| 48 | 16315 | F:TTTTGCTTGAGCTTAACCACTG R:AAACTTCCGCAGGTGAGTTC | (AAG)5 | 52 | 115 |
| 49 | 16316 | F:TCGAGGTTGGTAGGCTTGTT R:CTGTCGTCAACCAACACCAG | (TGT)5 | 52 | 116 |
| 50 | 16317 | F:AGGCCCTTCATCTTCATCCT R:TAGAGACTTGCCCCGATGTT | (TCT)5 | 52 | 116 |
| 51 | 16319 | F:CAACGGTTGTTGGTTTCCTC R:CGCAGACACCATCCATCATA | (CTC)5 | 52 | 116 |
| 52 | 16321 | F:CAACTCAACAAATCAATCAGTTCC R:GGTCAAAAGATACCTTCTTCCTTTC | (CAA)5 | 52 | 116 |
| 53 | 16326 | F:ATGCGATTGAAGGGATGAAC R:TCCTCCACCATATCCACCAC | (AGG)5 | 52 | 116 |
| 54 | 16330 | F:GCAGAGTTGGTAGTGGCAATTAT R:GAATGCACCAGAAAGTTGTAAGG | (TCA)5 | 52 | 117 |
| 55 | 16331 | F:AAATTCTTCTTGCTGCCAATTC R:TCTCCAAATGCCATGAATGA | (TCA)5 | 52 | 117 |
| 56 | 16332 | F:TCCAGCTGTTGTTGCTGAGT R:TCAGCTTAGTTGCATTGTTTTG | (GTT)5 | 52 | 117 |
| 57 | 16335 | F:CGCAGTGATCAGAGCAAGAG R:TGTTGTTGTTGTTGAATTAGAATTATG | (CAA)5 | 52 | 117 |
| 58 | 16342 | F:CCATGGATGCTTCATGTGAG R:GCTCAAATTGCGAAATTCTTC | (TGA)5 | 52 | 118 |
| 59 | 16343 | F:CGAGGTCAAGATTGGTGATG R:TTCAAAGGTCAACGATTCTCTTC | (GTT)5 | 52 | 118 |
| 60 | 16344 | F:CCCATGCATGACTTTTTGTG R:TGTATTTGGACCTTACAATCTTGA | (ATG)5 | 52 | 118 |
| 61 | 16346 | F:GCAGCTGCTCCACCATATTA R:TCCAGTTAGCGCTCCAAAGT | (TCA)5 | 52 | 119 |
| 62 | 16348 | F:CTCCTGGACTCGGAAAAGAA R:CACACTCCTGTGGCTTCGTA | (GAA)5 | 52 | 119 |
| 63 | 16354 | F:GTCCTGCCATCAACCAACAC R:ATTATTGTTGCGAGGCTGGT | (CAA)5 | 52 | 119 |
| 64 | 16355 | F:TCAGTTCCTATTCAGCAACAACA R:GGAATCGGATCAAAAGAGACC | (ACA)5 | 52 | 119 |
| 65 | 16360 | F:TGATGTGGGCTGCTAACTTTC R:GGAATGCTCCACAACAAGGT | (TGT)5 | 52 | 120 |
| 66 | 16361 | F:GATCCGGCATCCTCTGTCTA R:ATCGTCAAGTCGCTGTTGTG | (TGT)5 | 52 | 120 |
| 67 | 16362 | F:TCCCCTCACCATTGTTATCC R:ACGACCAAGATGAGGAGGTG | (TCA)5 | 52 | 120 |
| 68 | 16367 | F:GCATCAAGAGAAATAATAACAATATGC R:AGGTCATGCAAAGAAAACCAC | (TTG)5 | 52 | 121 |
| 69 | 16370 | F:GCCGGTAGGCCTGTTTTTAT R:GCCGTTAACCAACACCAAGT | (TGT)5 | 52 | 121 |
| 70 | 16371 | F:GGCACTTGCGTCTTTTGAGT R:GCTTGGACTGACTCCCAGAT | (TCT)5 | 52 | 121 |
| 71 | 16374 | F:CTGCCACCATAACCACAAAA R:CAGAGTCGATGTGGCATTTG | (CAC)6 | 52 | 121 |
| 72 | 16376 | F:TCCAGCAATTCCAACAATCA R:CCTTCTTCCTCTCAAAGTCCTGT | (CAA)6 | 52 | 121 |
| 73 | 16379 | F:TCTCTCTGCTCATCCCCTCT R:GGTTGAGATCGGAGCAACAT | (AAG)5 | 52 | 121 |
| 74 | 16380 | F:GCAAAGGGTCGAAACGTCTA R:ACCTCAACCTCGTCAACAGC | (TTG)5 | 52 | 122 |
| 75 | 16385 | F:CGTAGAGGGGGATGTGGTAA R:CAACCTCATGGTTTCCAGGT | (CAA)5 | 52 | 122 |
| 76 | 16387 | F:GCGGAGATTGGAAATGAAGA R:GACCCATGCTGGGGATAATA | (AAT)5 | 52 | 122 |
| 77 | 16389 | F:TCCCAGCAGTCATCTGGTAA R:CATGACGACGACGAAGAAGA | (TTG)5 | 52 | 123 |
| 78 | 16394 | F:AATGCAGCTTCCAATGAACC R:GAGTCTTTGGATGTGTGTAAATCAA | (CTT)5 | 52 | 123 |
| 79 | 16398 | F:AACAACCGCAACAAAGAACC R:CCAAAGACGGGTACAACTCTACA | (ACA)5 | 52 | 123 |
| 80 | 16399 | F:AGTTGCCATCAGAGCAATGTT R:CAAAAGGCAAGAAGGGTTGA | (TTC)5 | 52 | 124 |
| 81 | 16400 | F:ATATGACCTTGGCCCGTATG R:GGTCCCAAAACCTGAAACAA | (TGC)5 | 52 | 124 |
| 82 | 16411 | F:GCAGTTGCGCAGTTGAATAA R:TGAATTGGGGGAGAAAGAGA | (TTC)9 | 52 | 125 |
| 83 | 16413 | F:GAGCCAGATTCAAAACCAGAA R:GGTGCATTTGTCATCCCATA | (CCA)5 | 52 | 125 |
| 84 | 16417 | F:GAGCTAAGCCACTGCATCCT R:CGGAGAAGATGATCGGAAAA | (AGA)6 | 52 | 125 |
| 85 | 16418 | F:TTGGCCTTCTTCCTCTCAAA R:CCCCAACAATTCCAACAATC | (TTG)5 | 52 | 126 |
| 86 | 16420 | F:CATGCAGTGAACTCATTCTCA R:GAAAAATGGAAGAAAGCTTGTGA | (TTC)5 | 52 | 126 |
| 87 | 16421 | F:GATGCTCGTGTTGCTTTGAA R:CCCCTTTGCATCCTCTACAC | (TGT)5 | 52 | 126 |
| 88 | 16425 | F:ACCGGAGAAGGAACTGAACC R:AGCTGTTTTCTCGGCTTCAA | (GTT)5 | 52 | 126 |
| 89 | 16426 | F:GATAGTGGTGGCGGTTCTTG R:TCACCTCTCTCAAAGCCTCTTC | (GAA)5 | 52 | 126 |
| 90 | 16427 | F:CGGAGAAGATGATCGGAGAA R:AGCCACCTCACTCTCACTCTG | (CTT)5 | 52 | 126 |
| 91 | 16430 | F:TCAAGCCCCCAACATTTAAG R:CGAGTTGTTGTGGTTGGAATTA | (ACA)5 | 52 | 126 |
| 92 | 16432 | F:CGAATGGAGTACGTTGGTTGT R:TGAGAAAAAGAAGAGAAAACGTC | (GGC)5 | 52 | 127 |
| 93 | 16440 | F:TCCTTGCCATGATTTTCTCC R:GCGGTAAGACTCGTCATTCA | (TCT)5 | 52 | 128 |
| 94 | 16441 | F:TCCTCCAAAATGGCTCTCAC R:TTCTTTGTTGCGGTGGATTT | (TCC)5 | 52 | 128 |
| 95 | 16451 | F:AAAGGTCTCCGACTTTTGACC R:TGTTGTGTTGTGTCGTGTTG | (AAC)5 | 52 | 128 |
| 96 | 16455 | F:CTGTGACCATTCCGACTGC R:CCATCACCTTTTGCCTCTGT | (ACA)5 | 52 | 129 |
| 97 | 16457 | F:GCCAAGTCCGTGTTCTTTGT R:CGAATCCAGGCTCAGATTTC | (TTG)5 | 52 | 130 |
| 98 | 16472 | F:GGTGGAATGGGTGAAGAATG R:TCCCACAGAAACACCCATCT | (TGT)5 | 52 | 132 |
| 99 | 16477 | F:TCTTCAGTTATTCCTGTGTTTTCA R:TCGTTGTTGATGATTGTTGGTA | (CAA)5 | 52 | 132 |
| 100 | 16478 | F:CCAGTGGTGAATCCGGTTAG R:GGAGCAGTTCTCCACCACAT | (ATG)5 | 52 | 132 |
| 101 | 16479 | F:GAGGACCAAAGACGGGTACA R:GCAACAACAACTGCAACCAT | (TTG)5 | 52 | 133 |
| 102 | 16480 | F:GGCACCATTTCCATCAGTCT R:GGGATAAGTGCTCCCGCTAT | (TCT)6 | 52 | 133 |
| 103 | 16481 | F:TGGTTGTTGACGTGGTTGAT R:CATCATCCGAACCAGGAATC | (GTT)6 | 52 | 133 |
| 104 | 16484 | F:AGTGACCATTCCTGCACCTC R:CAGTGGGTCGAACCTTCTGT | (AAC)5 | 52 | 133 |
| 105 | 16486 | F:CTCATGGGTGTGCAGAAAGA R:GAGGGGTTGTTGGACTTGAA | (TCC)7 | 52 | 134 |
| 106 | 16489 | F:CAGGATGCGACACTCCACTA R:TGTCACAACCTCACGCCTTA | (GAA)5 | 52 | 134 |
| 107 | 16490 | F:GATCGTGCCAATGTTCAAGA R:TATTGGATGCATACGGGACA | (CAA)5 | 52 | 134 |
| 108 | 16494 | F:CGGGCCGAGTTATTTATCAG R:CCGAAATAAACGGAGCTGAA | (TGA)6 | 52 | 135 |
| 109 | 16495 | F:CTCTCCAAAGGGGGAATGTT R:TCGCATATGCCATAAATCACA | (TAA)6 | 52 | 135 |
| 110 | 16496 | F:GGCAGAAGCCTGTGAAAGTC R:ATCCCCAACCACAAACTCAA | (GTG)5 | 52 | 135 |
| 111 | 16498 | F:CCATCAATCTCCTCACACGTA R:GCTTTGATCTGATTTTCCAATTC | (AGA)5 | 52 | 135 |
| 112 | 16500 | F:CGCTGACACCAGAAGAACTG R:ATTCTGGCCAACAAGGACAG | (TTC)5 | 52 | 136 |
| 113 | 16503 | F:GGTCTTCATCTTGATTGGAATG R:CTGGCCCAATGTACCTGATT | (TCA)5 | 52 | 136 |
| 114 | 16506 | F:ATCCATCAAGTGCATGTCCA R:CCTTTCACTAACGGCACACA | (GTG)5 | 52 | 136 |
| 115 | 16516 | F:CCACGGTTGCTTTCTTCCTA R:GGGTGTGAGTGTGAGGGAGT | (GAA)5 | 52 | 137 |
| 116 | 16518 | F:TCTTTTGACGCTACTTTTATGTCA R:AACAATAATAAACATGCATGAGGAG | (CAT)7 | 52 | 137 |
| 117 | 16521 | F:TTCCCCATGCATAAAACACA R:AGAGGACAGACGCACCAGAT | (TTA)7 | 52 | 138 |
| 118 | 16530 | F:CCCACCATTCCAATTTATGC R:CCGGTTGGTGAAGGTAAGAA | (TTC)5 | 52 | 139 |
| 119 | 16533 | F:CGGGGTCTTCACATCCTCTA R:TTCAGTACCAACCGCAACAA | (GTT)5 | 52 | 139 |
| 120 | 16539 | F:AGAAGCTGCTCCACCACATT R:GAAGTGCCTGCAGTTGATCC | (TCA)6 | 52 | 140 |
| 121 | 16545 | F:AAGGCCCTCTATGAGGAGGA R:CCTGGTAGGCCTGTTGTTGT | (CAA)6 | 52 | 140 |
| 122 | 16546 | F:GCAGGTAATGACGACCAAGA R:TTGTTCCCATCCCAATTGTT | (AGA)5 | 52 | 140 |
| 123 | 16547 | F:CCACATAGCAAAAACGCTAGG R:CAGCGTAGCGTATGGAATCA | (ACA)5 | 52 | 140 |
| 124 | 16553 | F:TGGTTGGAAGAATGCAATGA R:TTGAGCTTCATCTTGGTTGAAA | (ATG)5 | 52 | 141 |
| 125 | 16557 | F:CACAACCAATGCTTCAGGTG R:GGCAGCCTGATGGTAAGGTA | (GGA)5 | 52 | 142 |
| 126 | 16560 | F:CTGGTTTCAGCTTGGACTGG R:GAAGAAGTTCGAGGCACTGG | (AGA)5 | 52 | 142 |
| 127 | 16564 | F:CCCCTGTTAACATTGGAAAAA R:TTTGCCTTTTTGTTGTGTATGG | (TCA)5 | 52 | 143 |
| 128 | 16573 | F:CCCTTTGCTTAATGTTGTCCA R:TCCTTACAAATGTTAAGGGTGAAA | (TTG)6 | 52 | 144 |
| 129 | 16576 | F:AAGACTTTGTGCAACCCAAAA R:TCCCCCTCCTTCTTCTTCTC | (GAA)6 | 52 | 144 |
| 130 | 16584 | F:TCCAACGATTCCATCAATCA R:GGTCAAAGGAGACTTTCTTCCTC | (CAA)6 | 52 | 145 |
| 131 | 16586 | F:TTGGGAAAGTCTGAAAAGACTAGAA R:CTCTCGGCTGCTTTTGTCTC | (AGA)6 | 52 | 145 |
| 132 | 16591 | F:TTCCGTTATTCCGGTATTTTC R:AGTTCTGCTGCTGCTGATGA | (CAA)5 | 52 | 146 |
| 133 | 16594 | F:GCACGTCCATTGTGTTTGTC R:GAGATCTGTTGTCTTCTCCACTGA | (TCA)5 | 52 | 147 |
| 134 | 16598 | F:CAATTCCGGTGAAGCTCAGT R:GGGGCAGTTGTTATGCTTGT | (CAT)6 | 52 | 147 |
| 135 | 16599 | F:TTTCACGAGGAGCATCATCA R:GCAACTACTGGAAACCGAAAT | (CAT)5 | 52 | 147 |
| 136 | 16601 | F:GGACATTATCGCCAGGTTGT R:AAAGCAACGATCCGACATTC | (ACA)6 | 52 | 147 |
| 137 | 16602 | F:TACAGCCCCACAGGCTAATC R:ATGCAGGATGCTGTGAATGT | (ACA)5 | 52 | 147 |
| 138 | 16605 | F:GAGGCACATGTTGCTTCTGA R:TTTGGATTCACTTTGGACTGG | (TCT)10 | 52 | 148 |
| 139 | 16606 | F:CAATGGTGGATCCCTTCAAC R:CTGAGAGGCACCTCGTCTTT | (GAA)5 | 52 | 148 |
| 140 | 16618 | F:AGTGAGGCTGGAACTGAGGA R:CCAGACTCCTTCTCCTCCAC | (GGA)6 | 52 | 149 |
| 141 | 16620 | F:TGAAGTCAACGTCAAAGCATAA R:GTTGAGGCGGACCCTATACA | (GAA)5 | 52 | 149 |
| 142 | 16632 | F:CACCCTGTCTGAAAAGGTTTG R:CGTACCTAGCCAAGGGTATGA | (A)10 | 52 | 110 |
| 143 | 16635 | F:GCTCCTCCTGAGCCACATTA R:CCGATATGATTCGGGTGTTA | (A)10 | 52 | 110 |
| 144 | 16638 | F:GTCTTCCGGTTTGCATTGTT R:TTTGAGTAGTTGAAAAAGCACCAG | (T)10 | 52 | 111 |
| 145 | 16640 | F:TGGCAACAGTTGTGCAATTT R:GTAGTGGGTCGAATGCAGGT | (T)10 | 52 | 111 |
| 146 | 16641 | F:TCATCATTGTGGCACCTCTT R:TCATCTAGTAGCGACGAAGCTATG | (T)10 | 52 | 111 |
| 147 | 16651 | F:GGGATTTGTGGCAGGTTTAC R:GCTTGGAAGTGGCAAGAAGT | (T)10 | 52 | 112 |
| 148 | 16659 | F:ATGTGCCCCATTACCAATGT R:TATTCAGGGCCGAGATAACG | (T)10 | 52 | 113 |
| 149 | 16665 | F:GCGCTTGCAGTAACGAACTA R:TTTCTCATTCGAGCAGAACTGT | (A)11 | 52 | 113 |
| 150 | 16667 | F:CAATGGGAACGCTCTCTCTT R:CGTTCTTTCTGCAACGTGAT | (A)10 | 52 | 113 |
| 151 | 16678 | F:GCCAAGCAGGAACCATGTAG R:TCCCTTGTTGGGTGTTTTTC | (A)10 | 52 | 114 |
| 152 | 16682 | F:TTGTGTTCCTGGCTTCAAGA R:TGGTTTCCACCGTCTTCTTC | (A)10 | 52 | 114 |
| 153 | 16686 | F:TTTGGAAGATGAGGTGAGTTTG R:TTCGACCCAAACAAAAACCT | (T)10 | 52 | 115 |
| 154 | 16689 | F:AACAAAACAAAGCTCACAGTGC R:TCAATCACTGGGGTTGATGA | (A)10 | 52 | 115 |
| 155 | 16690 | F:TCCATCGAGACACTCATTTTTG R:TCCAGTGAATTCAGATGCAGT | (T)13 | 52 | 116 |
| 156 | 16700 | F:GGTTGACTAGAGTTAGCTGAGTTATGG R:TTTTCCGAGTTCGAGCTTCA | (A)10 | 52 | 116 |
| 157 | 16711 | F:TGCATAGTGCAGAAGAAAAGGTT R:CGTGTCAGGTCCCTGTTTTT | (A)10 | 52 | 117 |
| 158 | 16714 | F:GTAGAAGACGAAGCGGGTCA R:TGGAACACACTAATCATCGGTAA | (T)10 | 52 | 118 |
| 159 | 16715 | F:CCCGTGTCCTTGAGGAGTTA R:TTTGGTGAAATTCGGAGGTG | (T)10 | 52 | 118 |
| 160 | 16716 | F:GACGATGGCAAAGTTGTGTC R:GAACCATGCAGGTCCACATT | (T)10 | 52 | 118 |
| 161 | 16755 | F:TGTGCGTGATTTTAGTGATGTATT R:CACACACATTACACACACATCAGA | (TG)6 | 52 | 115 |
| 162 | 16762 | F:ACAAAGGCTCAAGGGTGATG R:GCCATTTTAATGGTTTTTCCAC | (AC)8 | 52 | 115 |
| 163 | 16763 | F:AAGCTTCGGAAGACCATCTTT R:TGTCAATGCTTTGATTGTTTGA | (GA)7 | 52 | 116 |
| 164 | 16765 | F:GGGCGCCACTGTATTGTCTA R:CTAGCCTCGCCCTATCTGTG | (TG)9 | 52 | 117 |
| 165 | 16766 | F:TTCAGGATGGTGGCCTTATG R:TCACCACATCAATGGCCTAA | (TG)7 | 52 | 117 |
| 166 | 16882 | F:CCCAGTGGGAAACCATTTTA R:AAGCATGCAATATCCTACGAAAA | (CT)8 | 52 | 133 |
| 167 | 16884 | F:ACGCGCACACATACACAAA R:TACTTGCATGCGCGTGTGT | (CA)9 | 52 | 133 |
| 168 | 16885 | F:CGCACACACGAAAACACAAT R:TGTGTGTGTATGTGCATGTATGT | (CA)6 | 52 | 133 |
| 169 | 16886 | F:GCACGCACACTCACACATTA R:TGTGCGTGTGTTTGTCTGT | (CA)6 | 52 | 133 |
| 170 | 16889 | F:CCCTTGTGCAAAAACACTTG R:TGTGGAGAAAGGCCCAAATA | (TC)8 | 52 | 134 |
| 171 | 16894 | F:GGGGGATGGACCTAAAGTTAC R:TTCTGCAACAATGAGTGAATGA | (TC)6 | 52 | 135 |
| 172 | 16895 | F:TTCTGACAGCTCCCAATTCA R:TCGTACAAGTATGGGGCTAAA | (GT)8 | 52 | 135 |
| 173 | 16897 | F:CCCCGCAGAGTTGATAATGT R:TCAAAGGAGGGAAGCATCTG | (CT)6 | 52 | 135 |
| 174 | 16900 | F:AACGGAAACAAGCGTAGGTG R:AGTGTGCACGACCGTAACAA | (AG)7 | 52 | 135 |
| 175 | 16906 | F:TTCTTGAGCCCCATAACCTG R:TTCAGAGGGAGGGAGTTTTC | (TC)6 | 52 | 137 |
| 176 | 16909 | F:CATACATGCGCATCATGACA R:ACCCATCCGACCTGATATTG | (CA)7 | 52 | 137 |
| 177 | 16913 | F:ATGATTCAAGCACCCCAAAA R:GCACAAATTCAATGCGGTAA | (CAA)5 | 52 | 149 |
| 178 | 16915 | F:CACCCCAATTTCAATCCTACA R:CTGGTGATCGCTGAAGATGA | (AGA)5 | 52 | 149 |
| 179 | 16918 | F:CACCTCGAGCATGAGAACAG R:TGAAGTAATCCCTGAGATGACAA | (CTC)5 | 52 | 150 |
| 180 | 16921 | F:CTGGGTTGTCTCGGTTGATT R:ACTCTAAGAGGGGTCGAGGA | (TTG)5 | 52 | 151 |
| 181 | 16922 | F:CATGCGAACTTTTCGACAGA R:ATTTGTGGCCGGAGATGAT | (TTC)5 | 52 | 151 |
| 182 | 17055 | F:GGACGGTCAACGTCAAAACT R:AACCCAATTGTTGCAAGTCTG | (ATG)5 | 52 | 164 |
| 183 | 17061 | F:AAGCTGGCAGCACTGAATCT R:ACGGGAAAGCGTGTATTTGT | (AAG)5 | 52 | 165 |
| 184 | 17062 | F:GGGGCAAGATGAGTTGAAGA R:TGGGGACTACCTCCCTCTTT | (AAG)5 | 52 | 165 |
| 185 | 17067 | F:TCGGAGTGTTGTCCGTGTTA R:CCGACTCAAGAGAGTTCCAAAA | (TGA)7 | 52 | 166 |
| 186 | 17069 | F:GCTACATGGCAATGCAACAG R:TGCTGGTTCAAATGGTGTGT | (TCA)5 | 52 | 166 |
| 187 | 17072 | F:TTCCCAACCAATTTCCGATA R:GGTGGAGCAGGTGAAATTGT | (CAA)5 | 52 | 166 |
| 188 | 17075 | F:CCAATGGTGGATCCTTTCAA R:TAACTTTCTGGCGCTCACTG | (AGA)5 | 52 | 166 |
| 189 | 17078 | F:TTTCCAAAGAAAACACCTTCCTA R:GAGGAAAAGGGGTGAAGGAG | (AAC)5 | 52 | 166 |
| 190 | 17082 | F:AATTTGCAACCCCGTAAACA R:TCAAAGTTCTGTTGTTGTTGATGA | (ACA)6 | 52 | 167 |
| 191 | 17084 | F:GGTGGAGGGTGTAGGTTCAG R:AAGCTGGGGGAAACTTTTGT | (GAG)5 | 52 | 168 |
| 192 | 17086 | F:CAATGGAAGGAGGAGGAATG R:GGAGACTGTTGGCTGCTGAT | (ATC)5 | 52 | 168 |
| 193 | 17091 | F:CACCACAACCAACTGGAAAA R:ATAATAGGGGCCACCTCCAC | (AAG)7 | 52 | 168 |
| 194 | 17092 | F:AGCGCTCACTGAGAGGTACA R:TGGAATTGATCATTGGTGGA | (TTC)6 | 52 | 169 |
| 195 | 17094 | F:AACTGGGGGAAAATTGCTCT R:GATGGGGAGGATGCTTATGA | (TGG)6 | 52 | 169 |
| 196 | 17095 | F:AGCAGAGTCAGCAGGGTCAT R:GATGGTGGGTTTTTGGTGAC | (TGG)5 | 52 | 169 |
| 197 | 17096 | F:GGTGGTAAGGTGGAGGAGGT R:CCACCAACACCACACAAGAA | (TGG)5 | 52 | 169 |
| 198 | 17100 | F:TCATCTGAGGATTGGGCTGT R:GCAACTGATTAGGGTTGACCA | (CAT)5 | 52 | 169 |
| 199 | 17151 | F:TTCCCTGTGTGCCTCAGAAT R:AGTGCTCCAACGTGTCAGTCT | (TCA)5 | 52 | 174 |
| 200 | 17152 | F:CAAACGGAATAGGTAGAGAATGAAA R:AATGGCGTCTTGGAATTCAG | (GAA)5 | 52 | 174 |
| 201 | 17153 | F:CAGTTCCGATTCAGCAACAA R:TTGGTTGACTGACGTTCTTGA | (CAA)6 | 52 | 174 |
| 202 | 17162 | F:ATTTCCCCCATGGTTTGATT R:GTTGGCACAGCAACACACTT | (CAT)5 | 52 | 175 |
| 203 | 17170 | F:GGAGGACCACCACTTGGATA R:TGGGTCTTGGATTTGTTTGTT | (CTT)5 | 52 | 176 |
| 204 | 17172 | F:CCCAAGGGCTTTTTACATGA R:GACGTTGAATATGGGGATGAG | (CAC)7 | 52 | 176 |
| 205 | 17174 | F:CAAGCGCAATCAACAACAAT R:TAACTGACCCAAGGCCAAAG | (AAG)5 | 52 | 176 |
| 206 | 17176 | F:CCAAGGGAAACCAAGAGGTA R:AGTTTCGAAAGGCTCACTGC | (GGA)5 | 52 | 177 |
| 207 | 17178 | F:CCATTCACTCTGCTCTTCATCA R:GCAAGCGGAAGGAGTACTTG | (ATC)5 | 52 | 177 |
| 208 | 17182 | F:CGGGAGGTGAGCTTTATTGT R:TGCTCCCATGTTTCTTGACA | (TGT)5 | 52 | 178 |
| 209 | 17185 | F:GCAGCAATGGAAGCCTCTTA R:TTTGGCATCCTTCCAACTTC | (CTA)6 | 52 | 178 |
| 210 | 17187 | F:GCCATCACAATGCACAAAAG R:ATTGAGGCAAGAACGGAGAA | (AGA)5 | 52 | 178 |
| 211 | 17188 | F:TCAAAATCCAAGGGACATGA R:TGTCATTGTTGTTGTTGTTGTTG | (ACA)5 | 52 | 178 |
| 212 | 17194 | F:GGCTCAAGAATTTGCAGAGG R:CAACATCAGTAATAACCGCAACA | (TTG)5 | 52 | 179 |
| 213 | 17195 | F:GTCGGGAAACCCTCCTACTC R:CGCGTTCCTCGCTTCTATAC | (GCT)5 | 52 | 179 |
| 214 | 17198 | F:AGACCATGCCAACAACATCA R:GCGTCAAAAGAGACCTTCTTC | (ACA)5 | 52 | 179 |
| 215 | 17200 | F:TGAGTAGGGTCAAAGGAGACCT R:CCGTCAACATCAGCATCAAG | (TTG)6 | 52 | 180 |
| 216 | 17202 | F:CCCGTAGTGGTCGTGTTTTT R:TCTGGCCAGTAGGGATAGGA | (TTC)5 | 52 | 180 |
| 217 | 17204 | F:GGCTTGTTGACGGAAGAGAG R:TCCCCATACAAAGCATGAAA | (GAA)5 | 52 | 180 |
| 218 | 17207 | F:TCAAGTTTCAGAAGAAGAAGGAGAC R:CCATGGGGTTTTCACTTCTC | (AAG)5 | 52 | 180 |
| 219 | 17213 | F:TTCTCAACCGGCAATTCTCT R:GGTGTCTGGGGAATGTTTTG | (CAC)5 | 52 | 181 |
| 220 | 17215 | F:ATGTCAGACCTCGCCAACA R:CAAAAGTGACCTTCTTCCTCTCA | (CAA)5 | 52 | 181 |
| 221 | 17216 | F:TGGCCATAGATTTGTGTTTGA R:TCGCTTCTATGCCCTTGTTT | (AGT)7 | 52 | 181 |
| 222 | 17222 | F:TTTTCGTAGGCAATGGAACC R:TAAAACCCATGAGCGGGTAG | (TTG)6 | 52 | 182 |
| 223 | 17223 | F:AGAAGTTTGGCCCTGAGGTT R:GGAGGAAATGACGACCAAGA | (TCT)6 | 52 | 182 |
| 224 | 17227 | F:CCAGTGGGACAAGTGACAAC R:TCATGGTCTTCCCGCTTTAT | (ATA)5 | 52 | 182 |
| 225 | 17229 | F:GTGGCTCAGGAATTTGAGGA R:ATCAACAACAACCGCAACAA | (TTG)7 | 52 | 183 |
| 226 | 17232 | F:CGATCCTCAATTCACGTTGT R:ACCGTAATGGCTATCGTCGT | (TCA)6 | 52 | 183 |
| 227 | 17234 | F:CGATGGGTTCCTAGGTTGAA R:ACAACAACAACCGCAACAAA | (TGT)5 | 52 | 184 |
| 228 | 17239 | F:AGTGCTGTGCAACACGGTAG R:AATGTCGATCAGGCATCCTC | (ACA)7 | 52 | 184 |
| 229 | 17240 | F:GGAATCGGGTTAAAAGAGAGC R:ATCCACCGCAAAGAACAAAC | (TTG)5 | 52 | 185 |
| 230 | 17242 | F:CCAACTGGTGAACACAGAGC R:ACCACTGCATCTCCATTTCA | (TTC)5 | 52 | 185 |
| 231 | 17346 | F:TCTGGACTCAGCATTAGATTTTT R:GGGATTGGCACCTTCTGATA | (GAA)5 | 52 | 194 |
| 232 | 17347 | F:ACAAAGCAGCAGCAATAGCA R:ATTACGCGGGGGAAATTTAG | (CAA)6 | 52 | 194 |
| 233 | 17350 | F:AAAGCTTGTGAAAAAGAGGGAGT R:GGACTGTGAAGCCGAAGATT | (AGA)6 | 52 | 194 |
| 234 | 17351 | F:ATAGTTCCCGTTCCCAGTCC R:GTAACTGTCCTCCGCCTCCT | (ACC)5 | 52 | 194 |
| 235 | 17355 | F:AGCGTCTTCAGGTTCAGGAG R:TTTCGTTATGGGTTCGTTCC | (TTC)5 | 52 | 195 |
| 236 | 17357 | F:CGTACGGTTCGATGTGATACC R:CATCTTCCGAACCCATGAAC | (TGG)5 | 52 | 195 |
| 237 | 17359 | F:TCGTCCAAGTAGGCCAGTTT R:TGGTTGAGGAAGATCCATGTC | (TAC)6 | 52 | 195 |
| 238 | 17363 | F:GCTAGGAAGAGGGAGGGAGA R:CATCCTCTGTCTCGGCTGAT | (CAA)7 | 52 | 195 |
| 239 | 17366 | F:GGAGGCTTCGTGTCATAAGG R:TCTTCCTCTCAAAACTTTGGTGT | (ACA)5 | 52 | 195 |
| 240 | 17367 | F:TCTCAATCCCATCAGCATCA R:TGTCATAGGAATCGGGTCAA | (ACA)5 | 52 | 195 |
| 241 | 17369 | F:GGGCTCTCTCATTTCGCATA R:TAGTGACCCCGTTTCCTCAA | (TGA)5 | 52 | 196 |
| 242 | 17372 | F:AGGACATGCTCGCTCATCTT R:GGAGGAGGACTTCCTGGAGA | (CGA)5 | 52 | 196 |
| 243 | 17374 | F:AAGTTTCCGAGTCCCCTGAT R:TCGTAATCGTCAGCCTCAGA | (ATG)6 | 52 | 196 |
| 244 | 17375 | F:CAGGCTTTCACCGTCATTTT R:GGAGTGGATCGGAGATGAAA | (AGA)5 | 52 | 196 |
| 245 | 17376 | F:AACCGAGTCCACCATTCTTG R:TTGTGTTCAAGGTGGGAATG | (ACA)5 | 52 | 196 |
| 246 | 17377 | F:AGGGGTTCACGACTCCCTTA R:AACAAGAACAAGAACAACAACAATG | (TTG)7 | 52 | 197 |
| 247 | 17378 | F:TGCCATATCCAGCAAAAACA R:TTCAACCACCATCACCATTC | (TTC)5 | 52 | 197 |
| 248 | 17379 | F:TGGTGTGCTTCAGAAAGAGTG R:ATGCTCTTCTGGCAGCTTCT | (TCT)5 | 52 | 197 |
| 249 | 17382 | F:TCGCTAGACTTTGCGGTGTA R:TTTGCTCAACAAAAGGAATTACAA | (GTG)5 | 52 | 197 |
| 250 | 17383 | F:AAAGGAAACCACCACCAAAA R:GGTGGCTTTCGACTTTCCTA | (GAA)5 | 52 | 197 |
| 251 | 17385 | F:CAATTCAAACCCCATTTCTTG R:TTGTTGATGATTGTTGGTGTTG | (CAA)6 | 52 | 197 |
| 252 | 17386 | F:GCCCCCTATGGAAAGAAAAG R:CGTTGTTGTTACGGCTGCTA | (CAA)5 | 52 | 197 |
| 253 | 17391 | F:ACGAAGCCAAAAGGTGAAGA R:TCTGCGGGAAGAAGAATAGAA | (TTC)5 | 52 | 198 |
| 254 | 17392 | F:GCGCTGTCCGTGGTATTTAT R:CGACTCAAGGGTTCCGAAAT | (TGA)6 | 52 | 198 |
| 255 | 17393 | F:GATGAAATGAGGGGTTGGTC R:GGGGATCAAGTTCTCCATCA | (TGA)6 | 52 | 198 |
| 256 | 17394 | F:GCCAAAGACCAAAACGACAT R:TTGGTGATGAAATGAGCAAAA | (TCA)5 | 52 | 198 |
| 257 | 17396 | F:ACCCATGGCTTCTTGATTTG R:CCGCTACAGGGACAAAAGAG | (GTT)7 | 52 | 198 |
| 258 | 17402 | F:CTTCTTCTTCTGAGCGGAAAAT R:ATTGGATCAACCCCAAACAA | (TTC)5 | 52 | 199 |
| 259 | 17414 | F:TGTTGTTGCTGACGATGTTG R:TATGGGGGCTTTGCTAAGAA | (TGT)6 | 52 | 200 |
| 260 | 17417 | F:CAAACTCTGTCTCGGCTGAT R:CGGAGACCCATGTTGTTTCT | (GTT)7 | 52 | 200 |
| 261 | 17419 | F:TGGAAGGTTCATAGGTAAAACTCC R:TAGAAGGGAGGTGCCAAAAA | (CTT)6 | 52 | 200 |
| 262 | 17420 | F:GGGTTGCCTAGTGGTCAAAA R:TGCAAGCTAGGGTTTCATTG | (ATG)5 | 52 | 200 |
| 263 | 17421 | F:GGTCGTGTTTTTGGTCCTGT R:AGCTGCTCCACAACATTGAA | (ATG)5 | 52 | 200 |
| 264 | 17425 | F:GCTGAACCTTCAACCGAGAA R:ACCGCGATCAACAATAGGAC | (TGA)5 | 52 | 201 |
| 265 | 17426 | F:AAACCCTCCTCCACCACCT R:CTTTGTGCGCATGTTTTTGT | (TCC)5 | 52 | 201 |
| 266 | 17429 | F:AAATCCAACCCAAAGGTTCC R:GGAGTGCTGTCCGTGGTATT | (CAT)5 | 52 | 201 |
| 267 | 17432 | F:ACTGAAGCAGAAGGCGAAAA R:TCCTCGAGACCCACAAAATC | (AAG)6 | 52 | 201 |
| 268 | 17488 | F:CGATTCATGCTGCTGTTGTT R:CAGCATGTTTCGCCATAGTG | (AAT)5 | 52 | 207 |
| 269 | 17489 | F:TCACACGGTTTGTTCCAAAA R:TGTTCTTGCTGAGGATTTTTGTT | (AAG)5 | 52 | 207 |
| 270 | 17490 | F:GGAATGAAGGACGAGGATGA R:GATCCACACCCAACACATCA | (TTA)5 | 52 | 208 |
| 271 | 17491 | F:CCTTCTGGCACAAAGCTAGG R:AAATCTTTGAAAGGCTCACTGC | (GAG)5 | 52 | 208 |
| 272 | 17492 | F:AAGGAGCCAGAGGGAATGTT R:ATCCTTCCTTCCCAGCTGTT | (AGA)5 | 52 | 208 |
| 273 | 17493 | F:GCAGAAGGGCTGAAATGAAC R:TCCAGCTCCAACTCCAACTC | (TTG)7 | 52 | 209 |
| 274 | 17494 | F:GGACTGTGCAGCATGAATGT R:GTGTAGGGCTTGGAAGTGGA | (TGT)6 | 52 | 209 |
| 275 | 17498 | F:GCATCAGTTTCGGGAAGAAG R:GGTGTTTGTTTGTTGTTGTGGT | (CAA)5 | 52 | 209 |
| 276 | 17501 | F:CCTTCGAATTCAGCGAAGAC R:CGTCTACCACATCGGGAGAT | (TCT)5 | 52 | 210 |
| 277 | 17504 | F:ACCCAACAATTGGCATCAAG R:ATGGTCCCTTGGAGATGACA | (GAT)5 | 52 | 210 |
| 278 | 17522 | F:GCGGAGGGAGAACTGAATTG R:TGGCAGACGTACCCATAGTG | (AG)17 | 52 | 139 |
| 279 | 17527 | F:TGTGTGTATGTGTGCGTATGG R:GCCTTTTGCCAATTCTTCAA | (GT)6 | 52 | 140 |
| 280 | 17586 | F:CCTCTGTGTCCCCCTGTAGT R:GCGTGAGTGGGTGTGTATGT | (AC)8 | 52 | 147 |
| 281 | 17587 | F:CGGGAATTGAGATCGAAGAC R:GTTGCCTGCATCACCATACA | (GT)6 | 52 | 148 |
| 282 | 17589 | F:CAATTTGGGAGGGAAATTCA R:GATGCAATGCTTCGTTGATG | (GA)6 | 52 | 148 |
| 283 | 17592 | F:CTTGGGAAGCTCTGAAGGAC R:TCCAAGCTGAACCCAGAGAT | (AG)9 | 52 | 148 |
| 284 | 17594 | F:TCATTAGGGGCAAGATGACC R:AGGAGGGACGGCAGATACTC | (TG)6 | 52 | 149 |
| 285 | 17608 | F:GAAATGCCTCACTTGCAAAA R:TCCATTCCCACATATTTTCACA | (AG)6 | 52 | 150 |
| 286 | 17610 | F:CATCCTTTTGCTTCTCCCTCT R:GGTCGCCAGAAAAGATGTTG | (TC)13 | 52 | 151 |
| 287 | 17612 | F:TTTGTGGTTGTGGCTGAGTC R:CCTCATCCTTTCGGTTCAAA | (GA)14 | 52 | 151 |
| 288 | 17615 | F:GTCCCGGGTTTTGGTGTTAC R:GCATTGTTCTGGGAGGAGAG | (AT)7 | 52 | 151 |
| 289 | 17620 | F:GGCGTTTGAAACCGTTAAGA R:TCTTCCTTTGCCGCAATATC | (GA)6 | 52 | 152 |
| 290 | 17624 | F:GATACTCGCCACCGTGATCT R:GTTCTATCGGGCCTCAGCTA | (AC)6 | 52 | 152 |
| 291 | 17630 | F:CCTCGTTTCTCCCAAAACCT R:GGTTTTCAACCGTTGGTAGG | (CT)9 | 52 | 153 |
| 292 | 17632 | F:CATGGACTTGGGTTCAGGAT R:CGCTTTAACCACCGTGAGAT | (AT)6 | 52 | 153 |
| 293 | 17635 | F:CATCTCTCTCCTCAATCAACACC R:AGCAGGCAGGAAAGTGAGAG | (TC)6 | 52 | 154 |
| 294 | 17641 | F:GAACGCCTAAAAACGGAGAA R:AGGGGTTCTGGCTTCAATTC | (AG)6 | 52 | 154 |
| 295 | 17645 | F:ACCTTATTTTGGCCGCTCTT R:TAAGAGCGAACCAGGGTTGT | (TC)8 | 52 | 155 |
| 296 | 17649 | F:TCAAGGGGCAATAACGAGAT R:TGACGTAGCAGCGTGAAGTT | (GA)7 | 52 | 155 |
| 297 | 17653 | F:GGGTTTAGCCTCCCTTCCTA R:TCTGCAGGCTCAAGTCATTG | (GA)12 | 52 | 156 |
| 298 | 17656 | F:CGTTTTACTTGATTTTCTGCAACT R:ATCACGCAGCACATGTTTTT | (AG)8 | 52 | 156 |
| 299 | 17662 | F:AGAAGTTGCGTTGGGTGGT R:TGGAAAATACGGTGCAAACA | (GA)6 | 52 | 157 |
| 300 | 17670 | F:TCCAGACCCACTCATTCTCC R:GACTTTAGCACAATTTTAATCATACG | (AG)8 | 52 | 158 |
| 301 | 17672 | F:TTCTGAGGGACCCATCTCAC R:GATTGGACGTGGAGGAAGAA | (TC)6 | 52 | 159 |
| 302 | 17676 | F:ATGCCCTTAACAACCACACA R:GGCCTGCATTTTTGGTTAAA | (CT)6 | 52 | 159 |
| 303 | 17681 | F:TTACGTCAGCATCCGAACCT R:TTTGCGTGAGTTGTCTGACC | (AC)6 | 52 | 159 |
| 304 | 17683 | F:TTGAAGCAAAACCCTAATTTCA R:GCTCAAACCAATGGACACAA | (TG)6 | 52 | 160 |
| 305 | 17687 | F:CTTACACAAGCCACCGACAA R:GGTGCAGCTCGAACAAGAAT | (AC)6 | 52 | 160 |
| 306 | 17693 | F:CCACGTGTGGCATATCAAAG R:TTTGAAGGGTGGTTGGAGTT | (CT)6 | 52 | 161 |
| 307 | 17694 | F:CGGGTACTAAATATATGGCGAACT R:TGCGATGTTCGCTTCCTTAT | (CT)13 | 52 | 161 |
| 308 | 17695 | F:GCACACGCAGACACACACTT R:TGTGCTTTCTCATGCACAGG | (CA)8 | 52 | 161 |
| 309 | 17699 | F:TGTCTGTTCTCCCTTTCTGGT R:GCCAATCAATGGGCTTACAT | (TG)6 | 52 | 162 |
| 310 | 17706 | F:GCAAAAAGCTTCGGTGAAC R:CCTTGGAGAAGACCGTTGAG | (TC)7 | 52 | 163 |
| 311 | 17713 | F:AAAAAGGGGAAAGCAGGAGA R:TTGACTGTGAGGCTGGTTTG | (CT)10 | 52 | 164 |
| 312 | 17715 | F:ACCACACAAGGCTTCTCACC R:TTTTACCAGGGTCCCACAAA | (AC)8 | 52 | 164 |
| 313 | 17717 | F:TTGTGTGAATTACAGAGTGAGTTGA R:TGCTCCATAATCAAAGGAAAAA | (TG)6 | 52 | 165 |
| 314 | 17718 | F:GACGGTGGAAGTCGTATCGT R:GGTCACAGTGGGGAGAGAGA | (TC)6 | 52 | 165 |
| 315 | 17721 | F:GGGAGGCTTATTGTGCAAAG R:TCAAGCTTAGATTCTCCCTCAC | (AC)6 | 52 | 165 |
| 316 | 17724 | F:TTCTCTCTCTTTCTCTCTCTTTCTTTC R:CACACAACGCCTCACTGTTT | (TC)6 | 52 | 166 |
| 317 | 17727 | F:GGCCACATGTCCGATTCTAA R:GCGGTTTGGATGGTGATAAA | (CT)7 | 52 | 166 |
| 318 | 17729 | F:ATGCCGATGCATTATTCTGA R:GGCTAGATTTTTGGTTTTCTGTTT | (AT)6 | 52 | 166 |
| 319 | 17733 | F:TGCTTCAGCAATTGAAGTCTCT R:TGAGTTAAGTGTTTGCAGCAGAT | (TC)7 | 52 | 167 |
| 320 | 17736 | F:TGAGCTGCTGCTGGAATATC R:AGCATGCATTCTGCCTTTTT | (CT)6 | 52 | 167 |
| 321 | 17738 | F:TCACAAAAGCACTTAGGCTAAAAT R:CTCCTTTTTGAGCCAACCTC | (AG)8 | 52 | 167 |
| 322 | 17744 | F:AACCCTCTCACGCTCTCAAA R:AGCTTCTTCACGCCTTTCAC | (TC)6 | 52 | 168 |
| 323 | 17749 | F:GCTTTCTCGGTGACGAGCTA R:TTGCAACTTGGGATGAAAGA | (CT)11 | 52 | 168 |
| 324 | 17760 | F:TTGCCAAACGAGAGCAGTAG R:TGGTGATTTCCCTACGATTGA | (AG)9 | 52 | 169 |
| 325 | 17764 | F:GCGTGTTCTCTCGCTCTCAT R:GGAGGTCACCAACGGTAAGA | (TG)6 | 52 | 170 |
| 326 | 17772 | F:TCAAACCCACATGGGCTAAT R:GTGAGCGGAATCCACATTCT | (AG)9 | 52 | 170 |
| 327 | 17911 | F:TTGGTGACATGGTGGCTAAG R:AACCGGAAACCTGAGCTGTA | (TC)19 | 52 | 187 |
| 328 | 17913 | F:ATTAGCAGCACCCCATTCAC R:GCCTCATACTTCGTGGTTCC | (GA)6 | 52 | 187 |
| 329 | 17918 | F:GTCACGAACGTCGAATGATG R:CGCCAACACAAGTGGTTTTA | (AG)6 | 52 | 187 |
| 330 | 17944 | F:GAGAAGGGTTTGCATGTGGT R:TGATGACGTGGCAAAGGATA | (AG)7 | 52 | 190 |
| 331 | 17953 | F:GAGCTTTTGGTGGATGGTGA R:AACCCTAACCCCTTCCCTAA | (GA)6 | 52 | 191 |
| 332 | 17957 | F:GGCCAGACCAACAATGGATA R:GTTCGTCGGAGAAGAAGGTG | (AG)6 | 52 | 191 |
| 333 | 17982 | F:GGTTCGGGGAGTTAGGTGAT R:CGCAGGATGAATGGATGAAT | (AG)7 | 52 | 194 |
| 334 | 17983 | F:GGCAACCCCACCAATACTAA R:TCGCCAATGCTAATCTTTCA | (AG)6 | 52 | 194 |
| 335 | 18004 | F:CCTCCAATTTGACTTCAAAACA R:GCATCGCAACAACCGTATTA | (AT)6 | 52 | 196 |
| 336 | 18005 | F:GAACATTCCTACAAAGCACTAGAACA R:TTGTGTGGGGGAATTTTCAT | (AC)7 | 52 | 196 |
| 337 | 18039 | F:CTCGCGGGAGTCACATTATT R:GGAAGATAAGGGGAGGCATA | (TC)6 | 52 | 201 |
| 338 | 18041 | F:TCGTGAAGGGTGTCGGTAAT R:CACCCGAAATTTTCACCACT | (GA)7 | 52 | 201 |
| 339 | 18045 | F:TCCTTGCTTGTGTGCAACTC R:CTATTTACCCCTCCGATGGT | (AC)6 | 52 | 201 |
| 340 | 18050 | F:CCTTCATGCACTCAAGGAAAA R:TTTGACCGGAGAAGAAGGTG | (GA)7 | 52 | 202 |
| 341 | 18051 | F:ACAGACACACACGCAAGCTC R:GTGCGCTTGTGAAGGTACG | (CA)20 | 52 | 202 |
| 342 | 18061 | F:TCTGGACGATTGGAGTTGTTT R:CTCAAACCCTAAAACGCTCTG | (AG)6 | 52 | 203 |
| 343 | 18139 | F:TGGTTGAGTTCAAACCTCTCC R:GGCAACCCACGTTGTTTAAT | (A)10 | 52 | 120 |
| 344 | 18142 | F:TGGCCATTGTGATGGAAGTA R:GCTCCTCTCTTGAAACACGTTA | (T)11 | 52 | 121 |
| 345 | 18183 | F:CGTAAGGAAGCCGATGAAAA R:CCACAACCGTTGGGTTAAGT | (A)10 | 52 | 123 |
| 346 | 18232 | F:ACAAAACCCCTTGCTGAAGA R:TCAAAGTCTCATTTTGTCATTCTGA | (T)10 | 52 | 127 |
| 347 | 18234 | F:TCAAGCAATCAGCAGGAAGA R:GCCAGGAATTTGGTCTGAAG | (T)10 | 52 | 127 |
| 348 | 18239 | F:CTTCACAGAAATGGCGGAAC R:GCCTCATGTTGTAACTTCTGGT | (A)10 | 52 | 127 |
| 349 | 18241 | F:TGATGGTACACCAAGCACCT R:CGATGCACTAATTTTCGTTGTG | (A)10 | 52 | 127 |
| 350 | 18257 | F:TTTGCAAGCTTCTGATTTTGTC R:GGGAGAGGCAAAGAGAATTTG | (T)10 | 52 | 129 |
| 351 | 18261 | F:GCTTAAGGCCCAGGTTTCTAA R:CACTCCCTTAACTCATCCTTTTG | (T)10 | 52 | 129 |
| 352 | 18277 | F:TTTGCAAAGCGAGCCTAGAT R:GTTCTCCAGAAGGTGGGTCA | (T)10 | 52 | 132 |
| 353 | 18278 | F:ATCTCCGGACACACCCAATA R:TTGTGCATACGTTACGGAAAT | (T)10 | 52 | 132 |
| 354 | 18317 | F:CATGCTAAATGTCAAGTCAGGAATA R:TGCTGAAAATGGCCATAAAA | (A)11 | 52 | 135 |
| 355 | 18321 | F:CGCTTGGATGCCTACAAAGT R:TGAAACAGCAAAAGAACGAAAC | (A)10 | 52 | 135 |
| 356 | 18322 | F:TGAATGTGTCTCCCTGTAGCA R:CACCAATTGGAATCGGTAAA | (A)10 | 52 | 135 |
| 357 | 18348 | F:GTATCTCCTCCCCCGATTGT R:GCCAAACCGCTAAAGAAATG | (T)10 | 52 | 138 |
| 358 | 18350 | F:TCATTGGGCGGCTCTTATAG R:TCTGCTTTTGGCGAATTTTT | (T)10 | 52 | 138 |
| 359 | 18358 | F:CCTGAACCGATTTTGGTGAT  R：ATTCCGCCCTCTTTCACTTC | (A)10 | 52 | 138 |
| 360 | 18364 | F:TCATCTCCGTATGTTGGTTCC R:TTCACCCAAACCCATCCTTA | (G)10 | 52 | 139 |
| 361 | 18366 | F:TCATAGAAACAGGAGGGAAAACA R:CGTCCCGTTCATACATGTTC | (A)13 | 52 | 139 |
| 362 | 18388 | F:AGTCGGGAACAAGGGTAATG R:AAGTCGCCCTGATCCTCATA | (A)11 | 52 | 141 |
| 363 | 18389 | F:CCAGCTGAGAAAGGGTCATC R:TATGCATGCGTATGCTGTGA | (A)10 | 52 | 141 |
| 364 | 18390 | F:CGTCCTGCTCCATCCTATTT R:GGGTGGCAATCAGGTATGTC | (T)14 | 52 | 142 |
| 365 | 18393 | F:ACGCAAGTTGGCATGTTAAA R:CAGGCCCATCATGTGTGTAA | (T)10 | 52 | 142 |
| 366 | 18395 | F:TGTTCCATTGCAGTCCATGT R:TGCTCAAGTCAGTGGGTTCA | (T)10 | 52 | 142 |
| 367 | 18436 | F:TGAGTTGTGCCGGATATCAA R:TGGATCCAAAACTACAACAAATAAAA | (T)10 | 52 | 145 |
| 368 | 18446 | F:ACATCAGCCCTCCCCTTATT R:AAGAAAATGGCTCGGGTCTT | (A)10 | 52 | 145 |
| 369 | 18448 | F:TTTTTGGGTTTGTTTAAATTTTTG R:ACCCCACAGCTCGTACATTC | (T)12 | 52 | 146 |
| 370 | 18449 | F:TTCACATTGCATTGCATCAC R:GGAAAGGATGGTGGTTGCTA | (T)11 | 52 | 146 |
| 371 | 18451 | F:ATGGACGCGAACAGTCTTCT R:TCATTCTCACTCGGCACAAG | (T)10 | 52 | 146 |
| 372 | 18452 | F:TGCCTCGGTGTGTGTAACAT R:CATTGTTAGCCCTTACCAACA | (T)10 | 52 | 146 |
| 373 | 18461 | F:GGGGTGCAAGCATAGAACAT R:ATGATGCTGATTGGGAGGTC | (A)10 | 52 | 146 |
| 374 | 18463 | F:GGAGGAAACCCACACAATTT R:GGTATCGTCCTTCCGTTCAA | (T)10 | 52 | 147 |
| 375 | 18464 | F:TCCACCTCTAGGCTGGTTTG R:TGCAGAAAAGGCAACAGAGA | (T)10 | 52 | 147 |
| 376 | 18465 | F:GCGTAGGTAGCGTGCAAAGT R:AAATCTTCGTTGGCGGAAC | (T)10 | 52 | 147 |
| 377 | 18466 | F:TTACATCATGCCGTATGTCCA R:TCGCGATCACGATATTGGTA | (T)10 | 52 | 147 |
| 378 | 18467 | F:TCGCCCTTGTGGTACCTTAC R:AACCAGAATCGTGGGAATTG | (T)10 | 52 | 147 |
| 379 | 18471 | F:TGGAAGTTTGAGTTTGTATGAAAGA R:TGAACAGTTGATGAGGGTGAA | (C)10 | 52 | 147 |
| 380 | 18472 | F:GAAAACAGAAACCGCATTCA R:CGAATCGGATCTTCCTCAGA | (A)11 | 52 | 147 |
| 381 | 18473 | F:TCGATGTTCAGGAGCCAATA R:TTTCCAAGTTCTTAGTTTCTCTATTCA | (A)11 | 52 | 147 |
| 382 | 18476 | F:CATGCAAACCATCACGACTT R:ATCGTTCGTGACTCCAGACC | (A)10 | 52 | 147 |
| 383 | 18477 | F:AAAGCTCCAAATGCGACGTA R:ATGGCTTGTGCAGGTCTGA | (A)10 | 52 | 147 |
| 384 | 18478 | F:CGTCCGTTTTTGCGGTAATA R:TCACCTTTCAATCTCCATTAACC | (A)10 | 52 | 147 |
| 385 | 18479 | F:CCTCATAAAGCGCAGAAACA R:GGGTTGTTTGAGAGGGTGAG | (A)10 | 52 | 147 |
| 386 | 18484 | F:GAACCACGATGATTTCCAAAA R:AAAACTACCGCCGCTTATCC | (A)12 | 52 | 148 |
| 387 | 18487 | F:GCCCTCATTGCTTTCTTGTT R:GTCAAGCGCTGCTCTTCTTT | (A)11 | 52 | 148 |
| 388 | 18488 | F:CCACACATGATTTGACAAAATG R:CAGTGAATGACAAGGCAGACA | (A)10 | 52 | 148 |
| 389 | 18489 | F:CGGTTGTTCATCCCATTTTC R:GGGTGTCGGGACGATATCTA | (A)10 | 52 | 148 |
| 390 | 18491 | F:TCTCCCCTGAACCACTTCAC R:ACTGAATGCTTCGCCATTCT | (A)10 | 52 | 148 |
| 391 | 18492 | F:TCACAATGCCAAATGTTCAA R:GGACAAAGGCCAAATGGTAA | (A)10 | 52 | 148 |
| 392 | 18497 | F:AAAGCCATTCCAACCCAAAC R:GGGAGTCCCTGGATTTGATT | (A)10 | 52 | 149 |
| 393 | 18499 | F:GATGCCAATACTTTAGCAAGAGC R:TCAGGGAAAAGCGAAGAGAA | (A)10 | 52 | 149 |
| 394 | 18500 | F:AACCATGGCATTCCAAATGT R:ACTGTCTGCAGAGCGGATTT | (A)10 | 52 | 149 |
| 395 | 18501 | F:TACAAGGCGGTGGTTAGTCC R:CCGAATAGCATGTTTAGTACAGG | (A)10 | 52 | 149 |
| 396 | 18502 | F:GCTTATAGCAAAGAAGGGAGGA R:TAATCGGGGCCAATCAATAA | (T)11 | 52 | 150 |
| 397 | 18503 | F:CCCCCTTCTCGAGCTTATTC R:AAGGAGCAGCAGCATTTGTT | (T)11 | 52 | 150 |
| 398 | 18504 | F:TCTTTGGCACTTTTGCTCTG R:TCCTTGTGTGGGGGAATAAA | (T)10 | 52 | 150 |
| 399 | 18505 | F:ACCTTGAGACGGTGGTGAAC R:TTTGATTCGATTTGCACTGG | (T)10 | 52 | 150 |
| 400 | 18506 | F:TTGGATGGTGATGAGGAAGA R:GAAACGCTATGGGGAACAAC | (T)10 | 52 | 150 |
| 401 | 18514 | F:TCACCAGACCGACTCAGGAT R:CGCGTATGCCACATTATTTC | (A)10 | 52 | 150 |
| 402 | 18518 | F:GGGCATACTTTCATATTACCCTTA R:CCTTTTCCCGAATTCCATTA | (A)10 | 52 | 150 |
| 403 | 18520 | F:CCCGAGTCCATGATCCTTTA R:CCATTTCGTTTGCATTTGTT | (T)14 | 52 | 151 |
| 404 | 18523 | F:TGGAGGTGTGTCGTGAGTTT R:CATTTTCCCACCCCTAATGA | (T)10 | 52 | 151 |
| 405 | 18527 | F:ACGACGCAGATGCCTATTTT R:TTCGAAATGCAATCCCTTGT | (T)10 | 52 | 151 |
| 406 | 18530 | F:GGTGGTAGTTTCGAAAGGGAAC R:TCGTAGCTCGCCTCTTTCAC | (G)10 | 52 | 151 |
| 407 | 18531 | F:TTCATGCGAGAAAGGCTGTA R:TGTCAGCTGCAAGCAGATTT | (A)11 | 52 | 151 |
| 408 | 18532 | F:GACTTCCATCACCCCCATAA R:ATCGACCCCTTCTTTCCTGT | (A)11 | 52 | 151 |
| 409 | 18535 | F:CGTCAAAACTGTATCGCAAAA R:TCGGTTTTAGCTCCTTTTACTCA | (A)10 | 52 | 151 |
| 410 | 18539 | F:AGAGCCGTGTCAGGAACATT R:TGGCTGTGTCAAGCTACTGG | (A)10 | 52 | 151 |
| 411 | 18540 | F:CACTACAAAGCGGGAACCAT R:ACTTCCTGGCCAAATGATGT | (A)10 | 52 | 151 |
| 412 | 18560 | F:CGATGCAAACCAGATTCCTC R:CCGCCAGTCGACTCATTACT | (A)10 | 52 | 153 |
| 413 | 18594 | F:ATTCTGACCGCTCGACGAC R:TCGCCCAAAAACCATATCTC | (A)10 | 52 | 155 |
| 414 | 18596 | F:GCCTTGCTACCCATGTTGTT R:AACAGCGGCCTAAAACCTTA | (A)10 | 52 | 155 |
| 415 | 18601 | F:AAAGTGGGTGCTGCTCATCT R:GGTCAGTTCCAAACCACCAG | (T)10 | 52 | 156 |
| 416 | 18602 | F:GTGCATGTGAGTGGGTGAAG R:TCAGCCTCTCTGCTGTTTCA | (T)10 | 52 | 156 |
| 417 | 18640 | F:CGGTTGGCCTACTTGTCTTC R:CTGGCGGAGACATGAATTTT | (A)10 | 52 | 158 |
| 418 | 18641 | F:CCCCAAAAGCCTGTTATGAA R:TCACACATGCAGGATCAGGT | (A)10 | 52 | 158 |
| 419 | 18642 | F:GTGGTCCTGCATTGGAAAGT R:AAAATCAACCAAAATGGGTGT | (A)10 | 52 | 158 |
| 420 | 18643 | F:CTCCGGATACACCCAAATAG R:GCCCTTCAAAACCCAAAAAT | (A)10 | 52 | 158 |
| 421 | 18646 | F:GCTCTCTAGCCATCCCTCCT R:TCTGACGATTTGTTGGTGGA | (T)10 | 52 | 159 |
| 422 | 18648 | F:CGTTGGCTCCATTTTGATTC R:TGAAACCTAGAAACTGACATTTTGA | (T)10 | 52 | 159 |
| 423 | 18649 | F:TGAGACAAGTACATGGGGATCA R:TATTCGGGGAGAGGAAGGAT | (T)10 | 52 | 159 |
| 424 | 18703 | F:GCTTTAAAGGTGGGAGCATTC R:TGATGTACAAACAATATGATGCAAAC | (T)10 | 52 | 163 |
| 425 | 18704 | F:AAGAACGGAAAGCGGTCTCT R:ACCCGTCTGCCTCAACTTTA | (T)10 | 52 | 163 |
| 426 | 18705 | F:TCGCGTAGGTTGACTTGTTG R:GGTGGGTGAAAGAACGTCAT | (T)10 | 52 | 163 |
| 427 | 18707 | F:TTGGAAGTTTGGTCACAAAATG R:TATTGCCCATGAGCGTATCA | (A)10 | 52 | 163 |
| 428 | 18723 | F:GTCACAGGCAGCATGACACT R:CCAGCCATAGATGCACAAGA | (T)10 | 52 | 165 |
| 429 | 18730 | F:ACTCACAAAACCGCACACAC R:CGGCGAAGATGAAAGGATTT | (A)11 | 52 | 165 |
| 430 | 18734 | F:GGCCTTGCTTCACTCACCTA R:CGAAGATCTCCCTTTTTAAACTTC | (A)10 | 52 | 165 |
| 431 | 18747 | F:ACCGCAAGAACCACAAGAAG R:TCCAGATCCTTTTTCGGTGA | (A)11 | 52 | 167 |
| 432 | 18750 | F:CACCTCAATGCGCAAGTCTA R:GGGGCTAACCCTGCTAATGT | (A)10 | 52 | 167 |
| 433 | 18751 | F:CATTTTTGTTGAATCCATTTTCA R:GGTGGTGGAGCTTTAAGTGG | (A)10 | 52 | 167 |
| 434 | 18752 | F:CACCATCAATAACATACAACCAAAA R:AAAGGGGGTACCGATTTCAG | (A)10 | 52 | 167 |
| 435 | 18753 | F:GCAGCCTGCAGTAAAACACA R:AAACTCGGTTGCTGCTTGAT | (A)10 | 52 | 167 |
| 436 | 18754 | F:TGGGTGGGAAATGAAATGTT R:CAGGCGCTCTCTTCGATTC | (A)10 | 52 | 167 |
| 437 | 18755 | F:CTCCCCAACGGAAATTCTTT R:GGTCAATGCTTTGTGAAAGGT | (A)10 | 52 | 167 |
| 438 | 18757 | F:CGGAAGGTGGTGACGTTAAT R:CACTCAGCAACGCTCTCTCA | (T)11 | 52 | 168 |
| 439 | 18758 | F:TTGACCACCATCTCCAATCA R:TTTGAGTTGAAGAATAAATTCACTTG | (T)10 | 52 | 168 |
| 440 | 18780 | F:TTGATCATCCATTGCACTCC R:TTCCCTTCATTCGTGAGGTC | (A)11 | 52 | 169 |
| 441 | 18782 | F:GCTCCATCCCACCCTATTTT R:CGTGTTCCTTGCACTCATGT | (A)10 | 52 | 169 |
| 442 | 18783 | F:GGGATTGGTCCCTTGGATTA R:CATGGCAAGAACCAAGAGTTT | (A)10 | 52 | 169 |
| 443 | 18785 | F:TTGTGGAAGGAAAGGTAATCAA R:CTCGTTCCATCTCGGCATTA | (A)10 | 52 | 169 |
| 444 | 18791 | F:TTCTTCTAAACTTACTCTTGCACTTTC R:TTTGCTTCCCCATCTGATCT | (T)10 | 52 | 170 |
| 445 | 18792 | F:TCGCATTCTTTACACAAAACTCTT R:CCAAATATCAGCGTGCAAAA | (T)10 | 52 | 170 |
| 446 | 18793 | F:TTCGCACTTGAGGGGATATT R:GCTCAAGAGCAACCACACAA | (T)10 | 52 | 170 |
| 447 | 18906 | F:GCAAATCCTTTTTCCCCAGT R:CGTCCCGATTCGTTCTCTAA | (A)12 | 52 | 177 |
| 448 | 18907 | F:AGTGCCGAGTGAAGGCAAAT R:TTGACTTAGGGCAAACATGAAA | (A)11 | 52 | 177 |
| 449 | 18908 | F:CAGCCTAGTGGTTTGGCTGT R:TTACCTGGGGTGAGCACTTC | (A)10 | 52 | 177 |
| 450 | 18909 | F:ACGGGAATGCAAAATGAAAG R:GCCTCTGAAATGGAGGGATA | (A)10 | 52 | 177 |
| 451 | 18911 | F:CCAAATGTCTGGGCTTCAGT R:GAGTGCGAAAGCATGTTCAA | (A)10 | 52 | 177 |
| 452 | 18912 | F:CACCACCATGGCTATCATCA R:CGGTCGTCAGAGACGGTAGT | (A)10 | 52 | 177 |
| 453 | 18913 | F:CATTCATCATCCAAACACAGC R:TAAGCGGTATCGTGGGTCTC | (A)10 | 52 | 177 |
| 454 | 18914 | F:AACAATGGACCGGTGAGAAC R:TCGAGAGTGTGTGGGTGAGA | (A)10 | 52 | 177 |
| 455 | 18927 | F:GGGGTCGAGAGGAACAAAAT R:TGTAACCGCTTGCCTCCTAC | (T)10 | 52 | 178 |
| 456 | 18930 | F:ATGTAGTGCGGTCCGGTAAG R:GCAGTTCAATCTACCCGAGAA | (A)15 | 52 | 178 |
| 457 | 18932 | F:GAAGCAGGAGTTTGTCCTTGA R:GATGGGTGCACACTTGACAC | (A)11 | 52 | 178 |
| 458 | 19071 | F:CACGACGTCGAAGTTGACAC R:CACGCGTCTTCAAAGAGAAA | (T)10 | 52 | 187 |
| 459 | 19072 | F:TCCTCTTGTTTGCATTAGCA R:ACCTTCAACCATGCAACCTC | (T)10 | 52 | 187 |
| 460 | 19074 | F:GGGGGTAAGAAGGGAATTGA R:TTAGGCCAACAACCAACCTG | (G)11 | 52 | 187 |
| 461 | 19077 | F:TCACATGACCAGGGATTCAG R:AAGTTTGATGGCGACTCCAC | (A)12 | 52 | 187 |
| 462 | 19078 | F:ATGGAGGAACCCGGAAAAC R:GAGGGTGAAGAATCCACAAAA | (A)11 | 52 | 187 |
| 463 | 19081 | F:GCAAAACCAAAGCAAACCTT R:GCGTTGGGATCAAAGACAAT | (A)10 | 52 | 187 |
| 464 | 19082 | F:ATGGGAGGGAGAGAAATGGT R:TTTTCTCGCTTTTGCCAACT | (A)10 | 52 | 187 |
| 465 | 19146 | F:AGACCTGCAGAGCCTGTGTT R:TCGGAGCTGTATTTGCTTCA | (A)10 | 52 | 191 |
| 466 | 19147 | F:CCGCTACAGACCGGTATCAC R:CTTTGTGCCCTGGGTGTAAT | (A)10 | 52 | 191 |
| 467 | 19148 | F:GTACTCCCCCTCAAGGGAAC R:TGCTGCTACTACTGTTGGTGCT | (A)10 | 52 | 191 |
| 468 | 19149 | F:TCCCACTTTCACGATGTTCA R:GCTCCATCACGGTGAGTTTT | (A)10 | 52 | 191 |
| 469 | 19150 | F:AGGACGGGGGTAGAAGAGTC R:GCAAATGCAGCCTCAAACAT | (T)12 | 52 | 192 |
| 470 | 19151 | F:TCCCACCACAAGACATTCAA R:GGTGAGGCTGACATGGAGAT | (T)11 | 52 | 192 |
| 471 | 19152 | F:GGATTTCGACTCTCGCAGAT R:GGTGAACTTTTGGGTGATGA | (T)10 | 52 | 192 |
| 472 | 19153 | F:GATTGGGGAGAAAGCAGGAT R:GGAGAGGATGCAAAGCAAGT | (T)10 | 52 | 192 |
| 473 | 19154 | F:GGCATCTCATTTCGGTTGAT R:GAAGGCAGTGGAACGGTATT | (A)11 | 52 | 192 |
| 474 | 19184 | F:TATTGGCCCAGGCATCTTAG R:CGTCTTCTTGTTCCCACCAT | (T)10 | 52 | 194 |
| 475 | 19188 | F:TTTCCTCGATTCTCGTGTCC R:AAATTGCCACCCATTTACCTT | (T)10 | 52 | 194 |
| 476 | 19191 | F:CGCTGCCACCATCTACAAG R:GCAGCTGGCACTGAAATAGG | (A)10 | 52 | 194 |
| 477 | 19192 | F:CGAAAACAAATTGTGATTTTACCA R:TCTCCTCGGGTTAGTGATCC | (A)10 | 52 | 194 |
| 478 | 19193 | F:CACAAAAATTGCAGCCAACA R:TGGCTTTTGCTGCTCTTAGG | (A)10 | 52 | 194 |
| 479 | 19195 | F:CAGCATGCAAACATAACAACA R:CGACTAGGATGCTGCCATTT | (A)10 | 52 | 194 |
| 480 | 19198 | F:TATGACGCTGGACCATCTGA R:TTTTAATGGCCTTTGGTGAA | (T)10 | 52 | 195 |
| 481 | 19199 | F:CGATGAACCTTGGGAAAACA R:GCCGAAGTTTCCAAATTCAA | (T)10 | 52 | 195 |
| 482 | 19200 | F:TGGCTAAGGAACAAGCAGAA R:TCCAACTCAACCAACCGTAA | (T)10 | 52 | 195 |
| 483 | 19201 | F:ACTGGGCGCACTATAGCAGT R:CAATCCGTTTCTCCCTTCTG | (G)11 | 52 | 195 |
| 484 | 19252 | F:CAATATTGATCGGAATTTGTTTC R:TGCGGTTTGATTGAGTTTGA | (A)10 | 52 | 199 |
| 485 | 19253 | F:TAGGAGGCGGGAGGAGTATT R:CGATTCCGTTGCTCTTCTTC | (A)10 | 52 | 199 |
| 486 | 19259 | F:GAGTGTGCATGAGATTGAGGAG R:ACGACATGTTCGTCGTGCTA | (A)10 | 52 | 199 |
| 487 | 19261 | F:GGGGAACACTCGTTCCTTCT R:GGTGGCAACCAGAGAAAAAG | (T)10 | 52 | 200 |
| 488 | 19311 | F:CAGGGATGCACATGAAGTTG R:TCTGCCTGCACAAAACATTC | (T)12 | 52 | 204 |
| 489 | 19312 | F:GTCGGCTCACTCGTCTTTTC R:TGGAAATCATCGTGGTTCTG | (T)11 | 52 | 204 |
| 490 | 19313 | F:GACCGTCCGAAACTTGGTAA R:CCCATATAGAAAAGCATTCCCTTAG | (T)11 | 52 | 204 |
| 491 | 19314 | F:CACGTGGACATGAAGGTGAC R:TGTTGATGGATTCGTGTTCC | (T)10 | 52 | 204 |
| 492 | 19315 | F:TCTTGGTGTGTGCTTGTCGT R:ACCCATGCCTCTAACCCTCT | (T)10 | 52 | 204 |
| 493 | 19317 | F:CCCTCTAGCACTATTCCAGCTC R:GGAAGAATGGTGTTTTGGTCA | (T)10 | 52 | 204 |
| 494 | 19321 | F:TCAAATTCCTGCGACCCTTA R:AGCGTTGGGAGAGAGTGAGA | (A)10 | 52 | 204 |
| 495 | 19322 | F:TTCCGCCATGTTAGGTTTTT R:ACTTCCCGAAATGTGGACAG | (A)10 | 52 | 204 |
| 496 | 19335 | F:CTGCAGAGTAGCATCAATGACA R:ATTGATGACAAGGCGACCTC | (A)10 | 52 | 205 |
| 497 | 19336 | F:TTACACGAGCTTGGCAAATG R:TCCCATTGATTGTGGATGTG | (A)10 | 52 | 205 |
| 498 | 19337 | F:AGCGACCCTAATTGGTCAAG R:GTCTGACGCGACACCTACTG | (A)10 | 52 | 205 |
| 499 | 19338 | F:GGAACACGAGCCTCAGAAAC R:CCGGAGGTGCATCTCTGTAA | (A)10 | 52 | 205 |
| 500 | 19339 | F:TGCGATCCATTGGACATAGA R:GAAGTCGAGTAGGCGGATTG | (T)13 | 52 | 206 |
| 501 | 19340 | F:GCTGAATAGGGATCATGTGAAA R:GCTGCTTGCACTGCTTAGAA | (T)11 | 52 | 206 |
| 502 | 19341 | F:TGTCTATTGAGAAAGTGGAAAAGTTC R:CCCGGCTTCTCTTCCTATCT | (T)10 | 52 | 206 |
| 503 | 19342 | F:TCCATGGAGTGTGCCATTTA R:CAAATTGGATCCCAAGTCAAA | (T)10 | 52 | 206 |
| 504 | 19345 | F:CGTAGGGTGTTTGAAGGATCA R:CCCGCGAATCACTAAATTGT | (T)10 | 52 | 206 |
| 505 | 19346 | F:CGGTTCAGGCATTTTCTTGT R:CAGAAGAGGGGCATTTGGTA | (T)10 | 52 | 206 |
| 506 | 19347 | F:TATGTCCCGGAGAGTGATCC R:CATTCACCCACTTGGCCTAT | (T)10 | 52 | 206 |
| 507 | 19348 | F:CAGCACCAGAACAACTTTCAA R:GAAATGGAGAAGGCAGGTGA | (G)10 | 52 | 206 |
| 508 | 19350 | F:TCCACCCTGAAAGATTCCATA R:GGGCCTTCTTTCTGATGTGT | (A)11 | 52 | 206 |
| 509 | 19351 | F:CACAACAAGTCTCATCCTAAGCAC R:CTCATCGAAGACCCTTGGTTA | (A)10 | 52 | 206 |
| 510 | 19352 | F:TCCAGACATATCCAAAGTGTCAA R:GAGCGGGATCATAGGACTCA | (A)10 | 52 | 206 |
| 511 | 19354 | F:TTTGGTGATGTTGGGATGTC R:CTGTGAACGCAAGCTCAAAC | (A)10 | 52 | 206 |
| 512 | 19355 | F:TGCTTTGTATCTGCTTCCTCAA R:CTTGCTGAATCTGGCATGAA | (A)10 | 52 | 206 |
| 513 | 19356 | F:GCATGCATATCGAAGGAACA R:CAACACTGAAAGCCGAAACA | (A)10 | 52 | 206 |
| 514 | 19357 | F:ATTTCCCATCCCACCTTTGT R:GAGTCGATGGAAAGGGTGAA | (A)10 | 52 | 206 |

Note: F= forward primer; R= reverse primer, *T*_a_= annealing temperature.
