# Supplementary material for: High-Throughput Development of SSR Markers from Pea (Pisum sativum L.) Based on Next Generation Sequencing of a Purified Chinese Commercial Variety
Source: PLoS One. 2015 Oct 6;10(10):e0139775. doi: 10.1371/journal.pone.0139775 (PMC4595016; doi:10.1371/journal.pone.0139775)
Supplement: S4 File — (DOCX) [file pone.0139775.s004.docx]

**S4: Supplementary Table 3 Twenty two distorted segregation markers**

| **No.** | **Marker Name** | **Primer sequences(5’-3’)** | **Repeat motif** | **Ta(℃)** | **Expect Size（bp）** |
| --- | --- | --- | --- | --- | --- |
| 1 | 16237 | F:GCAAACGAAGCAGGCTTATC R:TTGGCTGATCCTGAAACTGA | (CATTTC)5 | 52 | 152 |
| 2 | 16410 | F:AAGGTCATGCTTCTTCATCTCT R:GGGTGAGGTGTTATGGCACT | (TTG)6 | 52 | 125 |
| 3 | 16433 | F:CACCGCAAACATAGCAAAAA R:TCTCATAGCTGCGAGGTTCA | (GAA)6 | 52 | 127 |
| 4 | 16437 | F:TTGTTTTTGTTGTTCTTGTTGTTG R:TTTTCGGGTTTTGCTTATGG | (TTG)7 | 52 | 128 |
| 5 | 16531 | F:AACCATGGGTTTGGTGTGAT R:CACATCCAATTCACCGTAAAGA | (TGA)6 | 52 | 139 |
| 6 | 16603 | F:ATGTGGTGGTGGTGCTACAG R:GTTACCGCTTTCGGATCAAC | (TTG)6 | 52 | 148 |
| 7 | 16899 | F:GCCTCCTTCGATACCAGATG R:GCCTCCAAAAGCTCCAAAAT | (AG)8 | 52 | 135 |
| 8 | 16914 | F:AACCTCGAGCAACAACAGGT R:TTAGGTTGGCGTTTTTGGTC | (ATC)6 | 52 | 149 |
| 9 | 17158 | F:CTCCCGAGTCTTGGCTAATG R:AGGCGCTCATAAACAGTTCC | (TTG)6 | 52 | 175 |
| 10 | 17193 | F:CACAGCCATACCCAAGTTACAA R:GGTTGCGAGGGATGAGAATA | (AAC)5 | 52 | 178 |
| 11 | 17219 | F:TCATGTGCATGTGATGAAGAAA R:GGTGTACCCATGTGCCATTT | (AAC)9 | 52 | 181 |
| 12 | 17225 | F:GTTGCAAGCTGCTACCATCA R:AGACGGATCCAACAATCTCC | (CCT)5 | 52 | 182 |
| 13 | 17360 | F:CGGTGACATTTGTTCGTCTG R:CGGTTGCATCTCTCAAACCT | (GTT)5 | 52 | 195 |
| 14 | 17384 | F:AGTAGCGGTGTGTGGTTGTG R:GGGAAGAAAAAGGTTGGAAGA | (CAT)6 | 52 | 197 |
| 15 | 17585 | F:AAAGCAGTGCATGCAACAAT R:CAACCACACTGCAATAACAGG | (AG)7 | 52 | 147 |
| 16 | 17593 | F:CATCCTCCTCCTCCATACCA R:TCATCATCAATGCAAAGGACA | (AG)6 | 52 | 148 |
| 17 | 17773 | F:TTCCACACGAGGCTATTTTC R:TGCAAAAGCGACATCTTGAC | (AG)7 | 52 | 170 |
| 18 | 17952 | F:TCATCCAATATATCAAACATACCTCT R:TGCTCGATCGTGTTTTTCTG | (GT)7 | 52 | 191 |
| 19 | 17989 | F:CAGAGCCGGAGTTCTGGATA R:TTTGGTTGACATTAGCACATGA | (GA)12 | 52 | 195 |
| 20 | 18260 | F:AACCTTGAAATGGAGGTACATGA R:GACCATGATCGGATGTTGTG | (T)10 | 52 | 129 |
| 21 | 18529 | F:GAATGTGCGTCCAACATCCT R:AGATTTTGATGCGGAAGAGC | (T)10 | 52 | 151 |
| 22 | 18562 | F:TTCTTCTGCTGCTGCTCAAA R:AAAACAAAAACCACAACCAAAAA | (T)11 | 52 | 154 |
